# Tandem utilization of CO_2_ photoreduction products for the carbonylation of aryl iodides

**DOI:** 10.1038/s41467-022-30676-y

**Published:** 2022-05-26

**Authors:** Yuan-Sheng Xia, Meizhong Tang, Lei Zhang, Jiang Liu, Cheng Jiang, Guang-Kuo Gao, Long-Zhang Dong, Lan-Gui Xie, Ya-Qian Lan

**Affiliations:** 1 Jiangsu Collaborative Innovation Centre of Biomedical Functional Materials, Jiangsu Key Laboratory of New Power Batteries, School of Chemistry and Materials Science, Nanjing Normal University, Nanjing, 210023 PR China; 2 School of Chemistry, South China Normal University, Guangzhou, 510006 PR China

**Keywords:** Photocatalysis, Coordination chemistry, Chemical synthesis, Photocatalysis

## Abstract

Photocatalytic CO_2_ reduction reaction has been developed as an effective strategy to convert CO_2_ into reusable chemicals. However, the reduction products of this reaction are often of low utilization value. Herein, we effectively connect photocatalytic CO_2_ reduction and amino carbonylation reactions in series to reconvert inexpensive photoreduction product CO into value-added and easily isolated fine chemicals. In this tandem transformation system, we synthesize an efficient photocatalyst, NNU-55-Ni, which is transformed into nanosheets (NNU-55-Ni-NS) in situ to improve the photocatalytic CO_2_-to-CO activity significantly. After that, CO serving as reactant is further reconverted into organic molecules through the coupled carbonylation reactions. Especially in the carbonylation reaction of diethyltoluamide synthesis, CO conversion reaches up to 85%. Meanwhile, this tandem transformation also provides a simple and low-cost method for the ^13^C isotopically labeled organic molecules. This work represents an important and feasible pathway for the subsequent separation and application of CO_2_ photoreduction product.

## Abstract (web summary)

A Ni-based MOF catalyst is reported to facilitate the photocatalytic reduction of CO2 to CO, a low-value product. In tandem, the as-produced CO is used as a reactant in the Pd-catalyzed carbonylation of aryl halides and other fine organic chemicals.

## Introduction

Since the onset of the industrial revolution, the anthropic excessive emission of carbon dioxide (CO_2_) has resulted in serious environmental problems (e.g., the greenhouse effect)^1–4^. In order to effectively reduce the atmospheric concentration of CO_2_, the safe, green and sustainable photocatalytic CO_2_ reduction reaction (CO_2_RR) technology is currently regarded as one of the most promising solutions, due to the direct photoreduction of CO_2_ into value-added carbon-based chemicals with renewable solar energy^5–8^. Although the photocatalytic CO_2_RR has gone through rapid development and obtained many great achievements in the past few decades, it still confronts with the following severe issues^9–13^: (1) the resultant major reductive products (e.g., CO, HCOOH) have low practical value^14–17^; (2) the reductive products suffer from low yield (often in nanomolar/micromolar scale)^18–21^; (3) the incomplete photocatalytic CO_2_RR results in a high-cost and difficult product isolation process. Therefore, these problems greatly affect the feasibility and economic usefulness of the subsequent separation and application of photocatalytic CO_2_ reduction products. In the current situation, the efficiency of the photocatalytic CO_2_RR and the yield of reductive products cannot be significantly improved, so we consider that reconverting these low-value and low-yield reductive products into chemicals with high-value and mature separation techniques as rapidly and wholly as possible, through the further tandem transformation technology, is probably a feasible strategy toward practical application.

**Fig. 1:**
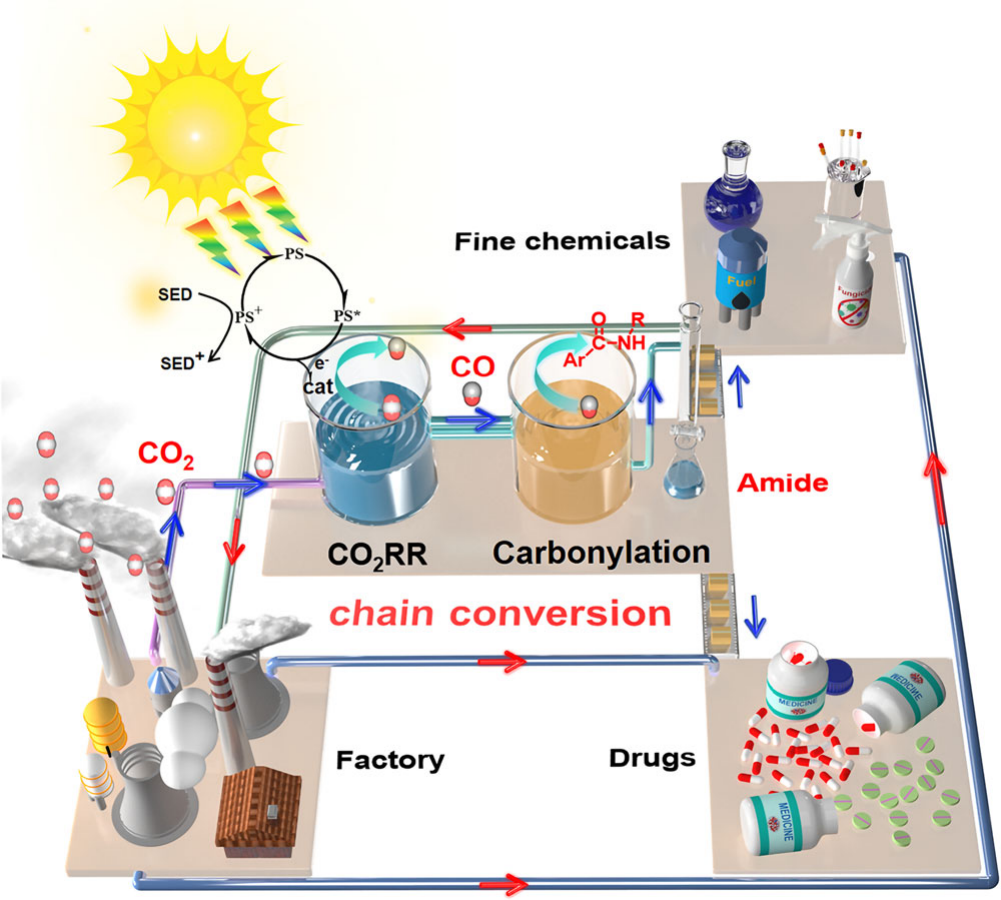
The tandem coupling of photocatalytic CO_2_RR and carbonylation reaction for the utilization of CO_2_ photoreduction product. PS photosensitizer, SED sacrificial agent, cat catalyst.

Carbon monoxide (CO) is an essential organic feedstock that has been widely used in the industrial preparation of fine chemicals including alcohols, aldehydes and carboxylic acids, which have owned the relatively matured separation technology and industrial application^22–26^. For example, the palladium-catalyzed carbonylation reaction can use CO to effectively prepare high-value photosensitive, conductive materials and important drug molecules through a simple one-step reaction^27,28^. This type of reaction occupies a vital position in chemical production and the pharmaceutical industry. Moreover, such reactions usually show a high conversion (80–90%) and a rapid and complete conversion process, even under the low CO concentration^29,30^. However, during the carbonylation reaction and its industrial scale-up production, using a large amount of highly toxic, colorless, and odorless CO gas as the direct reactant is still a potential threat^31,32^. Besides, the conversion of CO is likely to be remarkably reduced if the majority of CO source has not been used in the reaction completely and timely. Under these circumstances, if CO can be prepared through a safe, effective and green reaction method, and then serve as a reactant to participate in the palladium-catalyzed carbonylation reaction continually, a cascade catalytic reaction will be realized, which can extremely alleviate the aforementioned issues^33–36^. Inspired by this point, we consider that connecting photocatalytic CO_2_RR (for CO_2_-to-CO conversion) and carbonylation reaction to conduct a sequential catalytic reaction probably can achieve the following advantages: (1) CO_2_ photoreduction reaction can provide a safe, green and effective way to gradually generate low-concentration CO reaction source for the carbonylation reaction; (2) the produced low-concentration CO derived from photocatalytic CO_2_RR can be sufficiently reacted with the excess carbonylation reactant in a stoichiometric ratio to favor the efficient reaction conversion; (3) more significantly, such a tandem reaction can finish the efficient transformation of low-concentration and low-value CO product (from CO_2_ photoreduction) into high-value and easy-to-separated organic chemicals, which represents a feasible path for realizing the separation and practical application of photocatalytic CO_2_RR products in the true sense (Fig. 1).

In this work, we design and synthesizes two stable metal-organic frameworks (MOFs), [M_3_(5-AD)_4_(CO_2_)_2_] (CH_3_COO^−^)_2_ (NNU-55-M, M = Co, Ni, 5-AD = 5-Azabenzimidazole), both of them can serve as crystalline catalysts for photoreducing CO_2_ to CO under visible-light irradiation. Among them, the CO product selectivity (12.4%) and output (12.6 μmol, 630.0 μmol g^−1^, 39.4 μmol g^−1^ h^−1^) of NNU-55-Co are low under photocatalytic CO_2_RR conditions, but the competitive hydrogen evolution reaction (HER) has higher selectivity (87.6%). In contrast, NNU-55-Ni shows higher photocatalytic CO_2_-to-CO conversion selectivity (81.0%) and product yield (at least 85.3 μmol, 4265.0 μmol g^−1^,266.6 μmol g^−1^ h^−1^), and low HER selectivity (19%). The reason for such superior photocatalytic performance is that NNU-55-Ni can be in situ exfoliated into ultrathin two-dimensional (2D) nanosheets (NNU-55-Ni-NSs) during the photocatalytic CO_2_RR process to increase significantly accessible active surface area and the number of the catalytically active site. More importantly, according to the design idea of the above tandem catalytic reaction, we further combine the photocatalytic CO_2_RR with palladium-catalyzed carbonylation reaction of aryl iodide in series. Such a tandem reaction can realize the green, safe and efficient chain conversion of CO_2_ to CO to amide compounds (drug molecules and organic chemicals), which always has mature industrial separation technology and high-value application. It is worth noting that the low concentrations of CO generated from the photocatalytic CO_2_RR can be further converted into high-value DEET (N,N-diethyl-3-methylbenzamide, commercial pesticides) through the aminocarbonylation reaction, with the highest conversion of 85%. The present work represents an important case study that the low-value reductive product from photocatalytic CO_2_RR was reconverted into a high-value organic chemical through the cascade reaction. Obviously, the successful implementation of such a tandem reaction not only achieves the direct conversion of low-value CO_2_ to high-value drug-related molecules, but also opens up a new research avenue to upgrading the application value and subsequent separation of photocatalytic CO_2_RR products.

## Result

### Structure and characterization of NNU-55-M

**Fig. 2:**
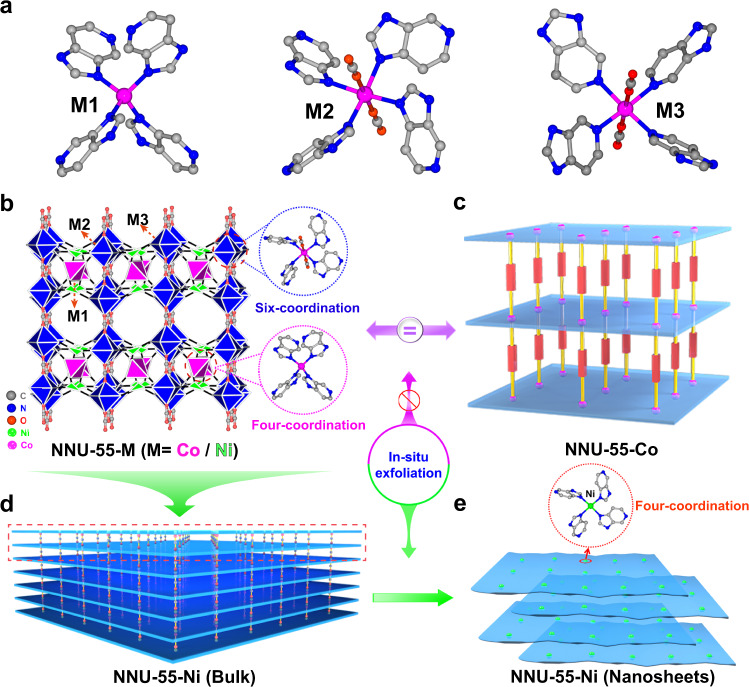
The crystal structure and in situ exfoliation process of NNU-55-M. **a** The coordination environments of three crystallographic independent M atoms (M1/M2/M3). **b** The three-dimensional framework and metal coordination environment of NNU-55-M (M = Co/Ni). **c** Schematic diagram of crystal structure to NNU-55-Co is corresponding to **b**. It shows that no such in situ exfoliation occurs to NNU-55-Co. **d** The schematic illustration of NNU-55-Ni (Bulk) shows the pillar-layer structure. The red dashed region is equated to **b**. **e** NNU-55-Ni (Bulk) experienced an in situ exfoliation process, NNU-55-Ni (Nanosheets) were formed.

The matched powder X-ray diffraction patterns of NNU-55-Ni and NNU-55-Co demonstrate that they are isostructural, and display high purity and good crystallinity (Fig. 2 and Supplementary Fig. 1). X‐ray single‐crystal diffraction analysis (Supplementary Note 1 and 2) indicates that NNU‐55-M crystallizes in the tetragonal system with a *P*4/*n* space group (Supplementary Tables 1 and 2). The asymmetric unit contains three 1/4 M^2+^ ions, one 5-AD ligand, two 1/4 CO_2_ molecules (Supplementary Figs. 2 and 3), and two free 1/4 acetate ions (Supplementary Fig. 4). Among the three crystallographic independent M atoms, M1 atom possesses a slightly distorted tetrahedral geometry and coordinates four imidazolyl N atoms from four different 5-AD ligands (Fig. 2a); the six-coordinated M2 and M3 atoms have a distorted octahedral coordination environment, and each M^2+^ ion is bonded to four imidazolyl N atoms (M2) or four pyridine N atoms (M3) from 5-AD ligands in the equatorial plane and two axial linear CO_2_ molecules (by M-O bonds) (Fig. 2a, b). The Co2 and Co3 atoms are connected by 5-AD ligands (N2 and N3) to form a monolayer structure (Supplementary Fig. 5). Then, two adjacent monolayers are further interconnected by Co1-N1 coordination bond forming 2D layers (Fig. 2c, d). The resulting 2D layers are further supported by the pillar CO_2_ molecules to form a 3D MOF (Fig. 2b and Supplementary Fig. 5). Of importance to note is that we calculated the binding energy of M–O bond between metal ion and CO_2_ pillar molecule in NNU-55-M by density functional theory (DFT) (Supplementary Note 3). The O binds with the planer M sites and the calculated binding energies are −0.45 eV and −0.28 eV for Co and Ni sites, respectively, suggesting a relatively stronger coordination ability for Co^2+^-CO_2_ coupling than Ni. Therefore, when the energy given by the specific external stimuli is larger than this M-O binding energy, the pillared CO_2_ molecules in NNU-55-Ni may tend to leave^37^ (Fig. 2e). In this case, NNU-55-M is likely to be split into a two-dimensional layered structure, and expose a larger active specific surface area and more active metal sites. In addition, thermogravimetric analysis indicated that NNU-55-M shows a high structural stability that can be maintained before heating to 350–400 °C (Supplementary Fig. 6a, b).

In view of the deep color for the crystals of NNU-55-Co/Ni, UV/Vis-NIR diffuse reflectance spectroscopy (UV/Vis-NIR DRS) was first characterized to evaluate their light-harvesting ability (Supplementary Fig. 7). As shown in Supplementary Fig. 7, NNU-55-Co/Ni displays a strong absorbance in the range of 400–800 nm, which means an obvious metal-ligand charge transfer (MLCT) effect existed in these MOFs, endowing them with good UV-visible-light absorption. Moreover, this fact can also be substantiated by the photocurrent response characterization, in which NNU-55-M can exhibit strong photoinduced electron transfer efficiency when the crystal surface was irradiated by visible-light (Supplementary Fig. 8). Meanwhile, the emission spectrum (Supplementary Fig. 9) and excited-state lifetimes (Supplementary Fig. 10a, b) of NNU-55-Co/Ni powder were tested, strong steady-state fluorescence and good photoluminescent (PL) lifetimes indicate the low recombination efficiency of photoinduced electron-hole pairs of NNU-55-Co/Ni (Supplementary Table 3). According to the Tauc plots, the band gaps (*E*_g_) of NNU-55-Ni and NNU-55-Co were calculated to be 1.71 and 1.72 eV, respectively, suggesting that these two MOFs feature semiconductor behavior^38^ (Supplementary Figs. 11 and 12). Besides, the HOMO-LUMO energy levels of NNU-55-M were estimated using ultraviolet photoelectron spectroscopy (UPS) technology. Then the ionization potential was obtained from the UPS of NNU-55-M (equivalent to valence band energy, HOMO), from which the HOMO levels of NNU-55-Ni and NNU-55-Co were determined to be −5.46 eV (Supplementary Fig. 13) and −5.29 eV (Supplementary Fig. 14, vs. vacuum level, *Ev*) by subtracting the excitation energy of 21.22 eV from the width of the He I UPS spectrum, while their corresponding LUMO levels were calculated as −1.10 V (NNU-55-Ni) and −1.28 V (NNU-55-Co)^39^. To ensure the reliability and accuracy of these energy level results, we further characterized Mott-Schottky measurements performed (Supplementary Note 4) at frequencies of 500, 1000 and 1500 Hz (Supplementary Figs. 15 and 16). The related results showed that the LUMO energy levels of NNU-55-Ni and NNU-55-Co were −1.10 V (Ni) and −1.28 V (Co), which were in good accordance with the UPS measurements. At the same time, the HOMO energy levels of NNU-55-Ni and NNU-55-Co were calculated as 0.61 V (Supplementary Fig. 17) and 0.44 V (Supplementary Fig. 18), respectively. It is obvious that these two MOFs have more negative LUMO levels, which make them to be potentially active catalysts favored for some reduction reactions.

### The photocatalytic CO_2_RR performance of NNU-55-M

**Fig. 3:**
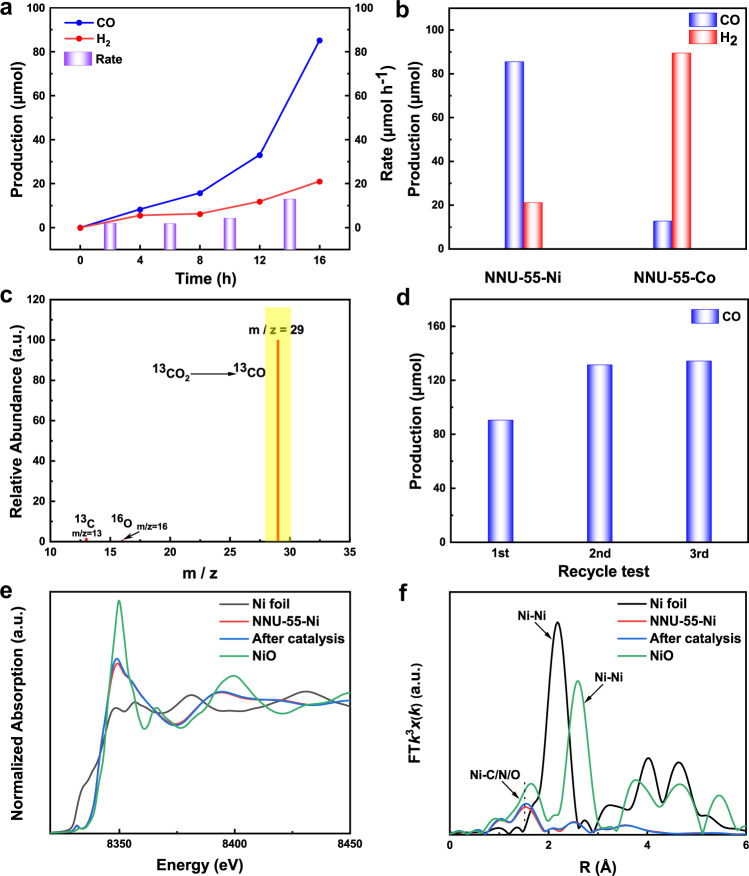
Photocatalytic performances of NNU-55-M. **a** A function of the irradiation time of CO and H_2_ production from CO_2_ photoreduction over NNU-55-Ni. The purple cylinder represents the reaction rate every 4 h. **b** The amount of CO and H_2_ produced with NNU-55-M. **c** Mass spectra (*m*/*z* = 29) analysis for reduction product ^13^CO (yellow shaded regions) under ^13^CO_2_ atmosphere. **d** The recycle experiments were conducted to NNU-55-Ni. **e** The experimental Ni K-edge XANES spectra of NNU-55-Ni samples before and after photocatalytic CO_2_RR as compared with calibrated standard Ni foil and NiO. **f** The *k*^3^-weighted FT-EXAFS spectra for NNU-55-Ni samples before and after photocatalytic CO_2_RR with Ni foil and NiO references.

Given that NNU-55-Co/Ni exhibited superior light absorption ability and photogenerated-charge transfer efficiency, and relatively more negative LUMO levels, thereby they were treated as crystalline catalysts for the CO_2_ photoreduction reaction. By optimizing the reaction conditions, we found that both MOF-based catalysts could realize a visible-light driven CO_2_-to-CO photoreduction reaction in a CO_2_-saturated CH_3_CN/H_2_O solution with triethanolamine (TEOA, SED) as electron donor and [Ru(bpy)_3_]Cl_2_·6H_2_O (bpy = 2′,2-bipyridine, PS) as photosensitizer. For NNU-55-Ni, with the illumination time was increased, a sustained increase of CO yield was observed until 16 h with the maximal generation of CO (85.3 μmol, 4265.0 μmol g^−1^, 266.6 μmol g^−1^ h^−1^) (Fig. 3a). This catalytic process has higher CO production in the reported MOF-based photocatalytic CO_2_RR systems (Supplementary Table 4). Moreover, the competitive HER reaction was effectively suppressed, and its maximum output was only 21.0 μmol (1050 μmol g^−1^, 65.6 μmol g^−1^ h^−1^), as shown in Fig. 3b. Therefore, NNU-55-Ni exhibited a very high selectivity (81.0%) for CO_2_-to-CO conversion. In order to investigate the stability of NNU-55-Ni before and after photocatalytic reaction, we explored the electronic structure and the local structures of Ni sites through X-ray absorption fine structure (XAFS) spectroscopy (Supplementary Fig. 19 and Supplementary Note 5). In the Ni k-edge X-ray absorption near-edge structure spectra of NNU-55-Ni, the oxidation state of Ni sites before and after reaction remained relatively constant (Fig. 3e). Moreover, compared to Ni foil and NiO reference sample, the very different of the peaks at 8331 and 8355 eV of NNU-55-Ni should be attributed to Ni^2+^ coordinating with N/C/O atoms in crystal structure leading to their different electronic structures^40^. Meanwhile, the Ni–Ni bond peak intensity in catalyst samples was much weakest at 2.2 and 2.6 Å than Ni foil and NiO reference sample, which was observed in the Fourier-transformed of the Ni K-edge extended XAFS spectra (Fig. 3f). It was obvious that no metallic Ni or NiO clusters/particles were formed for the catalyst before and after the reaction. Furthermore, these catalysts showed almost the same edge, also illustrating relatively good stability of the catalyst before and after photocatalytic CO_2_RR.

Under the identical photocatalytic CO_2_RR condition, whereas NNU-55-Co had relatively low CO output (12.6 μmol, 630 μmol g^−1^, 39.3 μmol g^−1^ h^−1^) but high H_2_ output (89.3 μmol, 4465.0 μmol g^−1^, 279.1 μmol g^−1^ h^−1^) (Supplementary Figs. 20 and 21). It is obvious that the photocatalytic CO_2_-to-CO selectivity of NNU-55-Co was very low (12.4%), and the competitive H_2_ evolution was very serious (Fig. 3b and Supplementary Figs. 22a, b and 23a, b). Meanwhile, the liquid phase product was detected by ion chromatography and ^1^H nuclear magnetic resonance (NMR), and the analytic results indicated that only a trace amount of formic acid was observed after photocatalytic reaction (Supplementary Fig. 24). Additionally, we also conducted a series of control analyses to confirm the photocatalytic CO_2_ reduction activity of NNU-55-Ni (Supplementary Table 5). The corresponding results showed that the photocatalytic CO_2_RR could not be carried out efficiently in the absence of catalyst, light irradiation, and the presence of only metal salts and ligands. These results proved that the above components involved in catalytic system are indispensable for the effective execution of photocatalytic CO_2_RR. At the same time, we further evaluated the photocatalytic CO_2_RR performance under illumination of monochromatic light with different incident wavelengths. The relevant results confirmed that the evolution trend of the apparent quantum efficiency (Supplementary Note 6) of the obtained photocatalytic products (CO and H_2_) matches the UV-vis absorption curve of [Ru(bpy)_3_]Cl_2_ (Supplementary Fig. 25). This research result indicated that the MLCT bands (Supplementary Fig. 26) of NNU-55-Ni (NS) have a small contribution to the performance of photocatalytic CO_2_RR (Supplementary Fig. 27a–c). To demonstrate the photocatalytic reduction product CO come from CO_2_ not derived from decomposition of organic substances, the identical photocatalytic reaction was conducted under nitrogen atmosphere. After the reaction, no carbon-based reductive products were detected. Furthermore, we performed an isotopic tracing experiment by using ^13^CO_2_ as the reaction atmosphere, and the peak at *m*/*z* = 29 detected by gas chromatography-mass spectrometer (GC-MS) could be attributed to ^13^CO (Fig. 3c), which confirmed clearly that the CO product was really originated from the gaseous CO_2_ reactant. Subsequently, three consecutive recycling experiments of NNU-55-Ni were performed under the same reaction condition to evaluate its photocatalytic durability (Fig. 3d). The corresponding results demonstrated that NNU-55-Ni could keep its original photocatalytic activity at least 48 h. Moreover, it was worth noting that with the number of cycles increased, the photocatalytic activity of the catalyst was improved dramatically^41^ (The total output can reach up to 342.0 μmol after three rounds of reaction) (Supplementary Fig. 28). This phenomenon made the total amount of CO generate increasingly by extending the reaction time, which means that the CO generation rate per unit time is getting much faster as shown in Fig. 3a. Interestingly, we found that the original reaction solution containing NNU-55-Ni solid became a suspension and showed a conspicuous Tyndall effect, indicating that the size of the catalyst was significantly decreased during the catalysis turnovers^42^. From the structural analysis of NNU-55-Ni, most of the M^2+^-CO_2_ coordination bonds (with low binding energy) between two-dimensional layers may break under the stimuli of continuous light energy input and violent stirring of the catalytic reaction, and then the catalyst evolved into a two-dimensional nanosheet structure. We speculated that the higher accessible catalytic active specific surface area and more metal catalytic active sites (the original saturated six-coordinated Ni2 and Ni3 atoms changed into unsaturated four-coordination) were generated during the stripping process of bulk NNU-55-Ni to nanosheets, thus enhancing the photocatalytic performance efficiently (Supplementary Figs. 28 and 29).

### Characterization and photophysical properties study of nanosheets

**Fig. 4:**
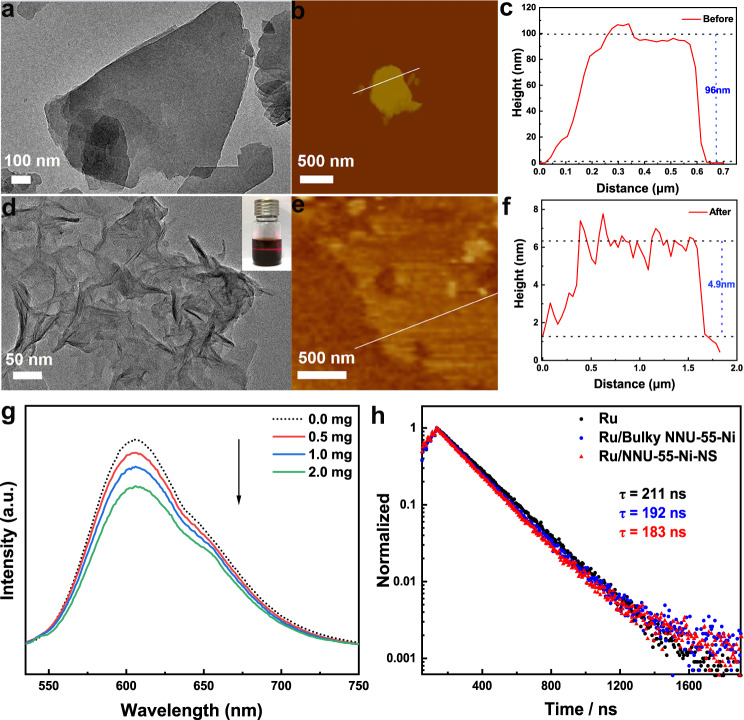
Characterization and optical properties of NNU-55-Ni-NS. **a**–**c** TEM and AFM images of the bulky NNU-55-Ni. The height profile along the white line represents the thickness of the bulky NNU-55-Ni. **d**–**f** After the bulky NNU-55-Ni was in situ exfoliated into NNU-55-Ni-NS, leading to a change in TEM, AFM, and height profile. **g** The steady-state fluorescent spectra of [Ru(bpy)_3_]Cl_2_ upon the addition of increasing amounts of NNU-55-Ni-NS. **h** Time‐resolved fluorescence decay spectra of [Ru(bpy)_3_]Cl_2_ in the presence of different catalysts.

To prove our speculation, scanning electron microscopy (SEM) and transmission electron microscopy (TEM) were employed to observe the morphology and size changes of the NNU-55-Ni catalysts before and after the photocatalytic process (Supplementary Figs. 30 and 31). Obviously, TEM and SEM characterizations showed that after the photocatalytic reaction, bulk NNU-55-Ni was exfoliated into ultrathin 2D nanosheets (NS = nanosheet) (Fig. 4a, d). And we have also effectively measured the size change of the catalyst before and after the photocatalytic reaction through atomic force microscopy in order to evaluate the degree of exfoliation. It showed that the thickness of the catalyst structure was sharply reduced from 96 nm (*ca.* 100 layers) (Fig. 4b) to 4.9 nm (~5 layers) (Fig. 4e) after undergoing the reaction, which closed to a 20-fold reduction, clearly proving an effective exfoliation process from 3D structure to 2D ultrathin NS structure (i.e., NNU-55-Ni-NS) (Fig. 4c, f). After peeling off process, the NNU-55-Ni-NS not only has a larger accessible specific surface area and more catalytically active metal sites, but also has excellent electron transfer and mass transport properties, in accordance to other 2D NSs demonstrated in previous important works^43,44^. To further verify the excellent electron transport performance of NNU-55-Ni-NS and study the quenching mechanism of the photocatalytic process, PL quenching experiments (Supplementary Note 7) and fluorescence excited-state lifetimes were tested. The PL spectra were performed under the condition of photocatalytic reaction without TEOA, the PL intensity of [Ru(bpy)_3_]Cl_2_ (Ex = 400 nm) followed linear Stern-Volmer behavior and decreased significantly as the increased catalyst concentration (Fig. 4g, Supplementary Fig. 32), which means that the photogenerated electrons could be transferred from [Ru(bpy)_3_]Cl_2_ to NNU-55-Ni-NS effectively. However, in the photocatalytic system without catalyst, the fluorescence intensity of PS cannot be quenched (Supplementary Fig. 33). Moreover, this charge transfer mode was also confirmed by the UV-Vis absorption spectrum experiment of [Ru(bpy)_3_]Cl_2_ (Supplementary Figs. 34 and 35). These experiment results indicated that in the process of photocatalytic CO_2_RR, the photogenerated electrons are mainly transferred from the light-excited [Ru(bpy)_3_]Cl_2_ to NNU-55-Ni-NS forming PS^+^, rather than get electrons firstly from TEOA to the excited PS*. Therefore, the photocatalytic process in our reaction system experienced an oxidative quenching mechanism. In addition, [Ru(bpy)_3_]Cl_2_ presented different photoluminescence lifetimes in reaction solutions involving different sizes of the catalysts. As shown in Fig. 4h, in the pure [Ru(bpy)_3_]^2+^ solution, the single excited emission followed the exponential first-order decay function, with a lifetime of 211 ns. For [Ru(bpy)_3_]^2+^ solution containing NNU-55-Ni-NS, however, we could clearly observe a decay of triplet metal-to-ligand charge transfer (^3^MLCT) excited state. Obviously, by fitting the fluorescence decay curves, the photoluminescence lifetime of photosensitizer decreased from 211 ns to 183 ns after adding catalyst (the difference value is *ca*. 28 ns) (Supplementary Table 6), indicating that the photoexcited electrons from photosensitizer were quickly transferred to the catalyst. Moreover, this charge transfer effect was more obvious and effective between photosensitizer and NNU-55-Ni-NS than between photosensitizer and bulky catalyst (Fig. 4h and Supplementary Table 6). In conclusion, the results of time-resolved PL decay spectroscopy experiment and PL quenching experiment could also prove that a significant charge transfer effect occurred between photosensitizer and NNU-55-Ni-NS catalyst and inhibited the recombination of photoexcited electrons and holes in the reaction system. Accordingly, the formation of NNU-55-Ni-NS with larger specific surface area and more exposed active metal sites is the main reason for the effective improvement of CO_2_ photoreduction activity^45^. But for NNU-55-Co with the relatively higher coordination binding energy, the exfoliation process did not occur under the identical photocatalytic reaction condition. Therefore, it finally displayed the poor photocatalytic CO_2_RR Performance due to the low specific surface area and less active metal sites. In addition, the stability of chemical composition and metal oxidation state of catalysts was confirmed by the nearly unchanged XRD (Supplementary Figs. 36 and 37), XPS (Supplementary Fig. 38a, b) and FTIR (Supplementary Fig. 39a, b) spectra before and after the photocatalytic reaction.

### DFT theoretical calculation

**Fig. 5:**
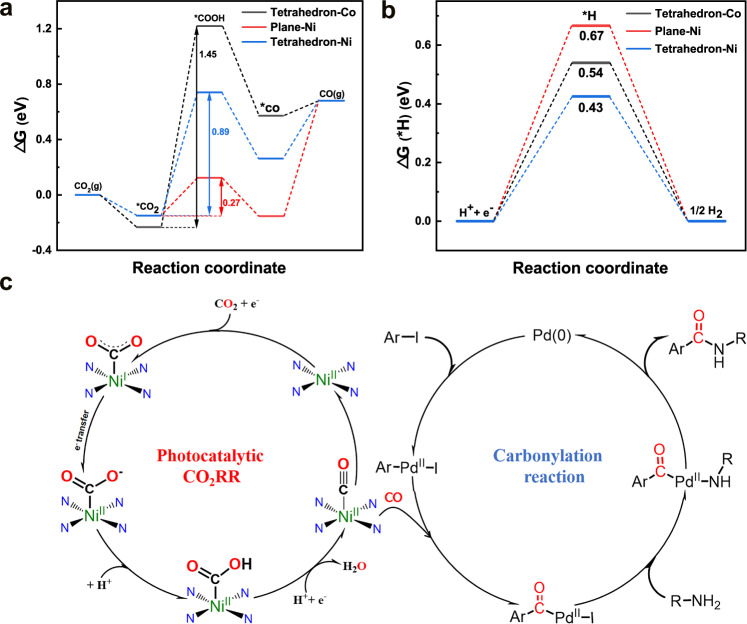
DFT calculations were performed to understand the effects of different configurations on the photoreduction of CO_2_. **a** Free energy diagram of CO_2_RR toward the production of CO. **b** Free energy profile of HER. **c** The possible reaction mechanism of the tandem reaction.

At the same time, DFT calculations were employed to further study the catalytic difference on CO_2_ photoreduction performance of NNU-55-Co and NNU-55-Ni. We have studied the free energy pathway for reducing CO_2_ to CO through free energy diagrams on the catalytically active site of NNU-55-M, and the HER occurs as a competitive process (Supplementary Fig. 40). Four consecutive reaction steps of CO_2_RR on the metal catalytically active site have been considered, and the formation of *COOH is the rate-limiting step among them (Supplementary Figs. 41a, b). It is difficult to break the Co-O coordination bond due to having strong binding energy (−0.45 eV) between CO_2_ molecular and Co^2+^ ion, so the saturated hexa-coordination sites cannot serve as catalytically active sites for CO_2_ reduction process or HER. Gibbs free energy calculations disclosed that the COOH* intermediate formation step of the catalytically tetrahedral active Co^2+^sites had a free energy activation barrier (Δ*G*) of 1.453 eV, which was much larger than that of photocatalytic HER. So the NNU-55-Co catalyst is more active for HER rather than CO_2_RR, which agrees with those obtained experimentally. For the isomorphic NNU-55-Ni, because it undergoes an in situ exfoliation process during photocatalytic CO_2_RR to destroy Ni-O coordination bond, the resultant NNU-55-Ni-NS exposes more planar four-coordinated active Ni^2+^ sites. On this site, the Δ*G* for *COOH intermediate formation is only 0.272 eV and the Δ*G* for HER is 0.667 eV (Fig. 5a, b). For the other type of Ni^2+^ active site with tetrahedral configuration, the Δ*G* of *COOH intermediate and HER are 0.890 eV and 0.425 eV, respectively. Therefore, the planer Ni sites are identified as the active sites for CO_2_RR based on thermodynamics analysis and the NNU-55-Ni present superior catalytic performance for photocatalytic reduction of CO_2_ to CO as observed experimentally (Fig. 5c).

### An idea for designing tandem reaction system

**Fig. 6:**
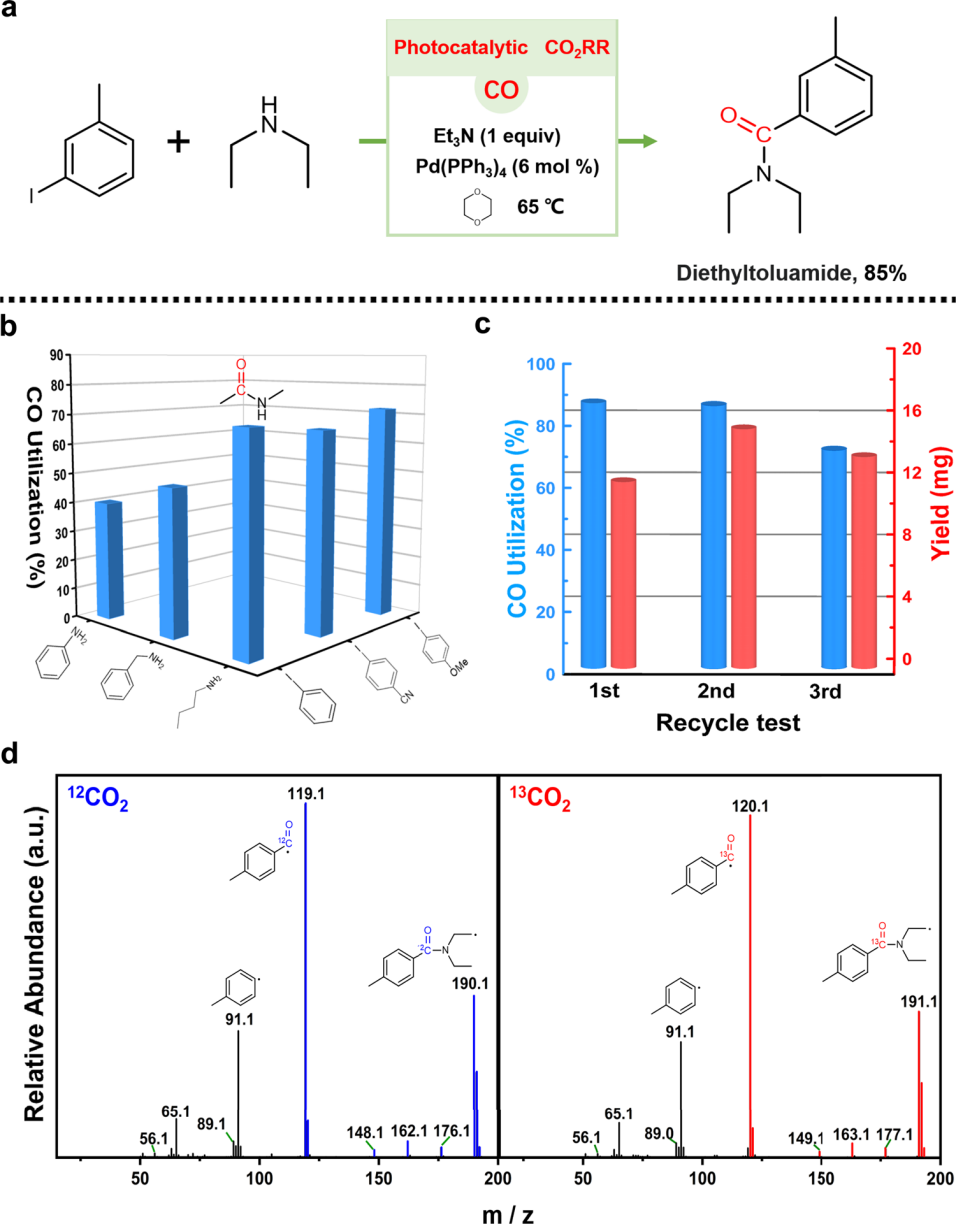
DEET production by tandem catalytic reaction and substrates screening were performed to determine the optimum conditions. **a** Schematic illustration of the synthesis procedure of DEET via tandem reaction and actual CO utilization. **b** Select optimization conditions of the carbonylation reaction. **c** The recycle experiments of tandem reaction were conducted. **d** GC-MS shows the different mass charge ratios (*m*/*z*) when ^12^CO_2_ (blue) and ^13^CO_2_ (red) as the source of CO by the tandem reaction to synthesize DEET. Further details about high-resolution mass spectrometry are provided in Supplementary Fig. 47.

In the currently reported works for photocatalytic CO_2_RR, the final reductive products are rarely further transformed or utilized. Although NNU-55-Ni in this work can exhibit high photocatalytic selectivity, activity and durability for CO_2_-to-CO conversion, we hope that the reductive product CO with lower use value can be further converted into fine organic chemicals with high use value and mature separation technology (e.g., drug molecules, chemical raw materials, etc.) through tandem catalytic reactions^46^. It may be a feasible strategy for the future industrial application of CO_2_ photoreduction reaction technology^47^. More significantly, such a tandem conversion reaction pathway is also equivalent to realize the direct conversion from cheap CO_2_ to value-added carbon-based products. At the same time, the corresponding tandem catalytic reaction can also elude the potential dangers caused by the direct use of large doses of toxic CO reactants^48^. With this idea in mind, we chose to effectively connect the NNU-55-Ni-catalyzed CO_2_ photoreduction reaction with the aminocarbonylation by coupling organic reaction in series. The aminocarbonylation process generally follows a pathway starting with oxidative addition of palladium (0) center to Ar-X and insertion of CO to the organopalladium complex to form the corresponding carbonyl palladium complex, and then following by transmetallation and reductive elimination to afford the amide product^49,50^ (Fig. 5c). Therefore, by this cascade reaction, not only the low-value and low-concentration CO can be reconverted into important amide drug molecules or commercial products, but these resulting products already have matured industrial separation technology for practical application. Moreover, NNU-55-Ni-catalyzed CO_2_ photoreduction reaction can provide a sustainable, safe and green CO generation pathway for the aminocarbonylation. Thus, this kind of tandem reaction can effectively realize a strategy of killing two birds with one stone (Fig. 6).

### The synthesis of DEET and ^13^C isotope-labeled experiment by using the tandem reaction

**Fig. 7:**
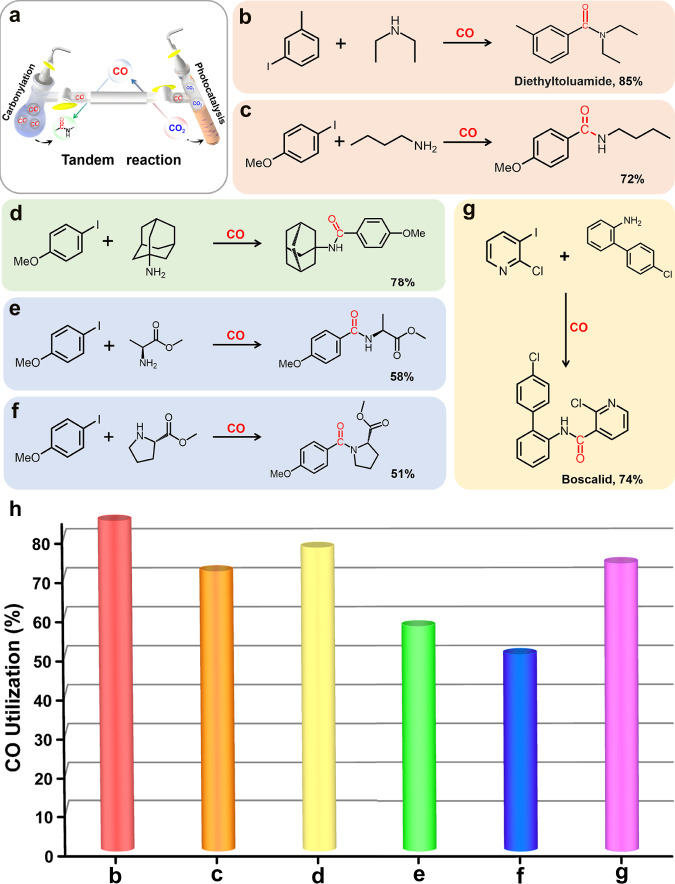
Application of the photocatalytic CO_2_RR to aminocarbonylative coupling. **a** Schematic illustration of the experimental apparatus and device structure. **b**–**g** Synthesis of pharmaceuticals molecules, fine chemicals, and other biologically active molecules. **h** The tandem reaction showed the different CO utilization for different reaction substrates.

Connecting two devices of different catalytic reaction regimes in series effectively is extremely important to successfully implementing of the tandem reaction. As shown in Fig. 7, the tandem reaction apparatus was built by connecting two independent chambers with good airtightness (Fig. 7a and Supplementary Fig. 42). The right chamber for photocatalytic CO_2_RR is utilized to produce continuous CO in a safe and green way, and then CO as a reactant can freely diffuse into the left chamber to undergo aminocarbonylation reactions and finally facilitate the synthesis of amide products (Supplementary Fig. 43). Through this tandem reaction device, we first tried to effectively connect the photocatalytic CO_2_RR (NNU-55-Ni as a catalyst) and the aminocarbonylation reaction of aryl iodide in series. Among the tandem reaction, the photocatalytic CO_2_RR was performed on the above optimal reaction conditions to obtain the highest CO yield (85.3 μmol), while the aminocarbonylation reaction of aryl iodide was carried out in 1,4-dioxane with Et_3_N as the organic base and tetrakis (triphenylphosphine) palladium-(0) catalyst. The reductive product CO from CO_2_ photoreduction reaction could be reconverted into the diethyltoluamide (DEET) (Supplementary Note 8 and 9), which is one of commercial pesticides to prevent some diseases of transmitted by mosquito vector, completing the chain conversion of CO_2_ to CO to DEET (Figs. 6a and 7b). In other words, such a reaction pathway also realized the direct conversion of low-value CO_2_ to high-value DEET. In addition, hydrogen as the byproduct of photocatalytic CO_2_RR almost had no effect on the palladium-catalyzed aminocarbonylation. After 16 h of tandem reaction, the conversion of CO to DEET reached as high as 85%. It is worth noting that the final DEET was effectively separated and the isolated product mass was about 15.2 mg (Supplementary Table 7). To further verify the sustainability of the tandem reaction, we tested three consecutive cycles of the photocatalytic CO_2_RR and the results showed that the tandem aminocarbonylation reaction still maintained a high CO conversion as demonstrated in Fig. 6c. Meanwhile, we attempted higher-scale experiments to obtain a higher yield of the target product. As shown in Supplementary Fig. 44, as the number of rounds of the photocatalytic cycle increased, the carbonylation reaction proceeded and the target amide compound (DEET) was accumulated continually (Supplementary Fig. 45), as demonstrated the deepen color of the reaction solution (Supplementary Fig. 44a). And the final purely organic product (DEET) yield could reach 84.4 mg (Supplementary Fig. 44b). Consequently, the tandem reaction successfully verified our initial purpose that reconverting the photocatalytic CO_2_RR product (CO) with low-value and low-concentration into value-added and easily isolated fine organic chemical. Furthermore, we also successfully synthesized isotopically labeled organic products through an isotope (^13^CO_2_) tracing experiment. During the tandem catalytic reaction, the injected isotope ^13^CO_2_ atmosphere was reduced to ^13^CO through photocatalytic CO_2_RR, and then ^13^CO as reactant undergone the tandem aminocarbonylation reaction to form isotope-labeled amide compounds (Supplementary Fig. 46). After that, the successful synthesis of ^13^C isotope-labeled DEET was confirmed by GC-MS (Fig. 6d) and high-resolution mass spectrometry characterization (Supplementary Fig. 47a, b). Therefore, this cascade reaction represents an effective method to use ^13^CO_2_ as a source of isotopically labeled amide molecules, suggesting a great promise in the research of carbon isotope labeling chemistry.

### The substrate scope extension experiments in the tandem reaction

In order to evaluate the effectiveness of tandem reactions, we also explored the catalytic activity of other substrates with different functional substitutes under the same reaction conditions (Fig. 6b and Supplementary Table 7). When aniline and benzylamine were used as reactants, the CO conversions for amide formation were low (40 and 49%) (Supplementary Notes 10 and 11). The conversion of CO was then significantly improved by applying *n*-butylamine as the coupling partner (70%) (Supplementary Note 12). The reason of the relatively higher yield could be attributed to the relatively strong nucleophilicity of alkyl primary amine than its aromatic analog. Further examination demonstrated that aryl iodides with electron-donating group (-OMe) and electron-withdrawing group (-CN) as substitutes underwent the cascade reaction conditions smoothly^51^, providing the amides in 72% (Fig. 7c) and 67% yields (Supplementary Notes 13 and 14).

We then carried out more of the tandem reactions by using 4-iodoanisole and different amines as the coupling partners. A conversion of 78% on the basis of CO-to-d (Fig. 7d) was detected when amantadine was employed as the amino source (Supplementary Note 15). Amines with steric hindrance^52^, such as L-Alanyl methyl ester and D-Proline methyl ester, were viable partners to this tandem photocatalytic reductive-aminocarbonylation system (Supplementary Notes 16 and 17), with CO conversions of 58 and 51% observed (Figs. 7e, f). The cascade reaction strategy was also applied to synthesize Boscalid (Fig. 7g), a fungicide active against a broad range of fungal pathogens, with good CO conversion (74%) detected, though a hetero aryl iodide^53^—iodopyridine derivative and a relative more structural complicated aniline were required (Supplementary Note 18). These successful results have fully revealed the application prospect and universality of the direct transferring CO_2_ to important organic chemicals by connecting the photocatalytic CO_2_RR with palladium-catalyzed aminocarbonylation reaction (Fig. 7h and Supplementary Table 7).

### The reaction type extension in the tandem reaction

**Fig. 8:**
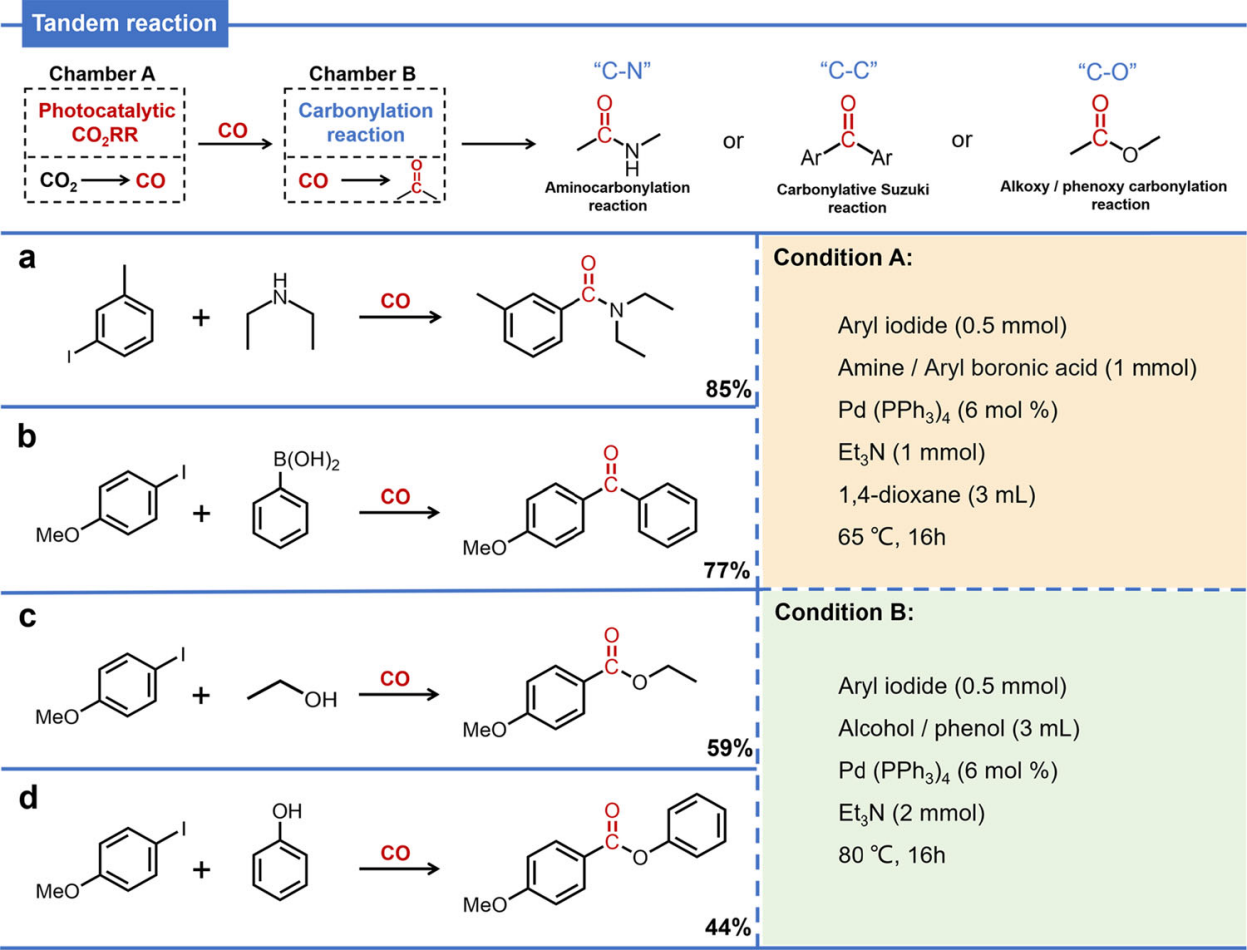
Expanding different carbonylation reactions to our tandem reaction. **a** Aminocarbonylation reaction using tandem catalysis system. **b** Carbonylative Suzuki coupling reactions using tandem catalysis system. **c**, **d** Phenoxycarbonylation/alkoxycarbonylation reaction using tandem catalysis system.

To further confirm the synthetic utilities of this tandem catalytic system, we also tried to combined the photocatalytic CO_2_RR with other carbonylation reactions, such as carbonylative Suzuki coupling, phenoxycarbonylation and alkoxycarbonylation. When the carbonylative Suzuki coupling was applied in the follow-up chamber under the standard conditions (Condition A) with phenylboronic acid and 4-iodoanisole as the reaction partners, the corresponding biarylketone was detected, and the conversion of CO generated by photocatalytic CO_2_RR was 77% (Fig. 8b and Supplementary Note 19). However, the phenoxycarbonylation / alkoxycarbonylation reaction was found sluggish under the same conditions (Condition A). We suppose that the weaker nucleophilicity of oxygen should be responsible for that. Therefore, a modification of the reaction conditions for the carbonylation reaction chamber has been applied, and the tandem catalytic reaction are implemented (Condition B). For the alkoxycarbonylation reaction, when ethanol is used as the nucleophile, ester compounds could be formed through sequential transformation and the CO conversion could reach 59% (Fig. 8c and Supplementary Note 20). Furthermore, conducting the reaction by replacing the nucleophile with phenol, led to the success of the phenoxycarbonylation with the CO conversion at 44% (Fig. 8d and Supplementary Note 21). To summarize, we successfully cascade photocatalytic CO_2_RR and different types of carbonylation reactions to completing the chain conversion of CO_2_ to CO to amide (Fig. 8a)/ketone (Fig. 8b)/ester (Figs. 8c, d) compounds involving the formation of C–N/C–C/C–O bond, which further expands and highlight the practical application of tandem reactions.

## Discussion

In summary, we have designed and synthesized a stable MOF-based catalyst system for photocatalytic CO_2_RR, in which NNU-55-Ni exhibits high activity, selectivity and durability for CO_2_ to CO photochemical conversion. Since NNU-55-Ni was exfoliated in situ to form ultrathin 2D nanosheets during the photocatalytic reaction process, resulting in the larger active specific surface area, more active metal sites and much faster electron transfer rate, so the photocatalytic performance of CO_2_RR was significantly improved. More importantly, we successfully achieved the re-conversion from the photocatalytic low-value product CO to value-added and easily separated fine organic chemicals through connecting photocatalytic CO_2_RR and the aminocarbonylation reaction in series. Such a tandem reaction tactic showed very high conversion and the application potential in the synthesis fine chemicals. In the meantime, we also achieved ^13^C isotopic labeling of bioactive molecules in an environmentally friendly manner. This work not only realized the value enhancement and separation application of photocatalytic low-value reduction product, but also provided a feasible and promising pathway for the subsequent application of the photocatalytic CO_2_RR.

## Methods

### Synthesis of NNU-55-Co

A solid mixture of 5-Azabenzimidazole (5-AD) (0.017 mmol), Co (CH_3_COO)_2_·6H_2_O (0.14 mmol) was suspended in DMF-H_2_O (3:1, 4 ml) solution in a 10 ml vial. Then the pH value of the solution was adjusted by addition of nitric acid solution (50 μl). The sonication steps take ~30 min. After the solution became clear, the vial was heated at 120 °C for 7 days and then slowly cooled to room temperature. Purple crystals of NNU-55-Co was obtained, and the yield was 58% (base on 5-AD). The CCDC number of NNU-55-Co is 2081042.

### Synthesis of NNU-55-Ni

NNU-55-Ni was prepared by a similar method except that Ni (CH_3_COO)_2_·6H_2_O was used instead of Co (CH_3_COO)_2_·6H_2_O. The yield of the NNU-55-Ni dark blue powder was 48% (base on 5-AD).

### Calculational methods

All calculational methods are provided in Supplementary Note 3.

### General procedure for photocatalytic CO_2_ reduction experiments

The photocatalytic CO_2_RR was carried out in a 25 ml quartz reactor at a temperature of 25 °C maintained by using a thermostatic water bath with as-prepared crystal. Catalyst (20 mg) and [Ru(bpy)_3_]Cl_2_·6H_2_O (0.013 mmol) were added into the mixed solution which contained acetonitrile (10 ml) and deionized water (100 μl). Then TEOA (23 mmol) was added into the reaction mixture solution. The reaction system was saturated by CO_2_ for 30 min with a cap at 1 atm CO_2_ partial pressure and then irradiated using a Xe lamp (*λ* ≥ 420 nm, 300 W). The typical test is 16 h. The gas products in the headspace of the reactor were measured using GC with an FID detector to analyze CO/CH_4_/CO_2_ and a TCD detector to analyze O_2_ and H_2_.

### Photophysical experiments

PL quenching experiments and photoluminescence lifetime experiments are provided in Supplementary Note 7.

### Tandem reaction experiments

For a better comparison, all organic measurements were carried out under equivalent experimental conditions. Achieving high gas density in the reaction volume, the relatively small volume of the chamber reactor (Schlenk tube) was selected (A chamber, 25 ml and B chamber, 10 ml). A chamber was used for photocatalytic CO_2_RR according to the protocol above. The reaction conditions in chamber B were changed according to different organic catalysis. Condition A: B chamber was loaded with aryl iodide (0.5 mmol), Pd(PPh_3_)_4_ (6 mol%), Et_3_N (139 μl, 1 mmol), and the corresponding amine or aryl boronic acid (1 mmol). Subsequently, anhydrous 1,4-dioxane (3 ml) was added to the reactor. Condition B: B chamber was loaded with aryl iodide (0.5 mmol), Pd(PPh_3_)_4_ (6 mol%), Et_3_N (139 μl, 1 mmol). Due to Phenoxy/alkoxy compounds act as both solvent and reactant, and the corresponding alcohol (3 ml) or phenol (3.2 g, 34.1 mmol) was added to the reactor. The two chambers were chained together by a short linker, all joints were in a closed state. Subsequently, only open the joint of B, and the B chamber was then flash frozen at 77 K (liquid N_2_ bath) and degassed by three freeze-pump-thaw cycles, and evacuated to an internal pressure of ~100 mTorr. Then warm to room temperature, A chamber was placed under photocatalytic conditions, the B chamber was placed in an oil bath. After photocatalytic reacting for a set amount of time, A joint was slowly opened and B chamber was stirred at 65 °C for 16 h in the oil bath. The reaction product was detected and quantified by mass spectrometry (5977B Agilent Technologies) and NMR analysis after purification by column chromatography on silica gel.

## Supplementary information

Supplementary Information

## Data Availability

The data that support the findings of this study are available within the paper and its Supplementary Information files or are available from the corresponding authors upon reasonable request. Source data are provided with this paper.

## Source data

Source data

## References

[1] Sudarsanam P (2018). Functionalised heterogeneous catalysts for sustainable biomass valorisation. Chem. Soc. Rev..

[2] Wang S (2019). Porous hypercrosslinked polymer-TiO_2_-graphene composite photocatalysts for visible-light-driven CO_2_ conversion. Nat. Commun..

[3] Kou M (2021). Photocatalytic CO_2_ conversion over single-atom MoN_2_ sites of covalent organic framework. Appl. Catal. B..

[4] Habisreutinger SN, Schmidt-Mende L, Stolarczyk JK (2013). Photocatalytic reduction of CO_2_ on TiO_2_ and other semiconductors. Angew. Chem. Int. Ed..

[5] Zhang T, Lin W (2014). Metal-organic frameworks for artificial photosynthesis and photocatalysis. Chem. Soc. Rev..

[6] Kong T, Jiang Y, Xiong Y (2020). Photocatalytic CO_2_ conversion: what can we learn from conventional COx hydrogenation?. Chem. Soc. Rev..

[7] Wang S, Guan BY, Lu Y, Lou XWD (2017). Formation of hierarchical In2S3–CdIn2S4 heterostructured nanotubes for efficient and stable visible light CO_2_ reduction. J. Am. Chem. Soc..

[8] Kim D, Sakimoto KK, Hong D, Yang P (2015). Artificial photosynthesis for sustainable fuel and chemical production. Angew. Chem. Int. Ed..

[9] Li X (2019). Selective visible-light-driven photocatalytic CO_2_ reduction to CH_4_ mediated by atomically thin CuIn_5_S_8_ layers. Nat. Energy.

[10] Jiang Z (2020). Filling metal-organic framework mesopores with TiO_2_ for CO_2_ photoreduction. Nature.

[11] Vu N-N, Kaliaguine S, Do T-O (2019). Critical aspects and recent advances in structural engineering of photocatalysts for sunlight-driven photocatalytic reduction of CO_2_ into fuels. Adv. Funct. Mater..

[12] Guo Q (2019). Efficient and selective CO_2_ reduction integrated with organic synthesis by solar energy. Chem.

[13] Wu YA (2019). Facet-dependent active sites of a single Cu_2_O particle photocatalyst for CO_2_ reduction to methanol. Nat. Energy.

[14] Yan Z-H (2018). Photo-generated dinuclear {Eu(II)}_2_ active sites for selective CO_2_ reduction in a photosensitizing metal-organic framework. Nat. Commun..

[15] Lan G (2018). Photosensitizing metal-organic layers for efficient sunlight-driven carbon dioxide reduction. J. Am. Chem. Soc..

[16] Guo Z (2019). Selectivity control of CO versus HCOO^−^ production in the visible light-driven catalytic reduction of CO_2_ with two cooperative metal sites. Nat. Catal..

[17] Wang S, Yao W, Lin J, Ding Z, Wang X (2014). Cobalt imidazolate metal-organic frameworks photosplit CO_2_ under mild reaction conditions. Angew. Chem. Int. Ed..

[18] Takeda H (2018). Highly efficient and robust photocatalytic systems for CO_2_ reduction consisting of a Cu(I) photosensitizer and Mn(I) catalysts. J. Am. Chem. Soc..

[19] Lu M (2019). Rational design of crystalline covalent organic frameworks for efficient CO_2_ photoreduction with H_2_O. Angew. Chem. Int. Ed..

[20] Pan Y-X (2017). Photocatalytic CO_2_ reduction by carbon-coated indium-oxide nanobelts. J. Am. Chem. Soc..

[21] Liang L (2020). Efficient infrared light induced CO_2_ reduction with nearly 100% CO selectivity enabled by metallic CoN porous atomic layers. Nano. Energy.

[22] Franke R, Selent D, Börner A (2012). Applied hydroformylation. Chem. Rev..

[23] Xiang Y, Kruse N (2016). Tuning the catalytic CO hydrogenation to straight- and long-chain aldehydes/alcohols and olefins/paraffins. Nat. Commun..

[24] Ai H-J, Zhao F, Geng H-Q, Wu X-F (2021). Palladium-catalyzed thiocarbonylation of alkenes toward linear thioesters. ACS Catal..

[25] Peng J-B, Geng H-Q, Wu X-F (2019). The chemistry of CO: carbonylation. Chem.

[26] Lian Z, Nielsen DU, Lindhardt AT, Daasbjerg K, Skrydstrup T (2016). Cooperative redox activation for carbon dioxide conversion. Nat. Commun..

[27] Ibrahim MB, Suleiman R, Fettouhi M, El Ali B (2016). A palladium–bisoxazoline supported catalyst for selective synthesis of aryl esters and aryl amides via carbonylative coupling reactions. RSC Adv..

[28] Chen M (2020). Palladium-catalyzed enantioselective Heck carbonylation with a monodentate phosphoramidite ligand: asymmetric synthesis of (+)-Physostigmine, (+)-Physovenine, and (+)-Folicanthine. Angew. Chem. Int. Ed..

[29] Friis SD, Taaning RH, Lindhardt AT, Skrydstrup T (2011). Silacarboxylic acids as efficient carbon monoxide releasing molecules: Synthesis and application in palladium-catalyzed carbonylation reactions. J. Am. Chem. Soc..

[30] Friis SD, Lindhardt AT, Skrydstrup T (2016). The development and application of two-chamber reactors and carbon monoxide precursors for safe carbonylation reactions. Acc. Chem. Res..

[31] Lang X-D, You F, He X, Yu Y-C, He L-N (2019). Rhodium(i)-catalyzed Pauson–Khand-type reaction using formic acid as a CO surrogate: an alternative approach for indirect CO_2_ utilization. Green. Chem..

[32] Morimoto T, Kakiuchi K (2004). Evolution of carbonylation catalysis: no need for carbon monoxide. Angew. Chem. Int. Ed..

[33] Nielsen DU, Hu X-M, Daasbjerg K, Skrydstrup T (2018). Chemically and electrochemically catalysed conversion of CO_2_ to CO with follow-up utilization to value-added chemicals. Nat. Catal..

[34] Hermange P (2011). Ex situ generation of stoichiometric and substoichiometric ^12^CO and ^13^CO and its efficient incorporation in palladium catalyzed aminocarbonylations. J. Am. Chem. Soc..

[35] Ren X (2017). Rhodium-complex-catalyzed hydroformylation of olefins with CO_2_ and hydrosilane. Angew. Chem. Int. Ed..

[36] Gotico P (2018). Visible-light-driven reduction of CO_2_ to CO and its subsequent valorization in carbonylation chemistry and ^13^C isotope labeling. ChemPhotoChem.

[37] Huang J (2018). Electrochemical exfoliation of pillared-layer metal-organic framework to boost the oxygen evolution reaction. Angew. Chem. Int. Ed..

[38] Wang X-K (2019). Monometallic catalytic models hosted in stable metal-organic frameworks for tunable CO_2_ photoreduction. ACS Catal..

[39] Liu J (2015). Metal-free efficient photocatalyst for stable visible water splitting via a two-electron pathway. Science.

[40] Jiang J (2021). Van der waals heterostructures by single cobalt sites-anchored graphene and g-C3N4 nanosheets for photocatalytic syngas production with tunable CO/H2 ratio. Appl. Catal., B..

[41] Zheng H-L (2020). Photochemical in-situ exfoliation of metal-organic frameworks for enhanced visible-light-driven CO_2_ reduction. Angew. Chem. Int. Ed..

[42] Peng Y (2014). Metal-organic framework nanosheets as building blocks for molecular sieving membranes. Science.

[43] Cao L (2016). Self-supporting metal-organic layers as single-site solid catalysts. Angew. Chem. Int. Ed..

[44] Zhao M (2015). Ultrathin 2D metal-organic framework nanosheets. Adv. Mater..

[45] Ding Y (2017). Controlled intercalation and chemical exfoliation of layered metal-organic frameworks using a chemically labile intercalating agent. J. Am. Chem. Soc..

[46] He X, Cao Y, Lang X-D, Wang N, He L-N (2018). Integrative photoreduction of CO_2_ with subsequent carbonylation: photocatalysis for reductive functionalization of CO_2_. ChemSusChem.

[47] Lescot C (2014). Efficient fluoride-catalyzed conversion of CO_2_ to CO at room temperature. J. Am. Chem. Soc..

[48] Nielsen DU, Lescot C, Gøgsig TM, Lindhardt AT, Skrydstrup T (2013). Pd-catalyzed carbonylative α-Arylation of aryl bromides: scope and mechanistic studies. Chem. Eur. J..

[49] Minatti A, Muñiz K (2007). Intramolecular aminopalladation of alkenes as a key step to pyrrolidines and related heterocycles. Chem. Soc. Rev..

[50] D’Souza DM, Müller TJJ (2007). Multi-component syntheses of heterocycles by transition-metal catalysis. Chem. Soc. Rev..

[51] Nordeman P, Odell LR, Larhed M (2012). Aminocarbonylations employing Mo(CO)_6_ and a bridged two-vial system: allowing the use of Nitro group substituted aryl iodides and aryl bromides. J. Org. Chem..

[52] Cunico RF, Maity BC (2002). Direct carbamoylation of aryl halides. Org. Lett..

[53] Wu X-F, Neumann H, Beller M (2013). Synthesis of heterocycles via palladium-catalyzed carbonylations. Chem. Rev..

