# Supplementary material for: Tandem utilization of CO2 photoreduction products for the carbonylation of aryl iodides
Source: Nat Commun. 2022 May 26;13:2964. doi: 10.1038/s41467-022-30676-y (PMC9135707; doi:10.1038/s41467-022-30676-y)
Supplement: Supplementary file 1 — Supplementary Information [file 41467_2022_30676_MOESM1_ESM.pdf]

Supplementary Information

Tandem utilization of CO_2 photoreduction products for the carbonylation of aryl iodides

Yuan-Sheng Xia[1], Meizhong Tang[1], Lei Zhang[2], Jiang Liu*[1,2], Cheng Jiang[1], Guang-Kuo Gao[1], Long-Zhang Dong[1], Lan-Gui Xie*[1] and Ya-Qian Lan[1,2]*

[1]Jiangsu Collaborative Innovation Centre of Biomedical Functional Materials, Jiangsu Key Laboratory of New Power Batteries, School of Chemistry and Materials Science, Nanjing Normal University, Nanjing 210023, P. R. China.

[2]School of Chemistry, South China Normal University, Guangzhou, 510006, P. R. China.

Correspondence and requests for materials should be addressed to Y.-Q. L. (Email: yqlan@njnu.edu.cn; Homepage: http://www.yqlangroup.com)

Supplementary Figures

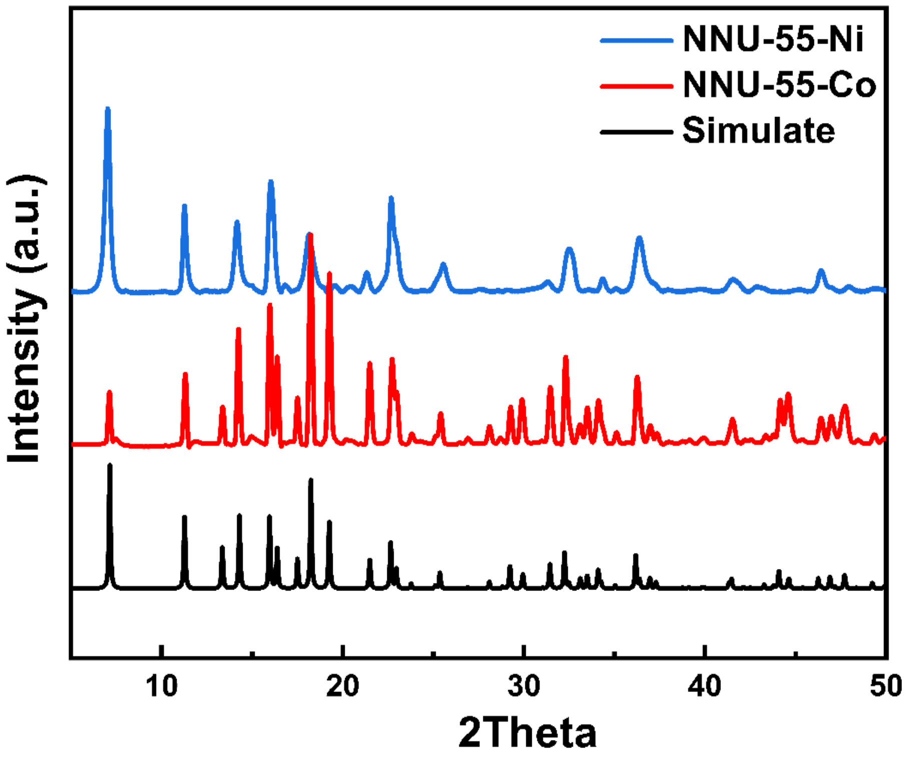

Supplementary Figure 1. PXRD patterns of NNU-55-M.

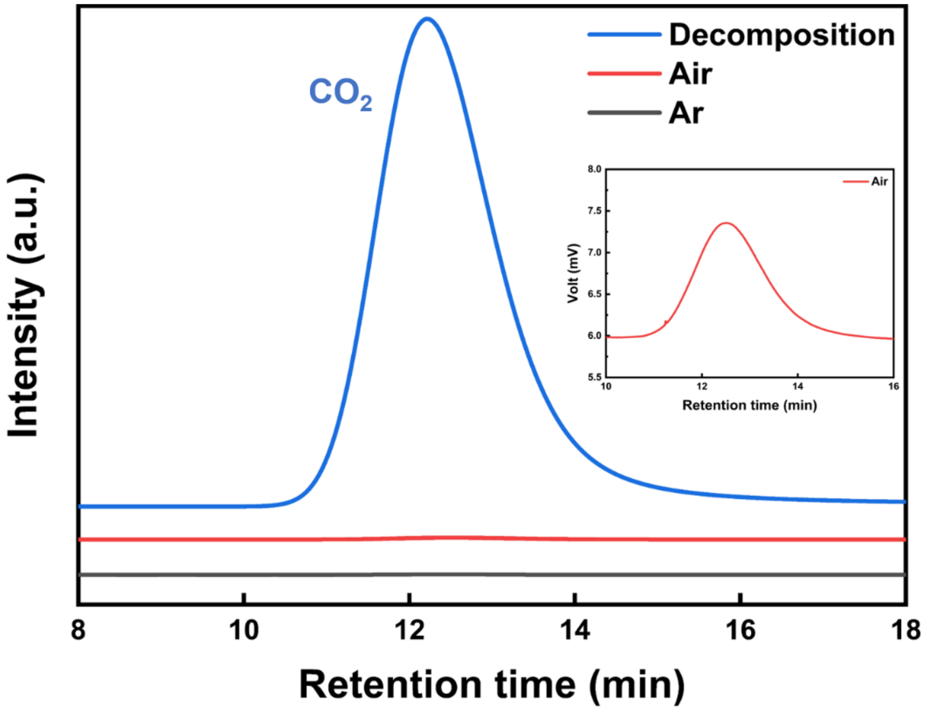

Supplementary Figure 2. GC measurement for CO_2 gas released from NNU-55 (blue), air (red) and Ar (gray).

The presence of a CO_2 molecule is confirmed by GC and MS analysis. According to a reported method, destruction of the framework of NNU-55-M by strong inorganic acid thereby released the coordinated CO_2 molecules. In order to compare, the CO_2 concentration was tested under air and argon atmosphere, respectively. 50 mg amounts of crystals were put into a sealed tube under argon and concentrated hydrochloric acid was added. Gas chromatography analyses of gas sampled from the headspace during the crystals were decomposed.

Owing to only the fewer raw materials being added, it is easy for a clearer result to analyze the reaction solution with GC-MS. We can find any evidence of decomposition products. The solution was tested directly by MS after the reaction. The comparison was performed by GC-MS and saturated CO_2 solution indicating the presence of CO_2 in the reaction solution. In addition, the solution produced other new substances of amides solvent breakdown, for example, some dimethylamine.

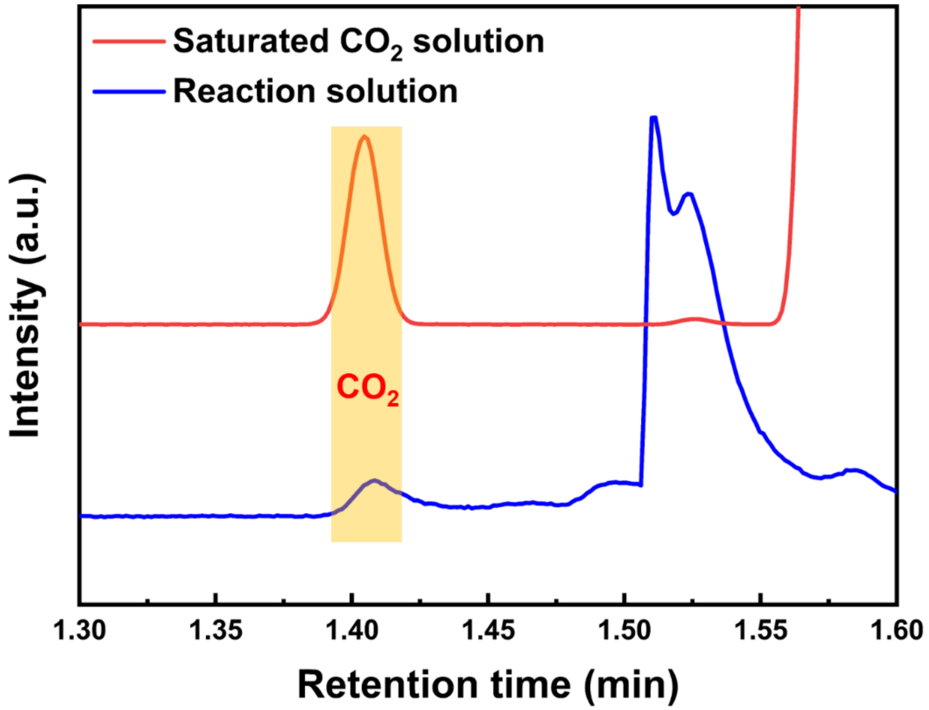

Supplementary Figure 3. GC-MS measurement for reaction solution of NNU-55 (blue) and saturated CO_2 solution (red).

According to a previous report, the amides solvent hydrolyzes into formic acid and dimethylamine at elevated temperatures. Then, formic acid can decompose to CO_2 and other products[10].

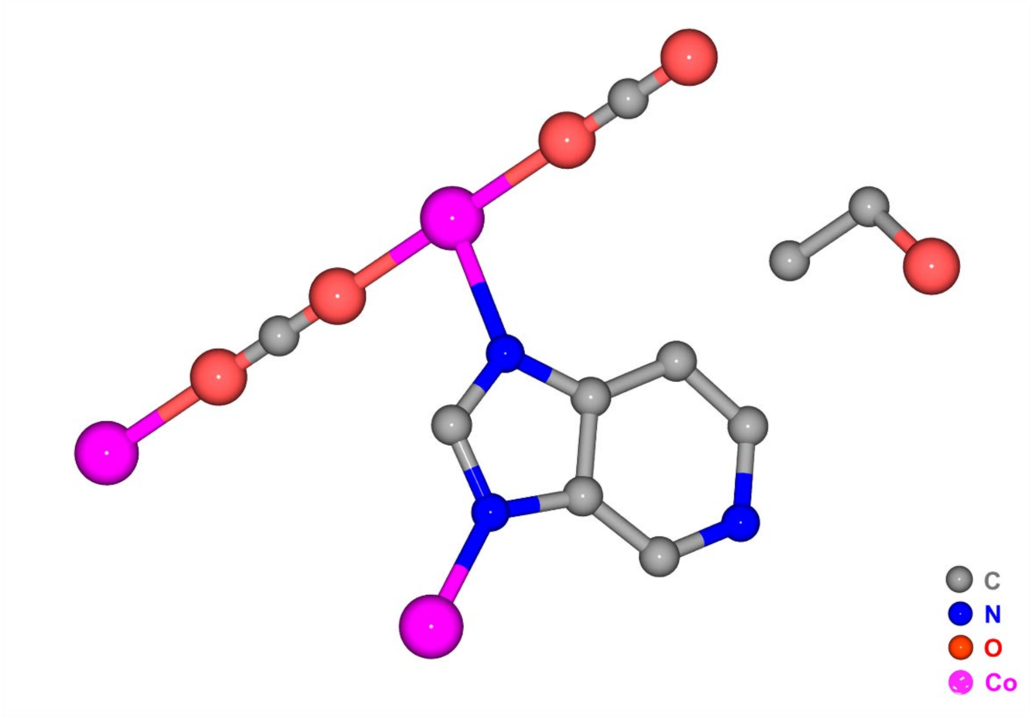

Supplementary Figure 4. Asymmetric units of the NNU-55-Co.

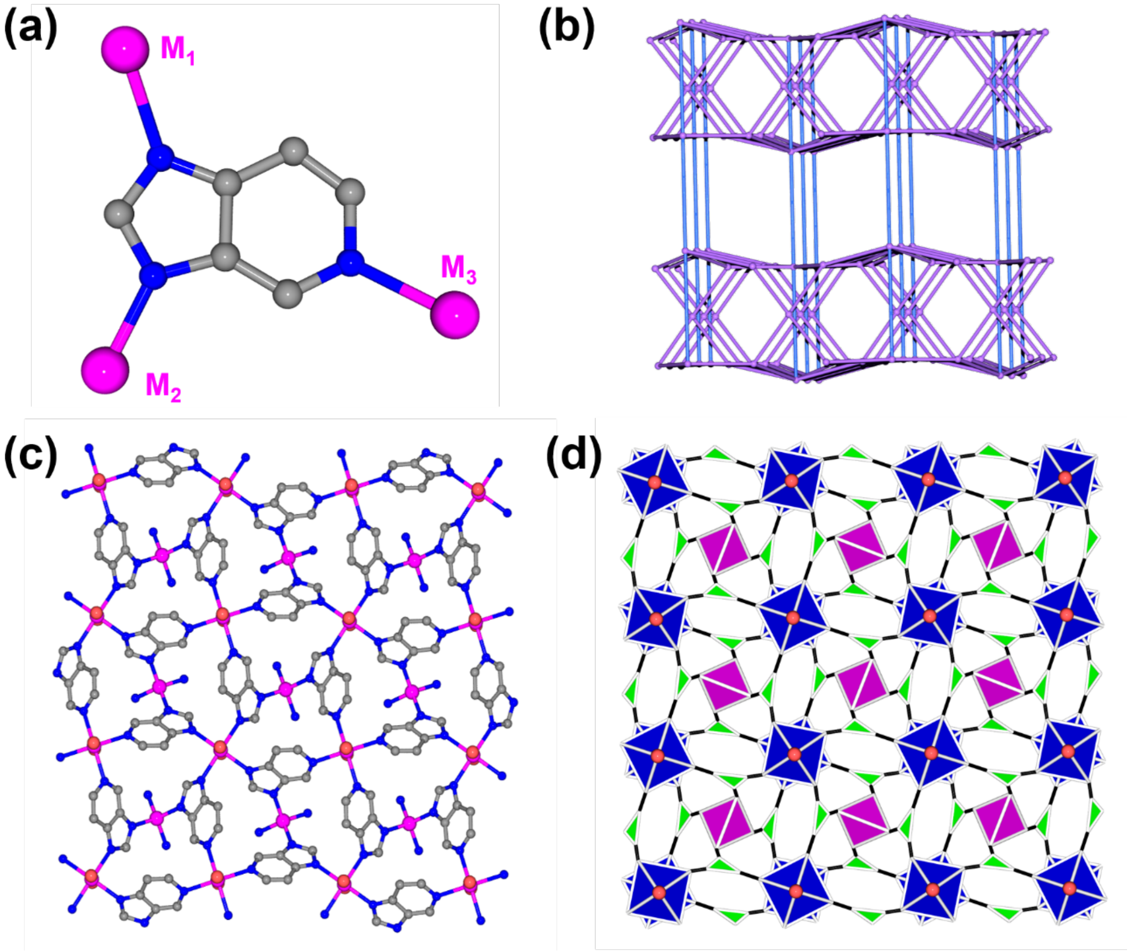

Supplementary Figure 5. (a). The coordination modes of 5-AD. and (b). The topological structure of NNU-55. (c). Schematic of the monolayer structure of a double layer two-dimensional structure. (d). Illustrating the coordination polyhedral of each atom in the c-axis direction of the crystal.

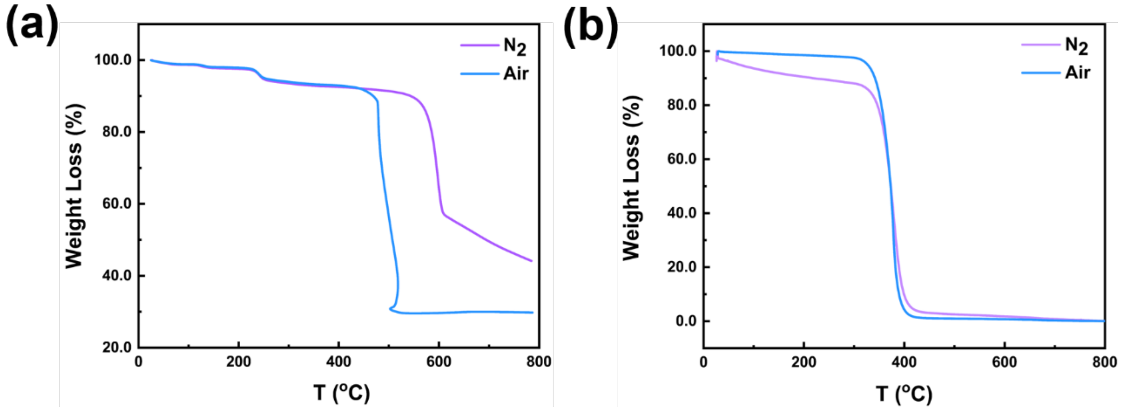

Supplementary Figure 6. Thermogravimetric analysis of (a). NNU-55-Ni and (b). NNU-55-Co.

The thermogravimetric analysis of NNU-55-M was carried out under oxygen or nitrogen atmosphere at a rate of 20 °C min^{-1} from room temperature to 800 °C. It is clear that the structures of these MOFs can keep high thermostability.

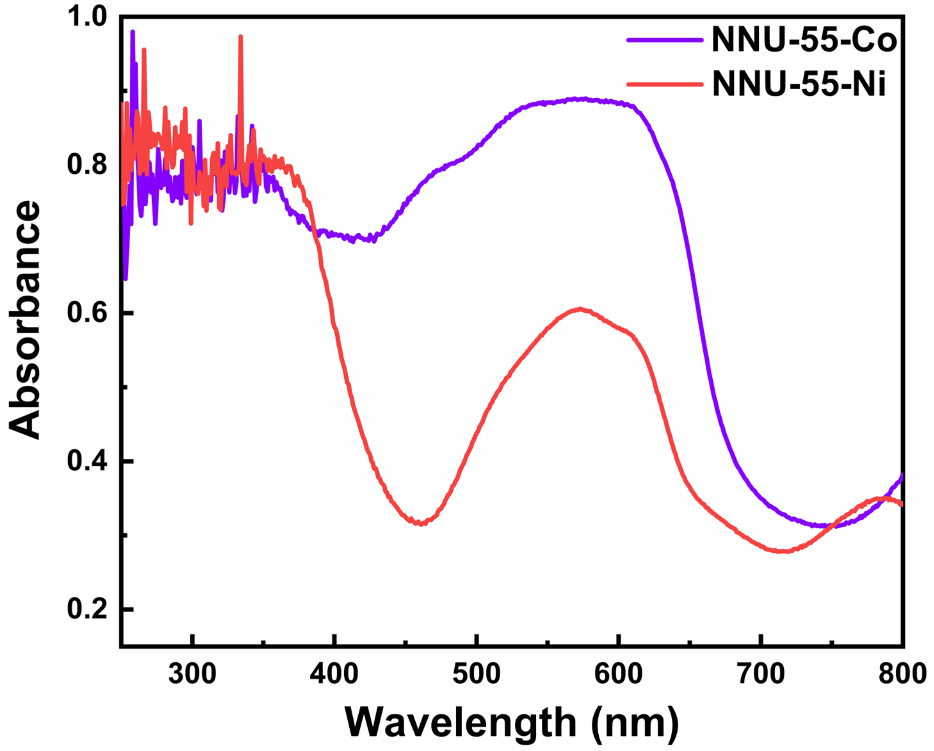

Supplementary Figure 7. The UV−Vis absorption spectra of NNU-55-Ni and NNU-55-Co.

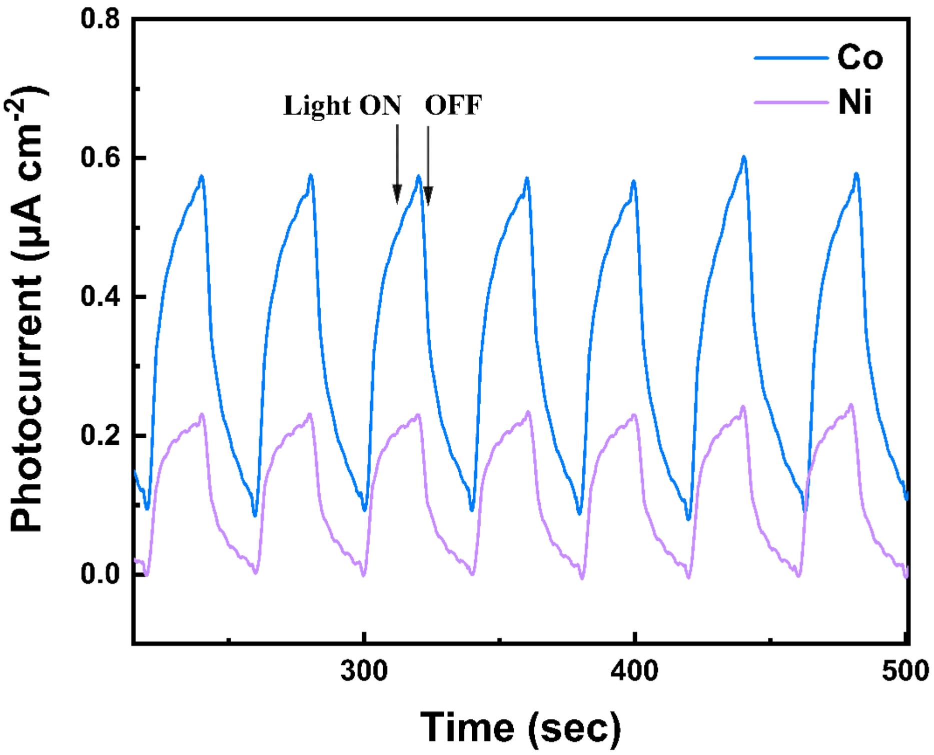

Supplementary Figure 8. The photo-current responses for NNU-55-M.

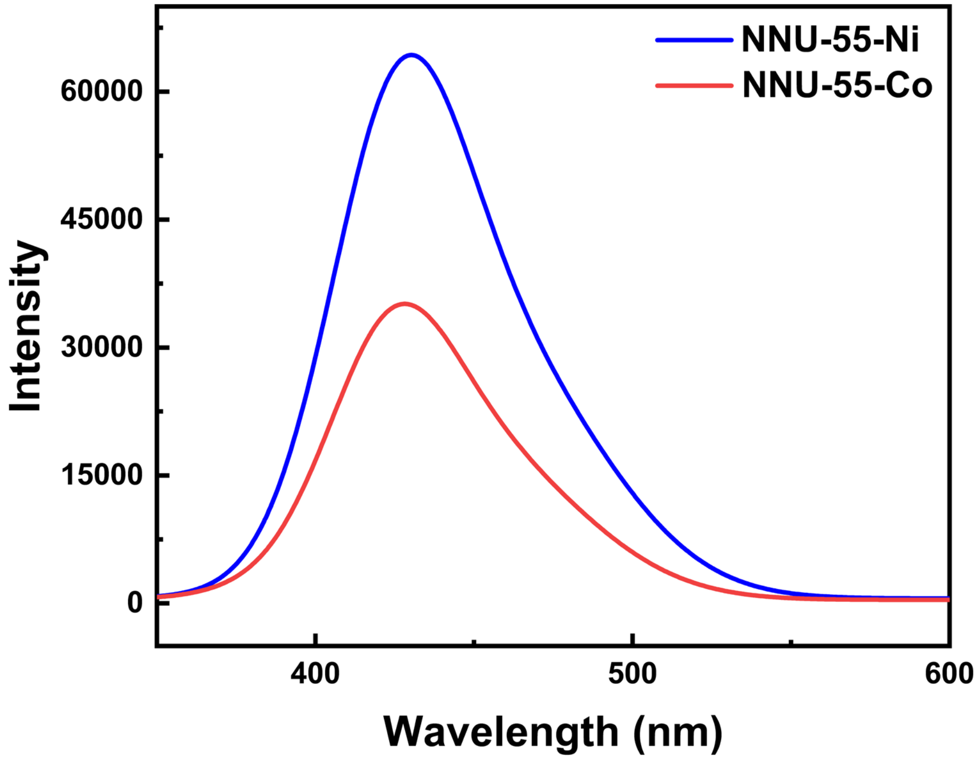

Supplementary Figure 9. The steady-state fluorescent spectra of NNU-55-Co/Ni powder.

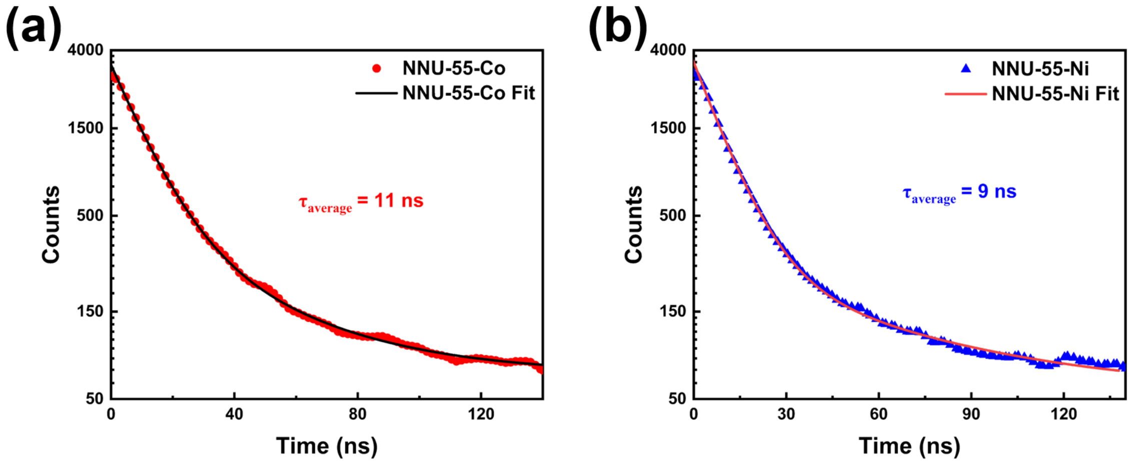

Supplementary Figure 10. Photoluminescence lifetime decay curve of (a). NNU-55-Co, (b). NNU-55-Ni.

We have tested the excited state lifetime of NNU-55-Ni/Co powder and the emission spectrum of the catalyst. These samples were excited by the incident light of 330 nm and the photoluminescence lifetime decay spectra at 430 nm were monitored using Edinburgh FLS980. The fitted parameters for photoluminescence lifetimes were presented in Supplementary Table 3. And the excitation wavelength was 330 nm when steady-state fluorescence spectra was measured using Edinburgh FLS980.

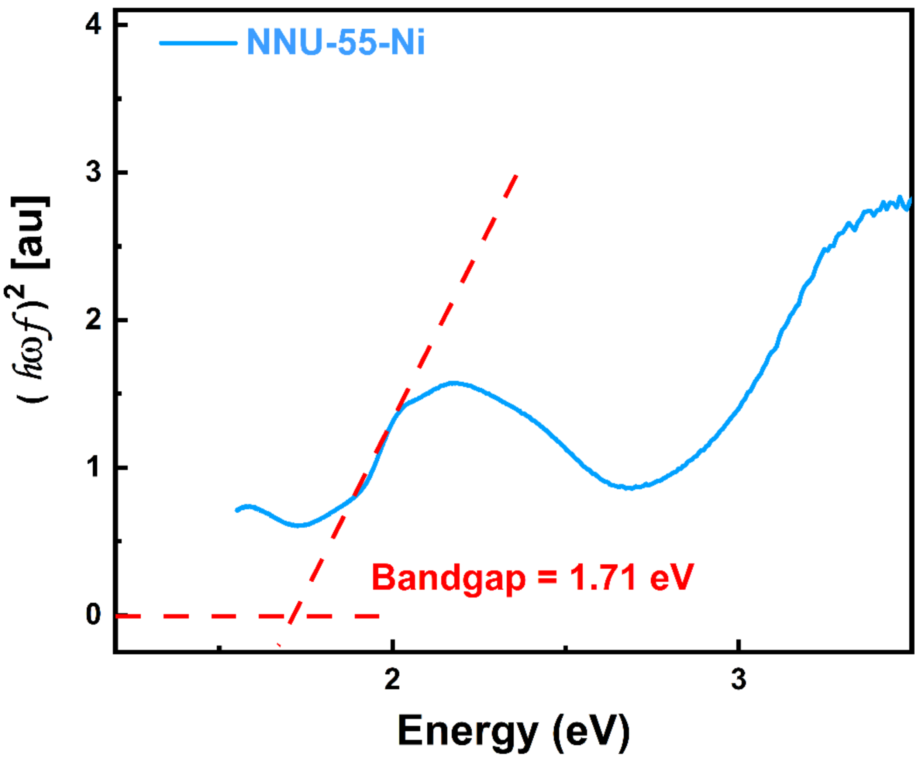

Supplementary Figure 11. Tauc plot of NNU-55-Ni.

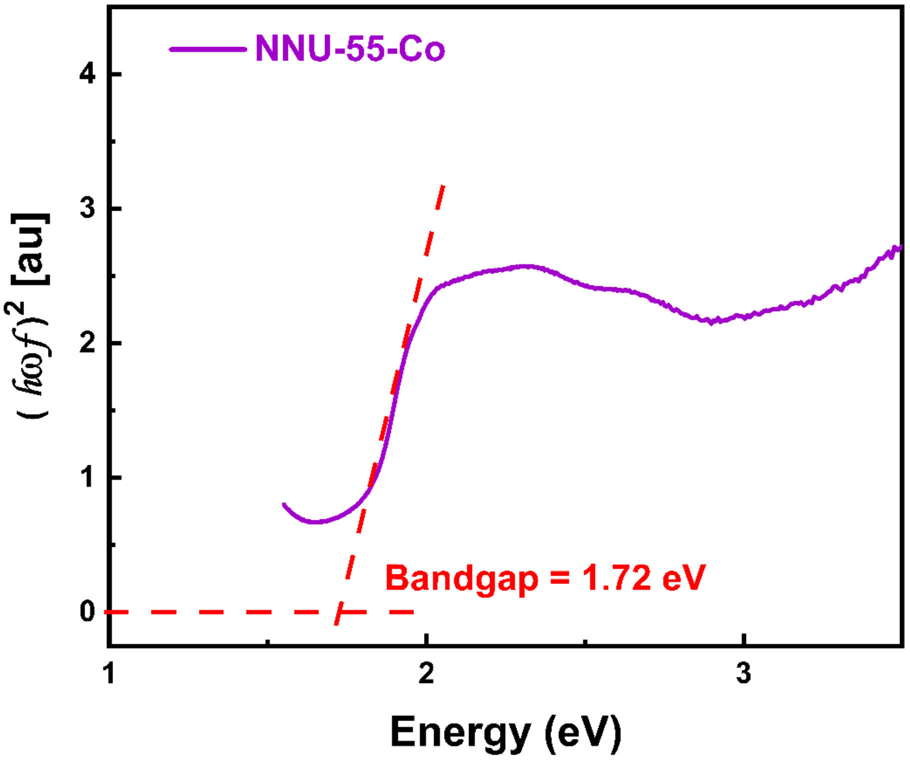

Supplementary Figure 12. Tauc plot of NNU-55-Co.

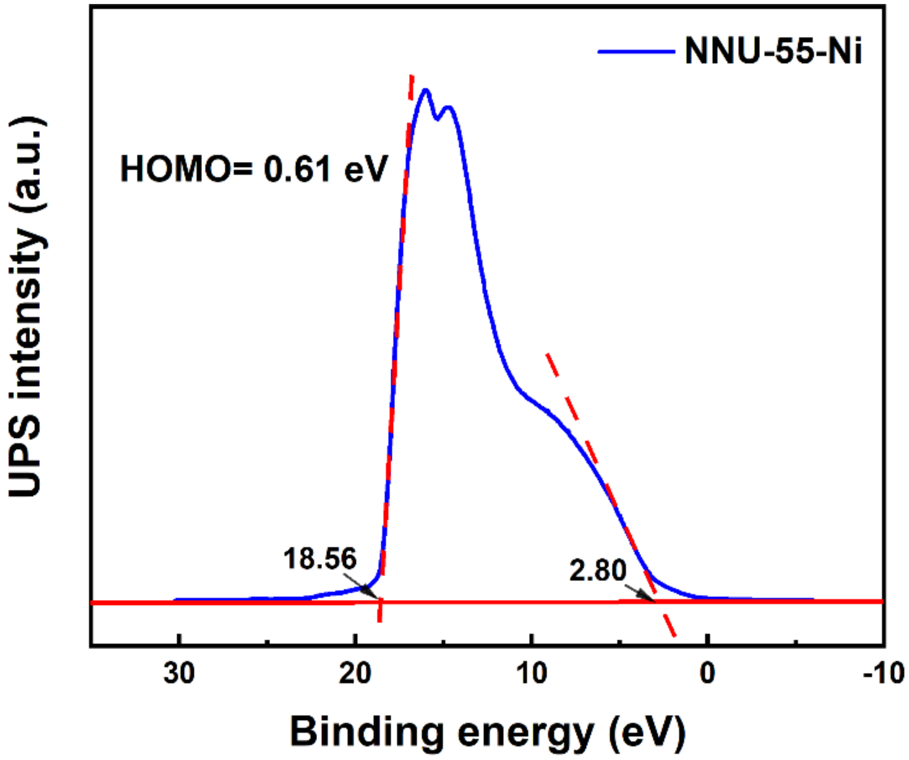

Supplementary Figure 13. UPS spectra of NNU-55-Ni.

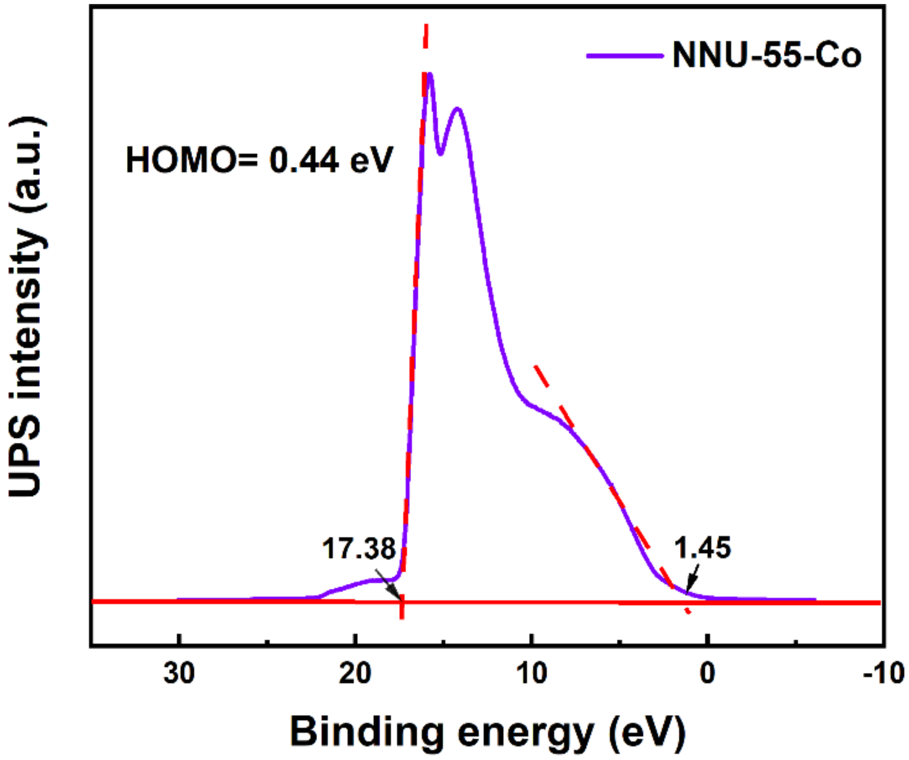

Supplementary Figure 14. UPS spectra of NNU-55-Co.

The ultraviolet photoelectron spectroscopy (UPS) was used to determine the ionization potential (HOMO) of NNU-55-M, which was calculated by subtracting the width of the He I UPS spectra from the excitation energy (21.22 eV). The tangent of the curve intersects the red baseline. The difference between the two crossing point is the width of the He I UPS spectra. Then the ionization potential (HOMO) of NNU-55-M is calculated to be 0.61 eV (NNU-55-Ni) and 0.44 eV (NNU-55-Co).

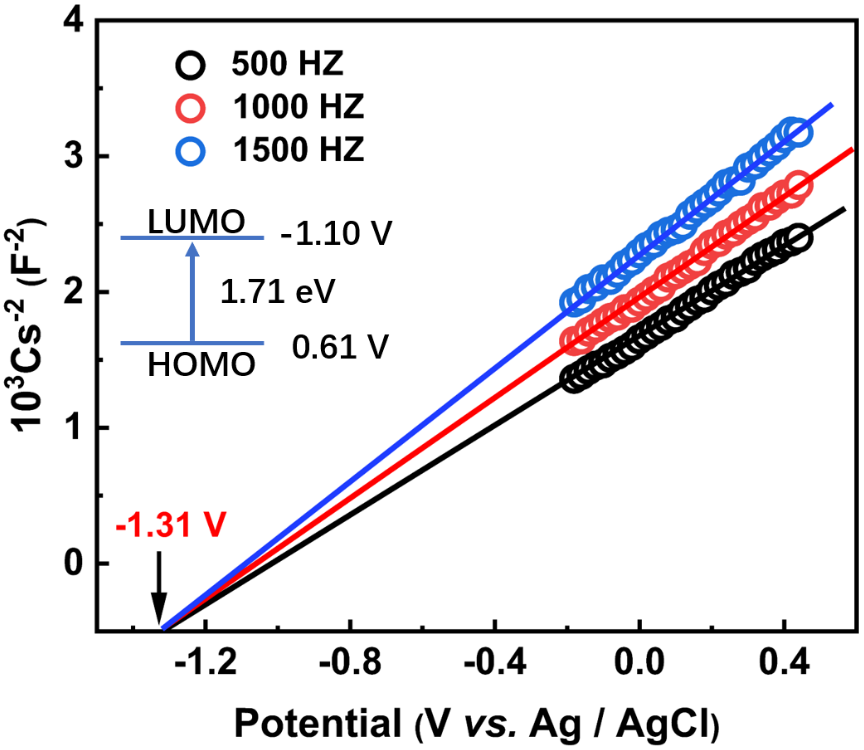

Supplementary Figure 15. Mott-Schottky plots of NNU-55-Ni.

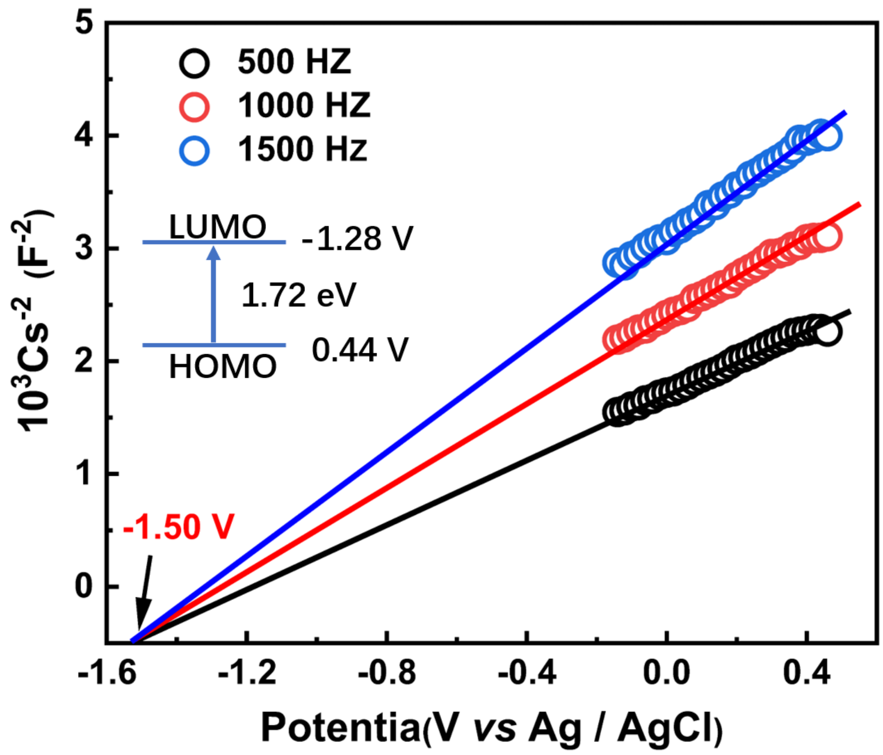

Supplementary Figure 16. Mott-Schottky plots of NNU-55-Co.

The conduction band minima (CBMs) of NNU-55-M MOFs were determined to be -1.10 eV (Ni) and -1.28 eV (Co) (vs. NHE, pH=7). The value of Mott-Schottky was compared with UPS calculated, the value of LUMO were virtually no differences between the two. These data confirmed the high accuracy of results.

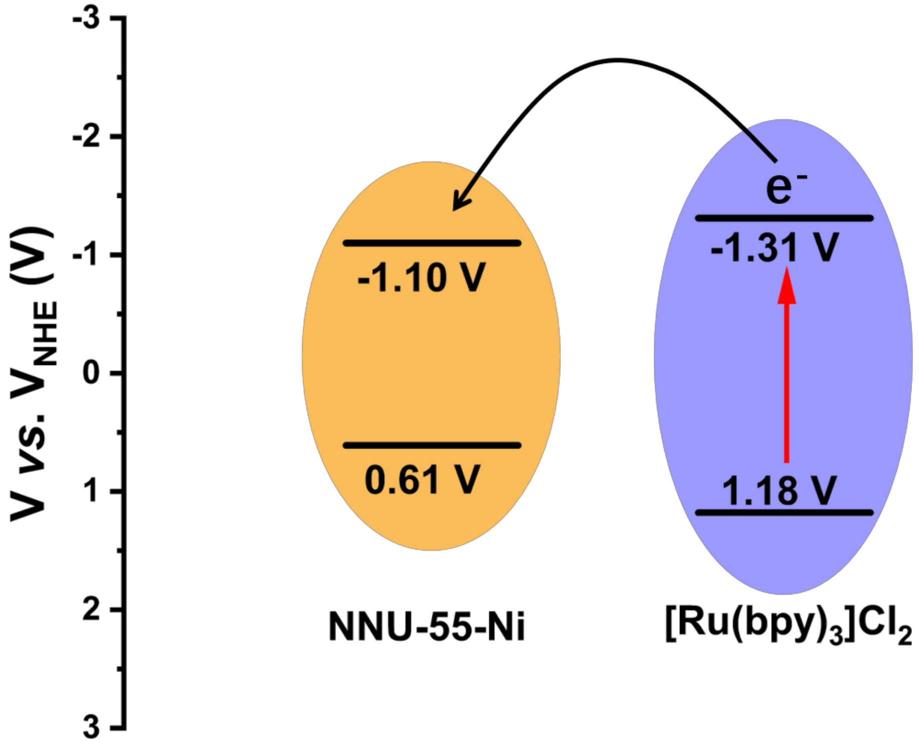

Supplementary Figure 17. Schematic energy-level diagram showing electron transfer from $[Ru(bpy)_3]Cl_2$ to NNU-55-Ni.

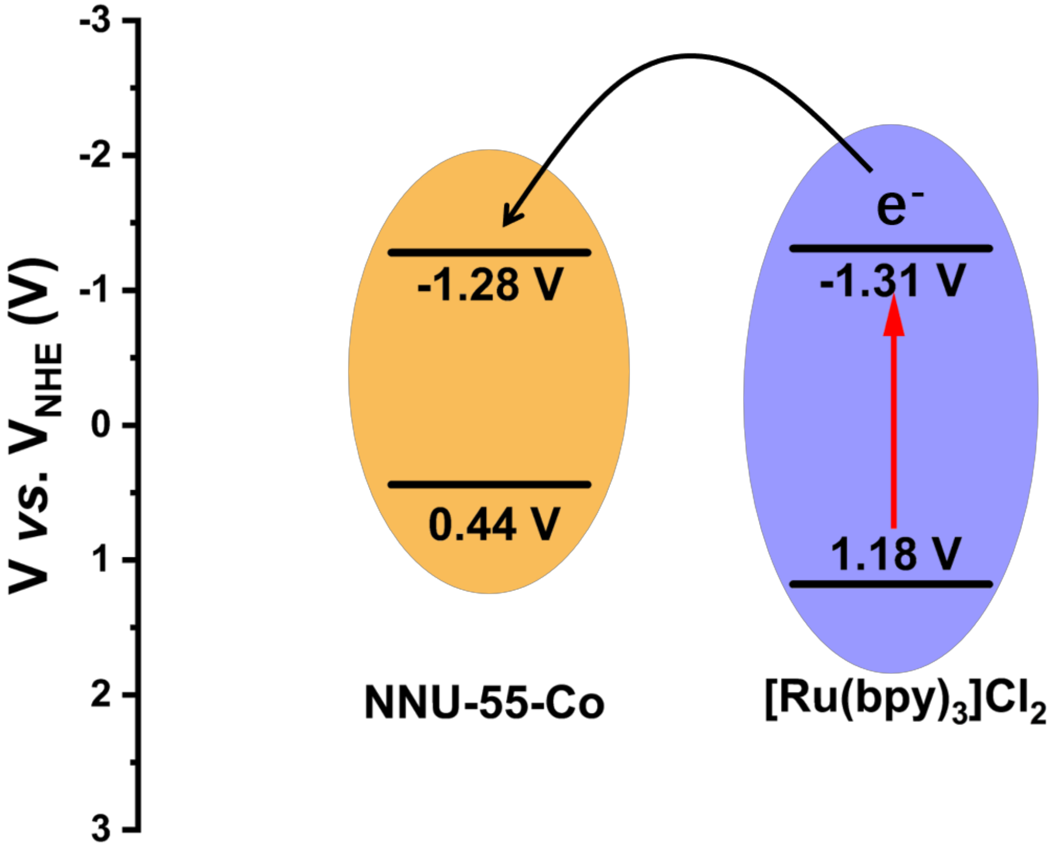

Supplementary Figure 18. Schematic energy-level diagram showing electron transfer from $[Ru(bpy)_3]Cl_2$ to NNU-55-Co.

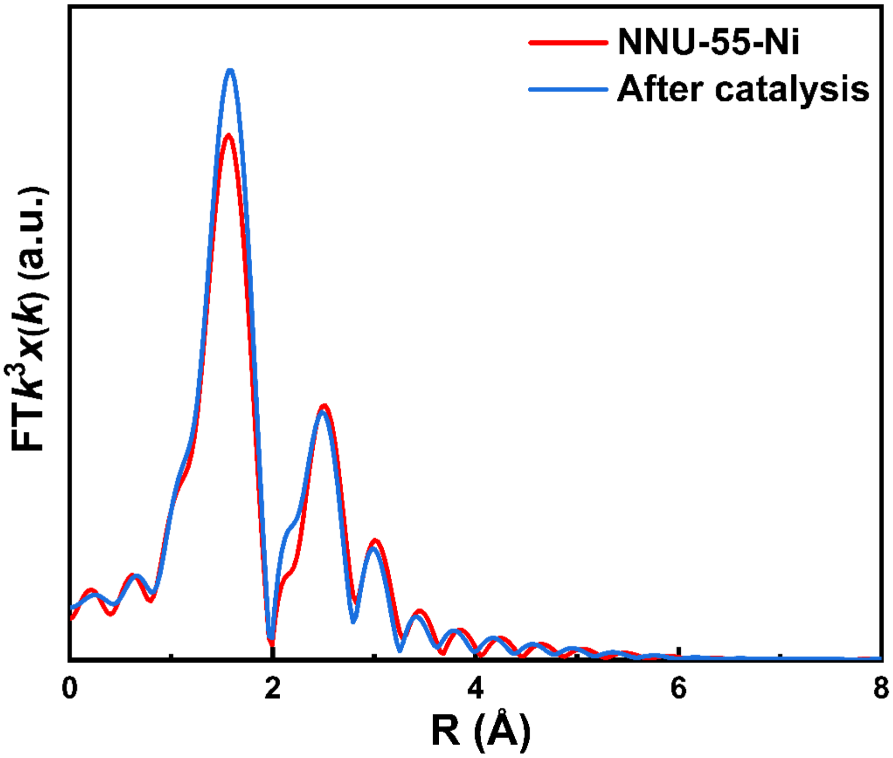

Supplementary Figure 19. The k3-weighted Fourier-transform experimental EXAFS spectrum of catalysts at Ni K-edge.

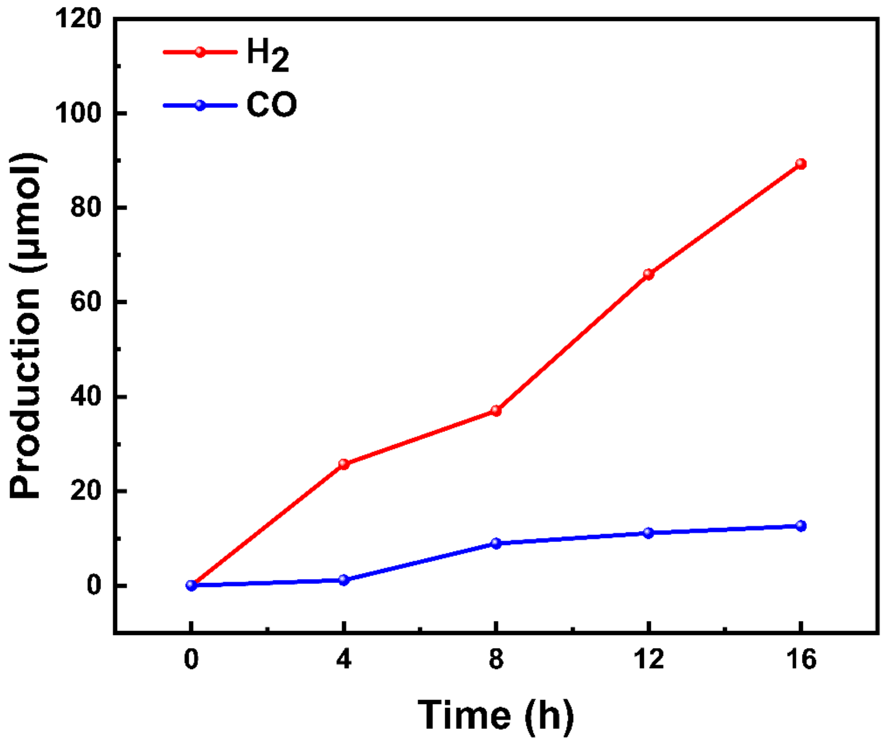

Supplementary Figure 20. The production of CO and H_2 with NNU-55-Co catalyst as a function of reaction time under visible light illumination.

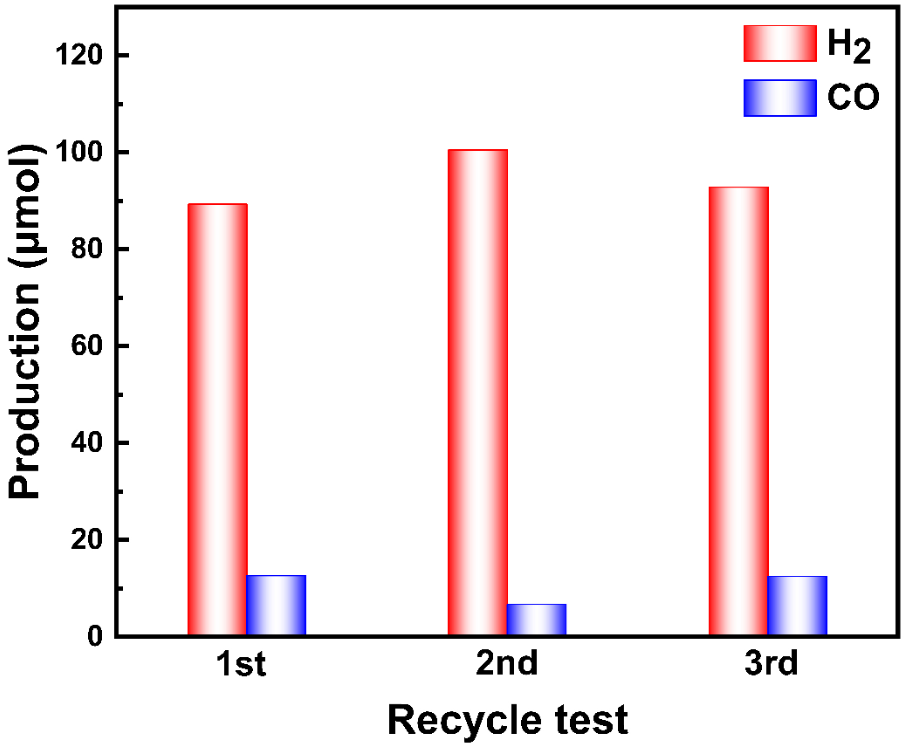

Supplementary Figure 21. The recycle experiment of NNU-55-Co.

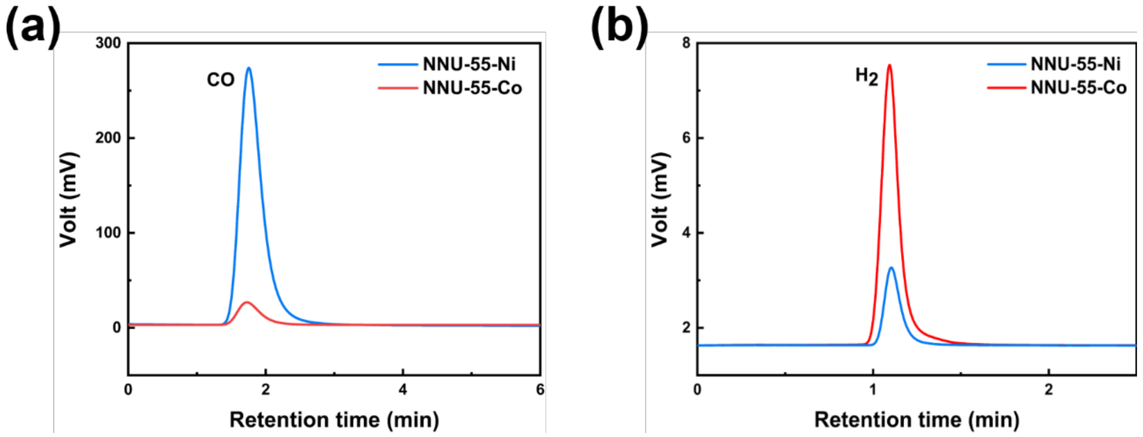

Supplementary Figure 22. Gas chromatography profiles for the gas products from the photoreduction of CO_2 by using NNU-55-Ni and NNU-55-Co as catalysts. (a). CO product can be detected by GC using FID with methanizer and (b). H_2 product can be detected by GC using TCD.

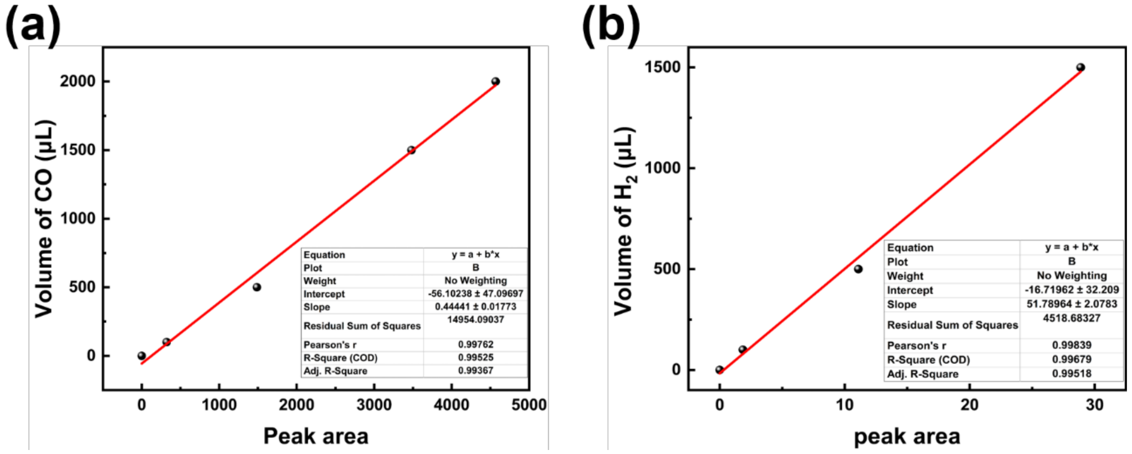

Supplementary Figure 23. A linear relationship between the concentration of (a). CO and (b). H_2, the GC peak areas which were determined by external standard method using highly purified CO or H_2 samples.

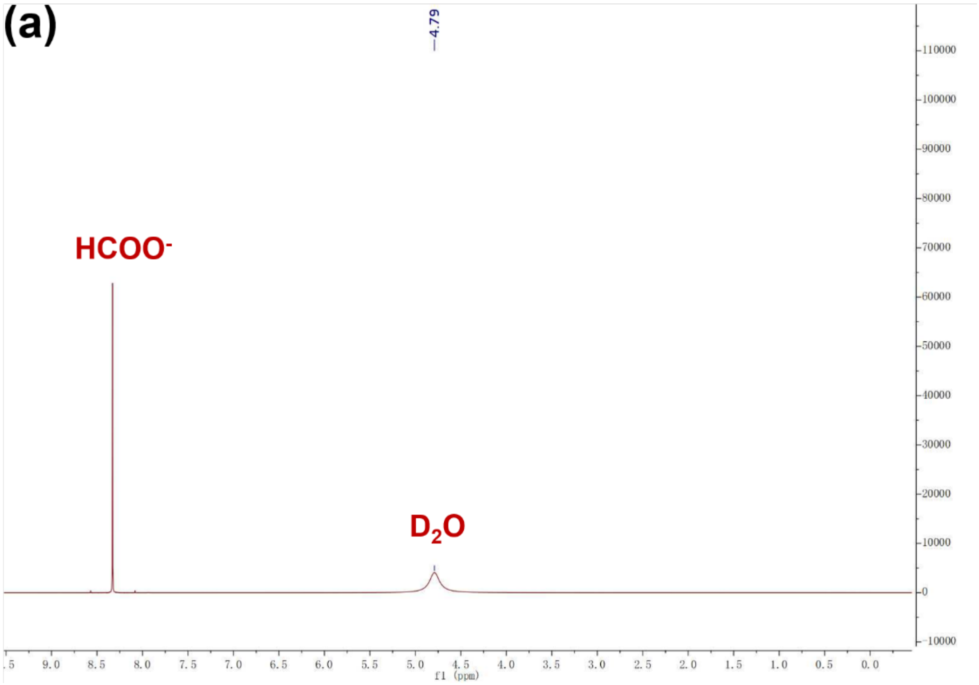
(a)
4.79
HCOO-
D2O
f1 (ppm)

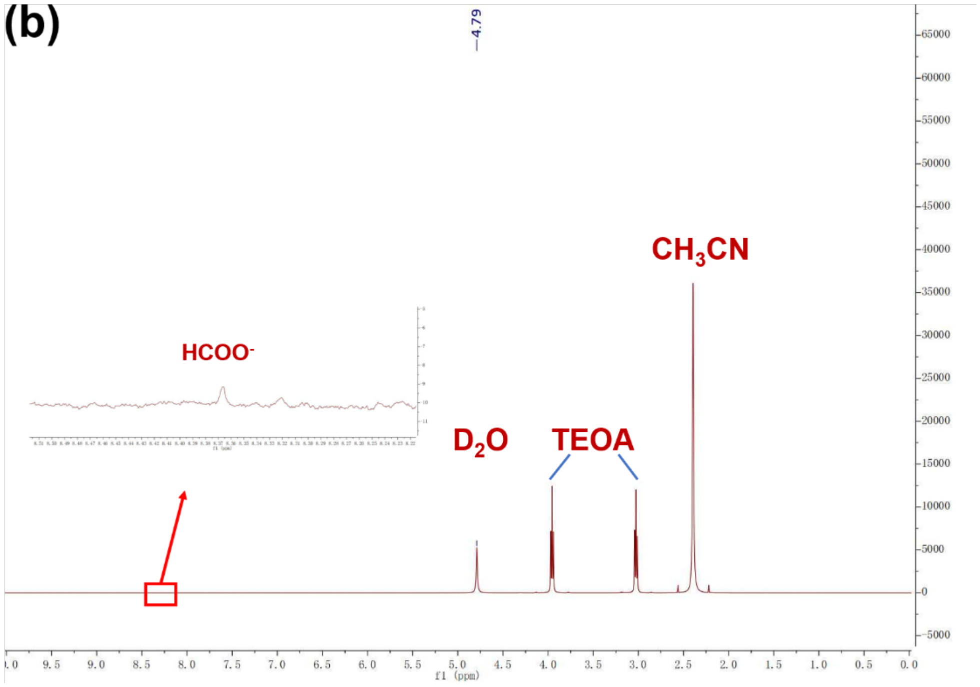
(b)
4.79
CH3CN
HCOO-
D2O
TEOA
f1 (ppm)

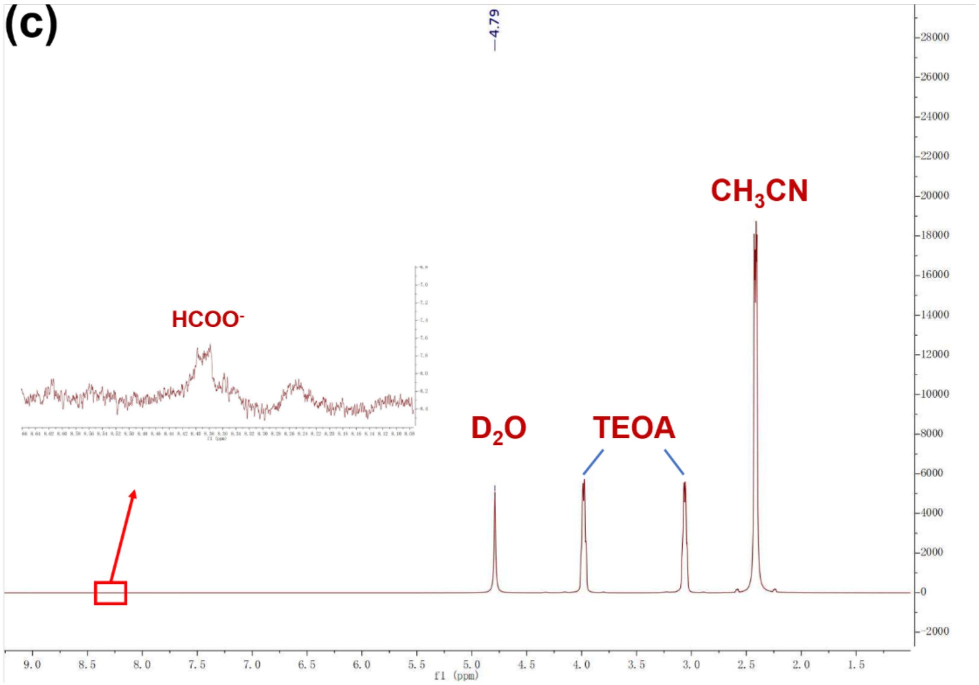

Supplementary Figure 24. The 1H NMR spectra of the liquid phases after the photocatalytic experiments for (a). NH_4COOH, (b). NNU-55-Ni and (c). NNU-55-Co.

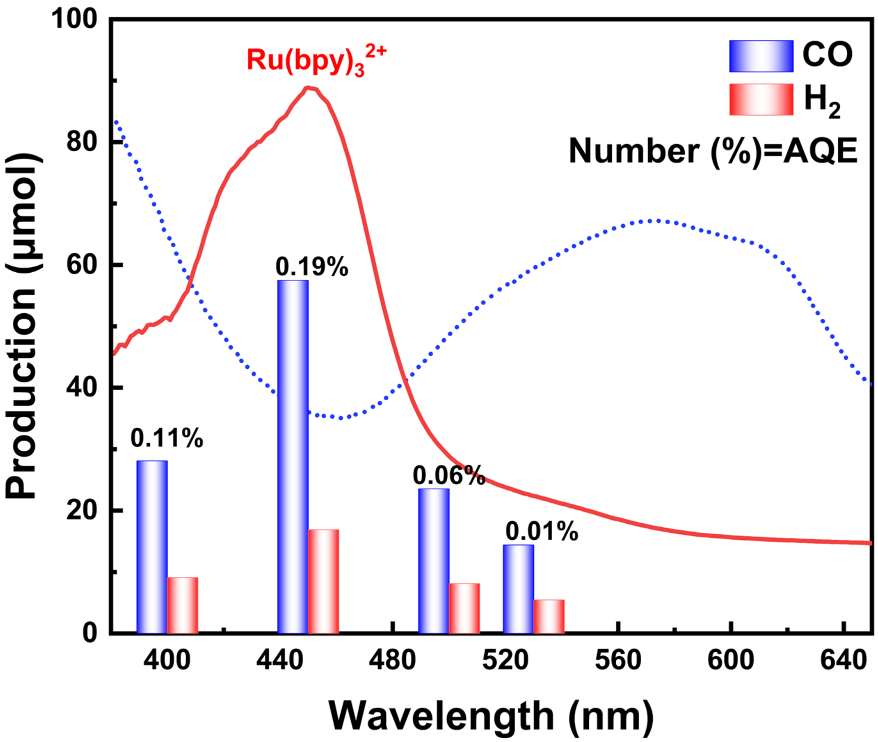

Supplementary Figure 25. The dependence of the wavelength of incident light on the evolution of CO and H_2. The bar diagram represents the amount of CO and H_2 after 16 h irradiation. The number represents the AQE of Photocatalytic products (CO and H_2). The red (blue) line is the UV-Vis absorption spectrum of photosensitizer $[Ru(bpy)_3]Cl_2$ (NNU-55-Ni).

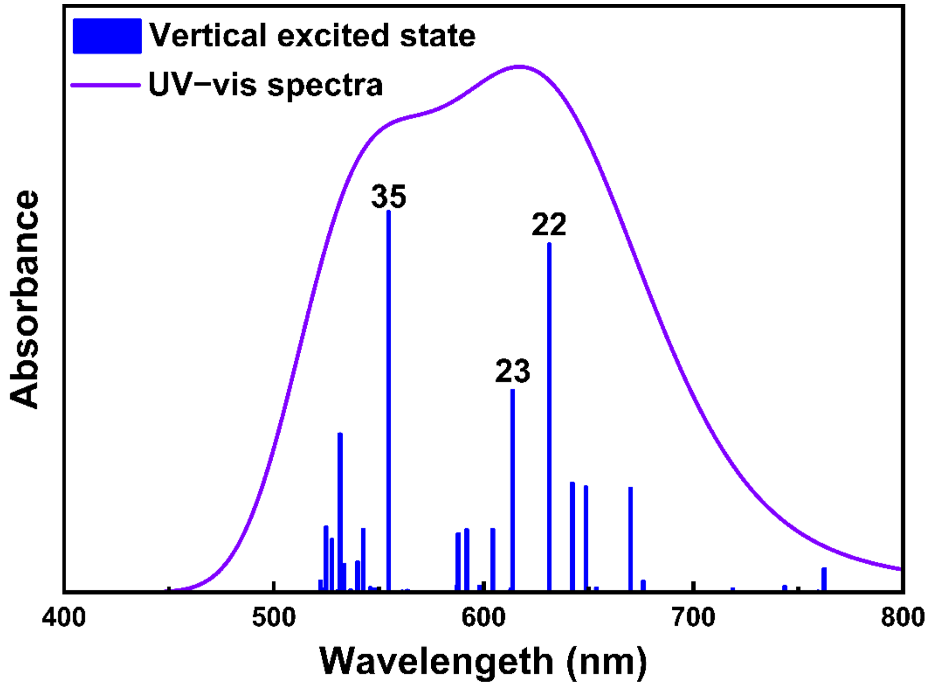

Supplementary Figure 26. The theoretical simulated UV-vis absorption and the signatures of electronically excited states of NNU-55-Ni-NS by TD-DFT.

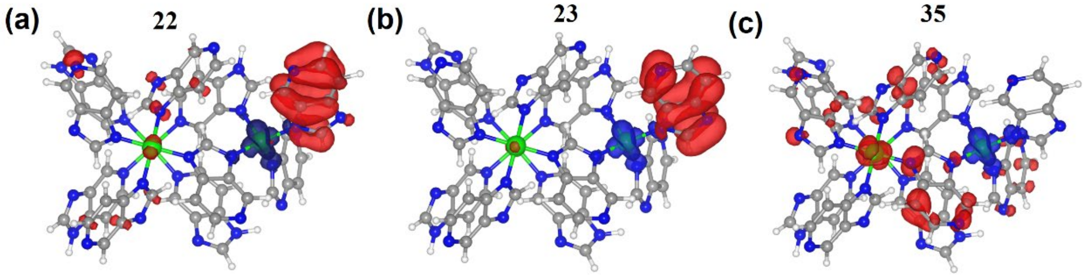

Supplementary Figure 27. Charge density difference corresponding to different low-lying excitations of NNU-55-Ni-NS, where the red and blue represent electron depletion and accumulation, respectively. (a). 22. (b). 23. (c). 35.

To further explore the excitation properties of the system, we perform time-dependent DFT (TDDFT) calculations with 50 low-lying excitations, which computes the signatures of electronically excited states and simulates the UV-vis spectra. As shown in **Supplementary Fig. 26**, the calculation results agree well with the experiments, suggesting the reliability of the computation methods and models. In the following, electron excitation analysis is performed to characterize the absorption peaks with the help

of Multiwfn, and three low-lying excitations with the strongest excitation intensities are selected. **Supplementary Fig. 27** illustrates the charge density difference between the ground state and the excited state, and the photo-excited electrons are marked as red while the holes are denoted as blue. It can be noted the Ni centers serve as electron donors and the surrounding ligands accept the photo-excited electrons for the three selected excitations, and we attribute these excitations to MLCT. Granted, the excitations in the UV-vis regime can be very complicated and involve many kinds of change transfer processes. However, we can conclude here, based on the calculation results and analysis, that the observed peaks around 400-800 nm are mainly dominated by MLCT.

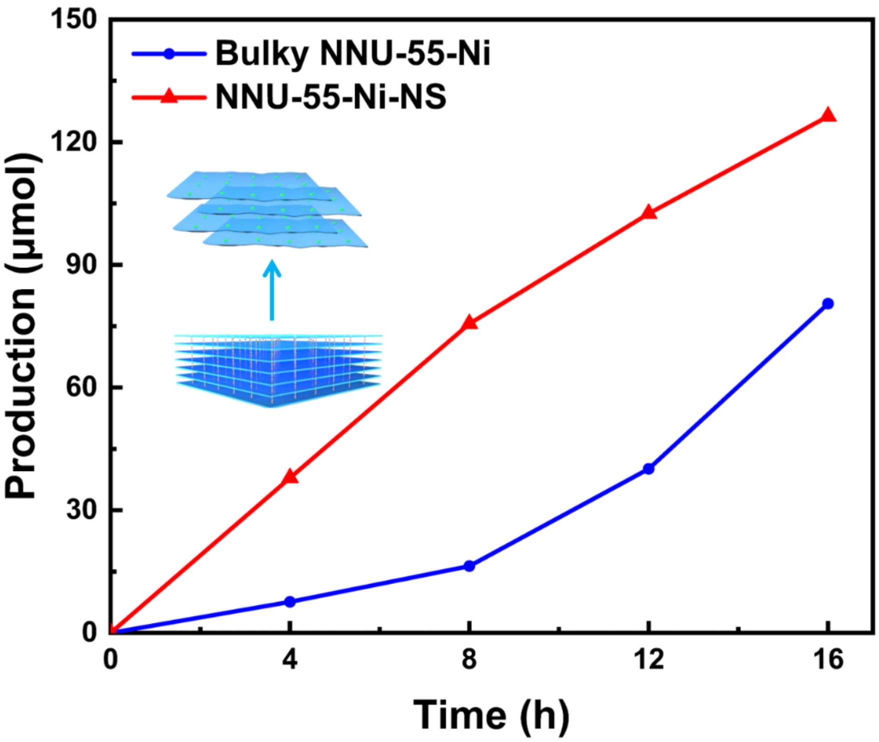

Supplementary Figure 28. When NNU-55-Ni or NNU-55-Ni-NS were used in the photocatalytic CO_2RR as starting catalyst, a function of the irradiation time of CO production from CO_2 photoreduction.

When NNU-55-Ni-NS was used as catalyst to complete photocatalytic CO_2RR, the CO product formation rate is significantly higher than that of the bulky NNU-55-Ni, especially in the initial stage of the reaction.

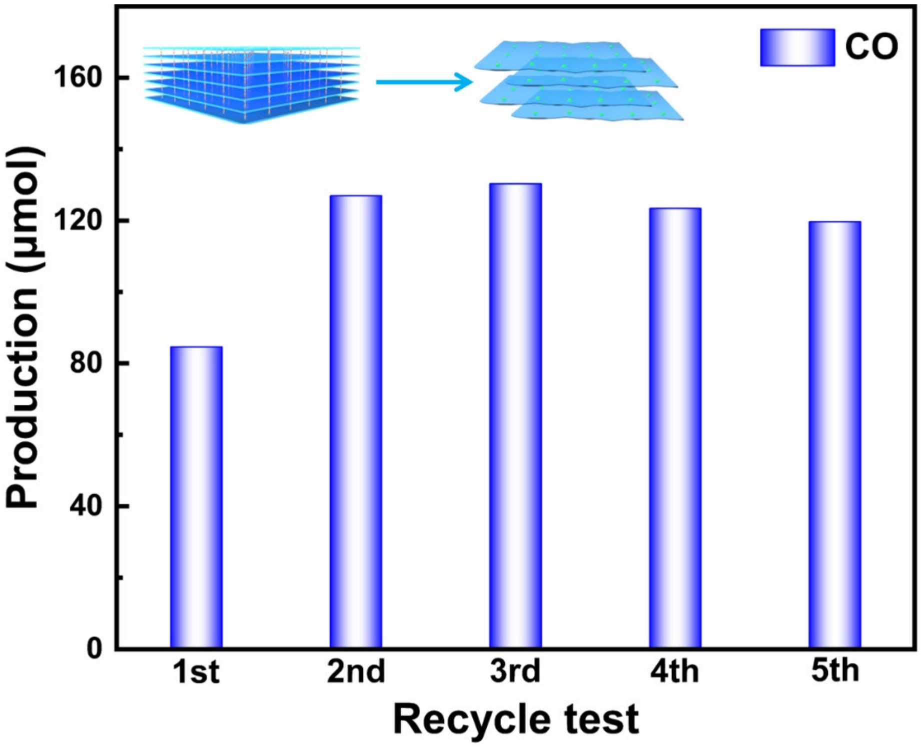

Supplementary Figure 29. The recycle experiment of NNU-55-Ni.

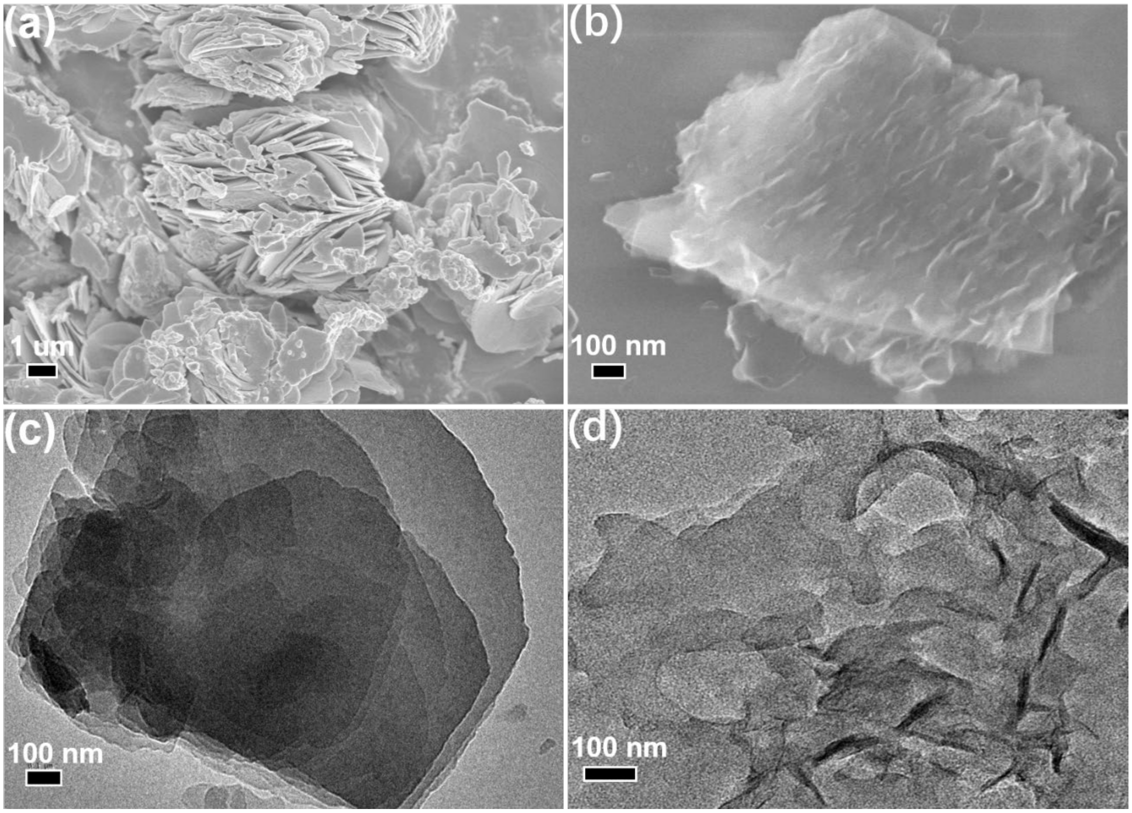

Supplementary Figure 30. (a). SEM image of the grinded NNU-55-Ni. **(b).** SEM image of NNU-55-Ni after photocatalytic reaction. **(c).** TEM image of the grinded NNU-55-Ni. **(d).** TEM image of NNU-55-Ni after photocatalytic reaction.

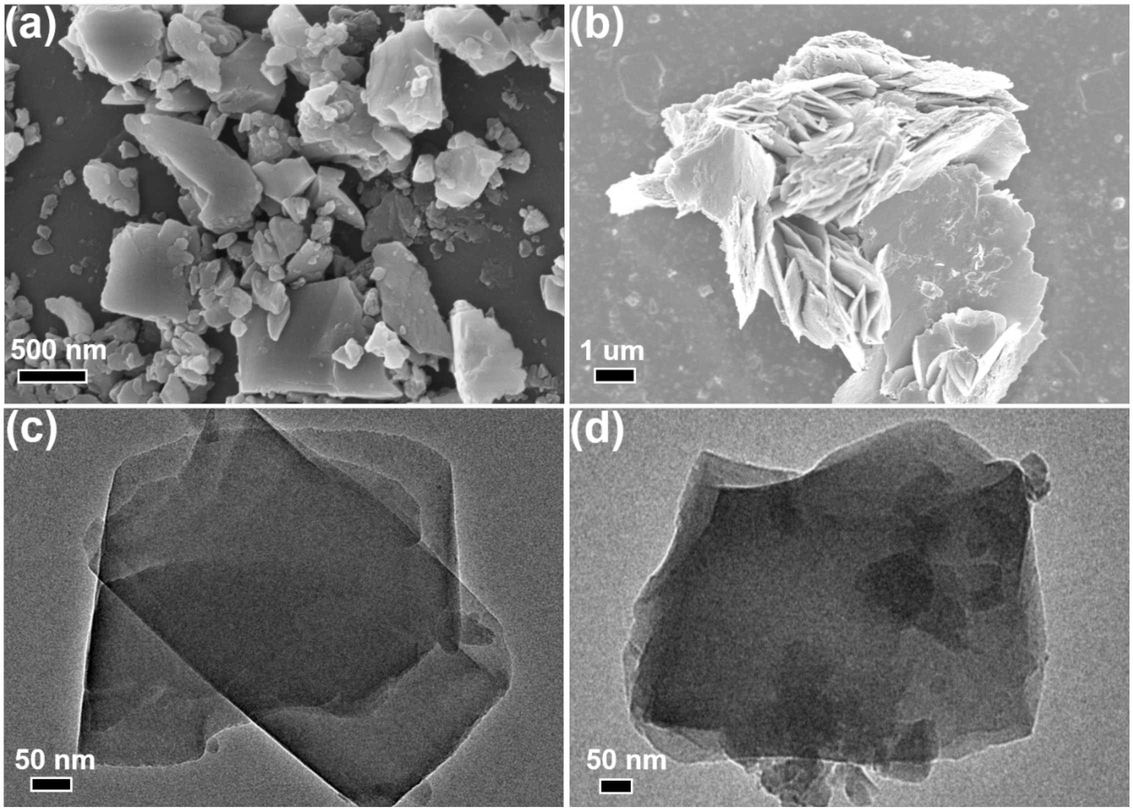

Supplementary Figure 31. (a). SEM image of the grinded NNU-55-Co. **(b).** SEM image of NNU-55-Co after photocatalytic reaction. **(c).** TEM image of the grinded NNU-55-Co. **(d).** TEM image of NNU-55-Co after photocatalytic reaction.

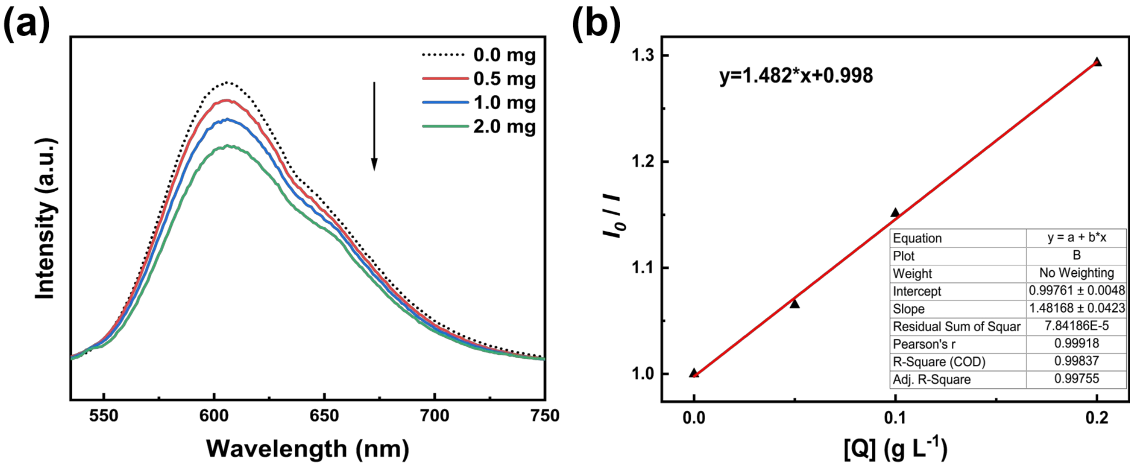

Supplementary Figure 32. (a). The steady-state fluorescent spectra of [Ru(bpy)$_3$]Cl$_2$ (0.04 mM)upon the addition of increasing amounts of NNU-55-Ni-NS (λex = 400 nm). **(b).** Stern-Volmer plot of the emission quenching of [Ru(bpy)$_3$]Cl$_2$ solution by NNU-55-Ni-NS.

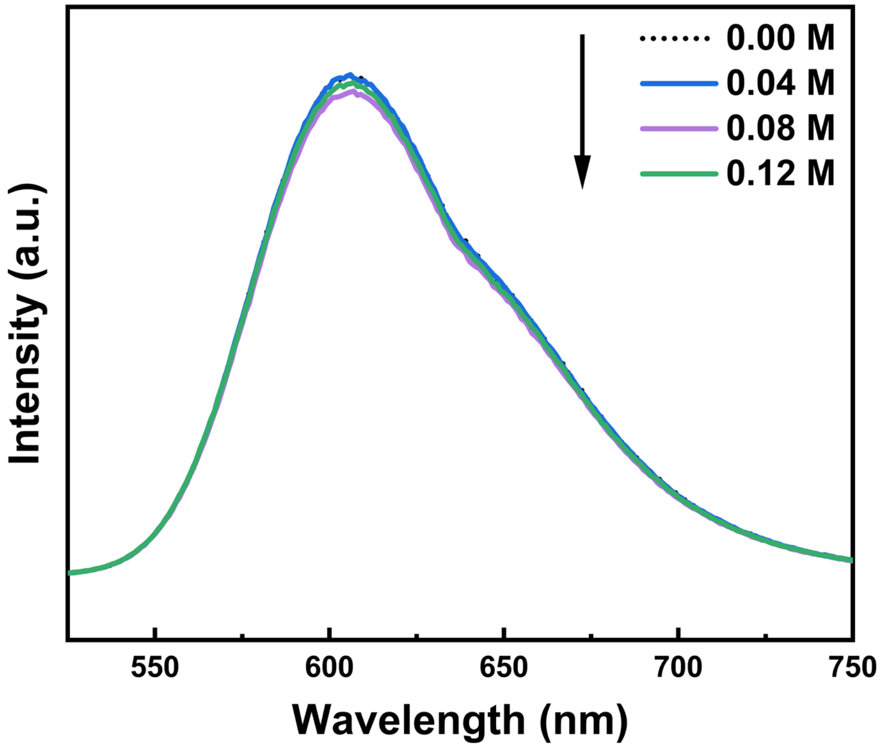

Supplementary Figure 33. Fluorescence intensity of 0.04 mM [Ru(bpy)$_3$]Cl$_2$ in CH_3CN/H_2O solution with the addition of 0, 0.04, 0.08, 0.12 M TEOA.

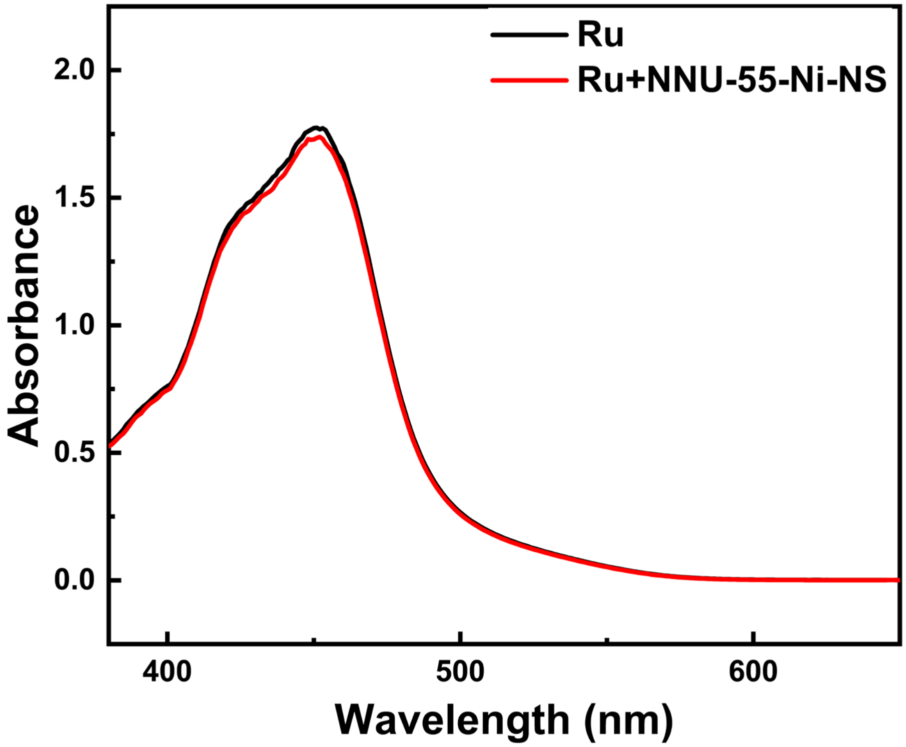

Supplementary Figure 34. UV-vis absorption spectra of $[Ru(bpy)_3]Cl_2$ solution (0.10 mM) and mixture solution containing $[Ru(bpy)_3]Cl_2$ (0.10 mM) and NNU-55-Ni-NS (0.154 g / L).

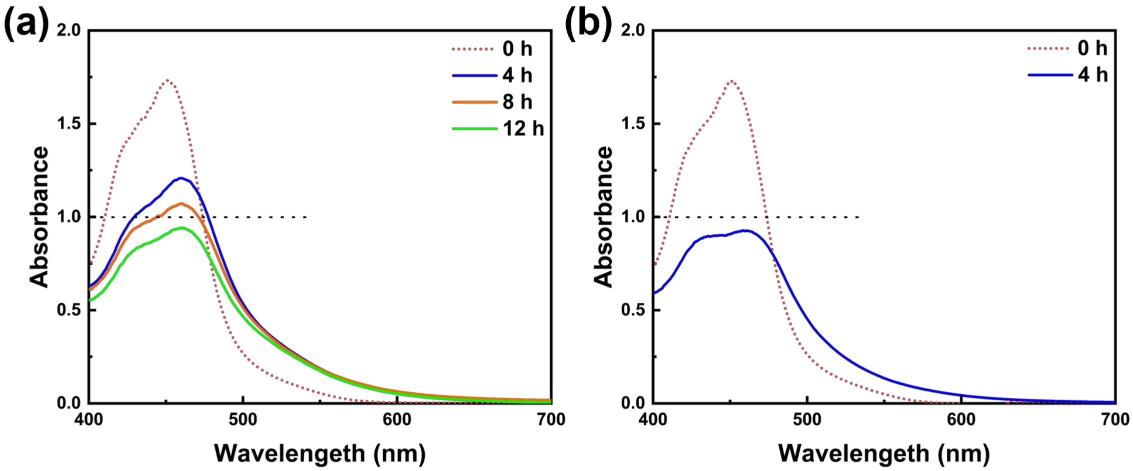

Supplementary Figure 35. UV-vis absorption spectra taken during the course of the photolysis experiment containing 0.1mM $[Ru(bpy)_3]Cl_2$, TEOA in CH_3CN/H_2O upon irradiation with visible light when NNU-55-Ni-NS catalyst was (a). present, or (b). absent.

We measured the UV-vis absorption spectra of a pure Ru solution and a Ru solution containing NNU-55-Ni-NS catalyst (**Supplementary Figure 32**). The results revealed that the addition of catalyst has almost no effect on the inherent light absorption of $[Ru(bpy)_3]Cl_2$ (PS). And the steady-state photoluminescence (PL) spectroscopy of the Ru solution containing different masses of NNU-55-Ni-NS catalysts is characterized (without TEOA), in which the PL intensity of $[Ru(bpy)_3]Cl_2$ decrease as the increased catalyst concentration (**Fig. 3g**). The above two experiments confirmed that the quenching of the PL intensity is attributed to the photoexcited electrons transferred from the Ru photosensitizer to the catalyst. We also tested the UV/vis absorption spectrum changes of the photocatalytic solution with catalyst or catalyst-free under different irradiation time (**Supplementary Figure 35**). It is known from experimental phenomena that in the absence of catalyst (only with the sacrificial agent TEOA), the faster bleaching of PS under visible light is due to the instability of PS^- state formed during the charge transfer process. In contrast, the bleaching of PS became significantly slower in the presence of catalyst, which indicated that effective photo-generated charge transfer occurs between PS and the catalyst, thereby prolonging the lifetime of PS and then stabilizing the photocatalytic system. To summarize, all the above experimental results confirmed that the photo-generated charge are transferred from PS to the catalyst in our photocatalytic system.

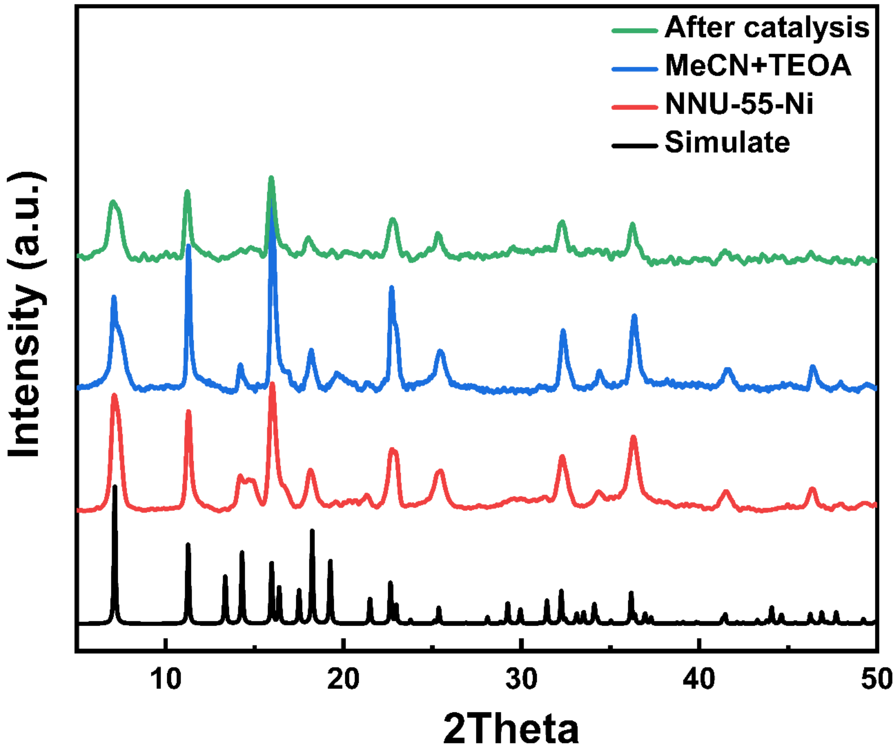

Supplementary Figure 36. The PXRD patterns of NNU-55-Ni under the same conditions of photocatalytic reaction solution (MeCN+TEOA) was immersed 7 days; the PXRD patterns of NNU-55-Ni before and after photocatalytic reaction.

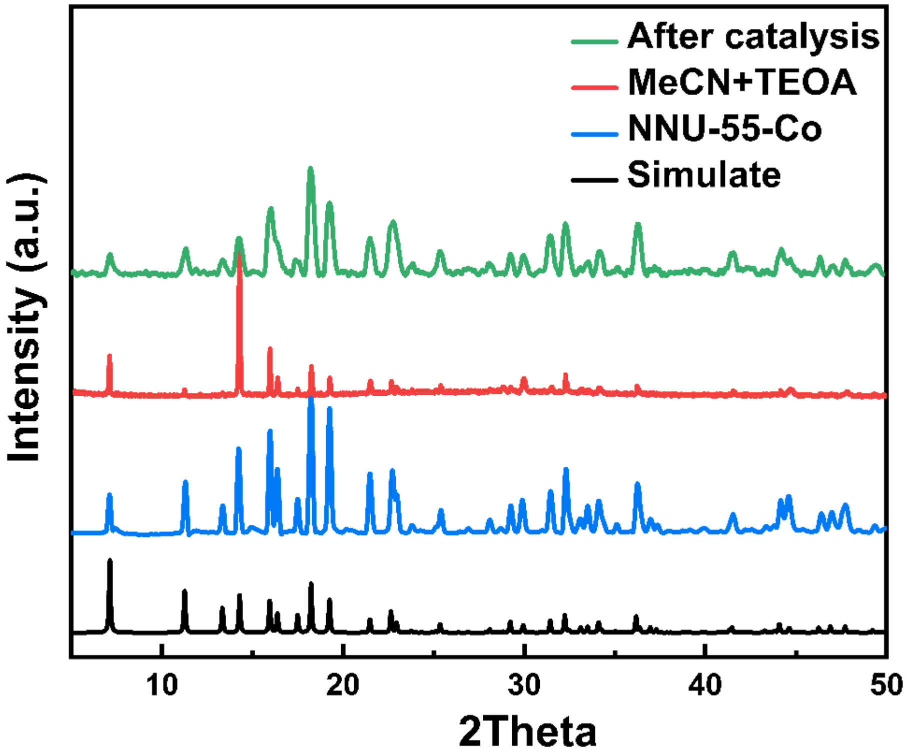

Supplementary Figure 37. The PXRD patterns of NNU-55-Co under the same conditions of photocatalytic reaction solution (MeCN+TEOA) was immersed 7 days; the PXRD patterns of NNU-55-Co before and after photocatalytic reaction.

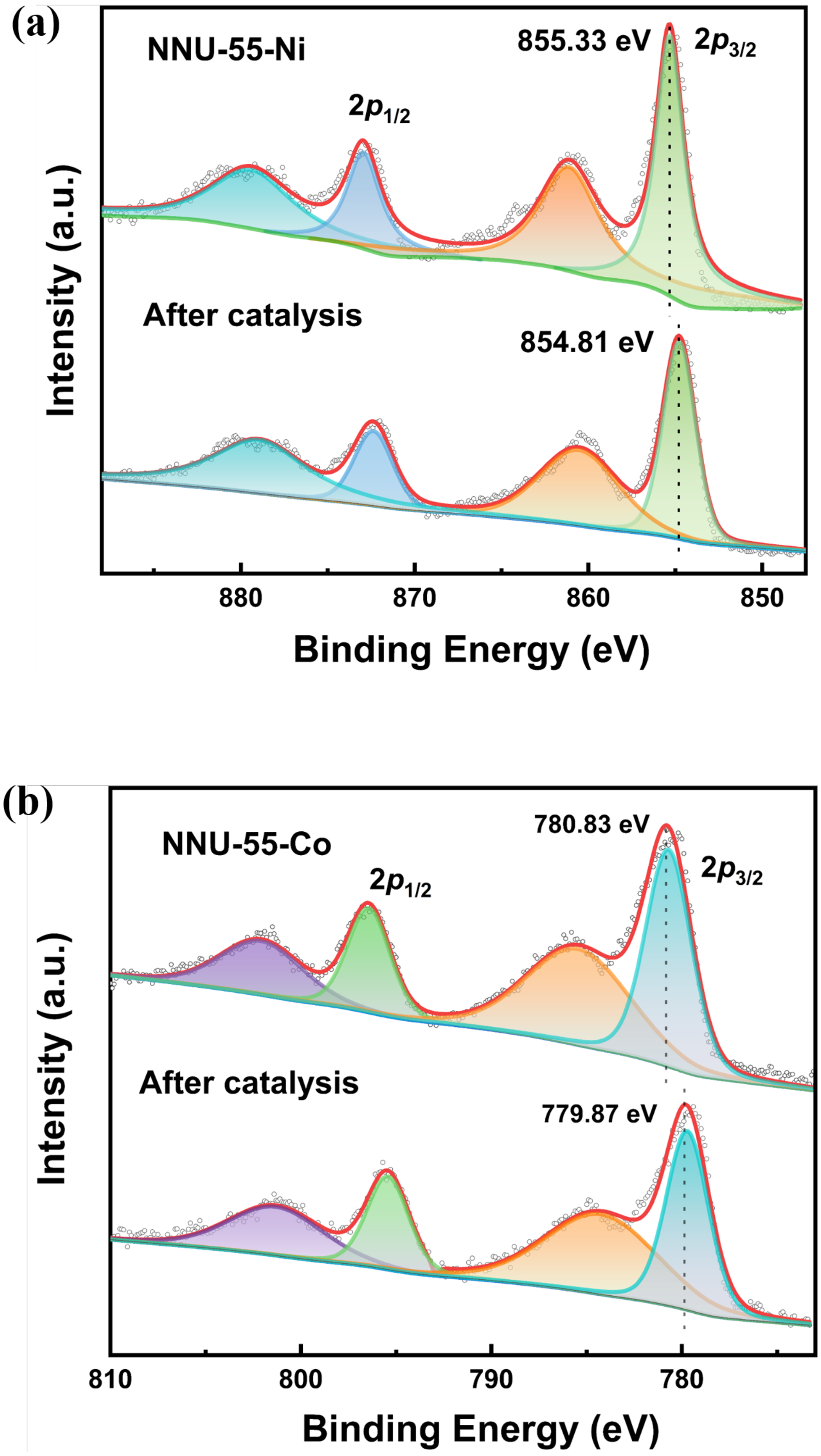

Supplementary Figure 38. The XPS spectra of (a). NNU-55-Ni and (b). NNU-55-Co before and after photocatalysis.

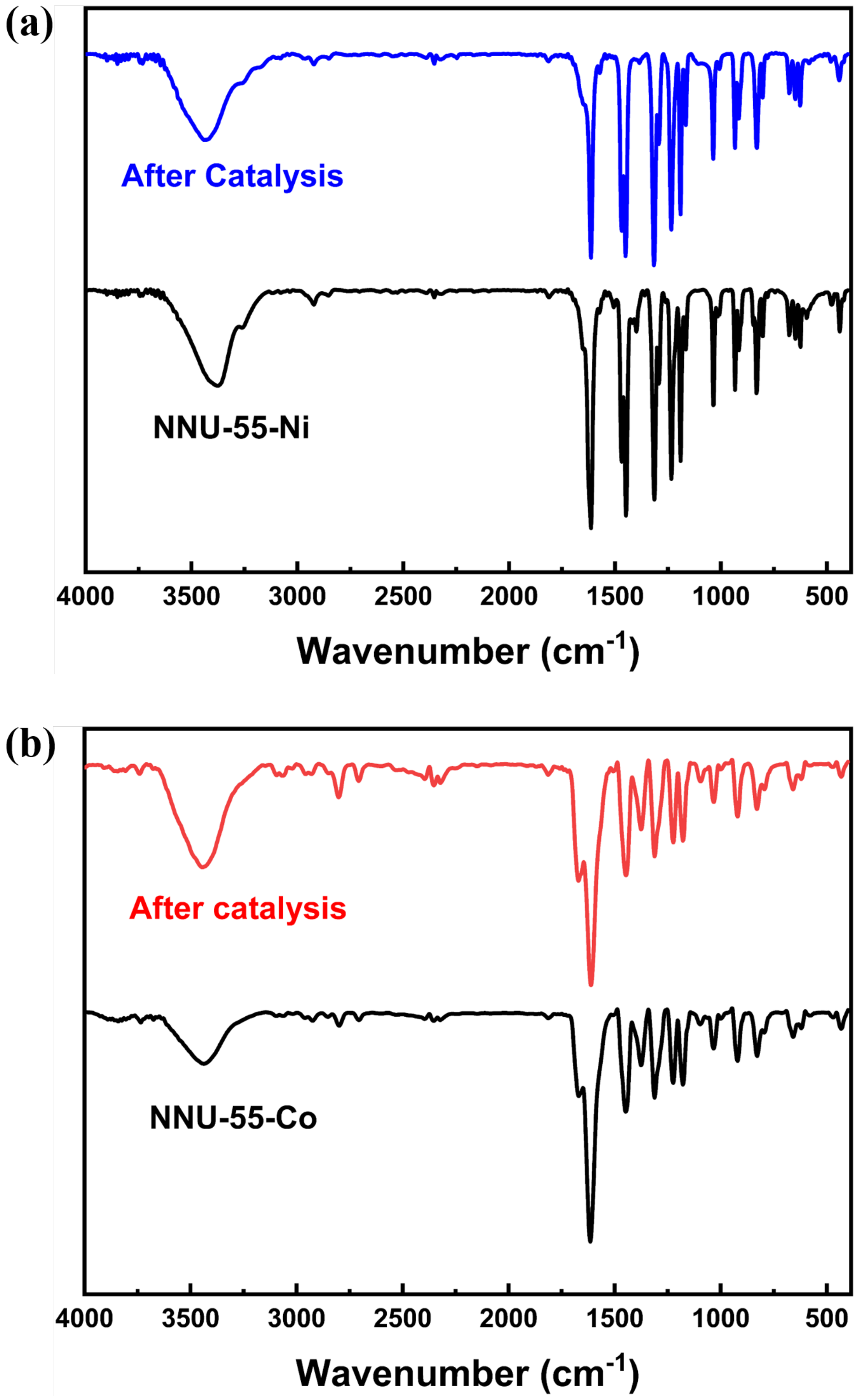

Supplementary Figure 39. FTIR spectra for (a). NNU-55-Ni and (b). NNU-55-Co before and after photocatalytic reaction test.

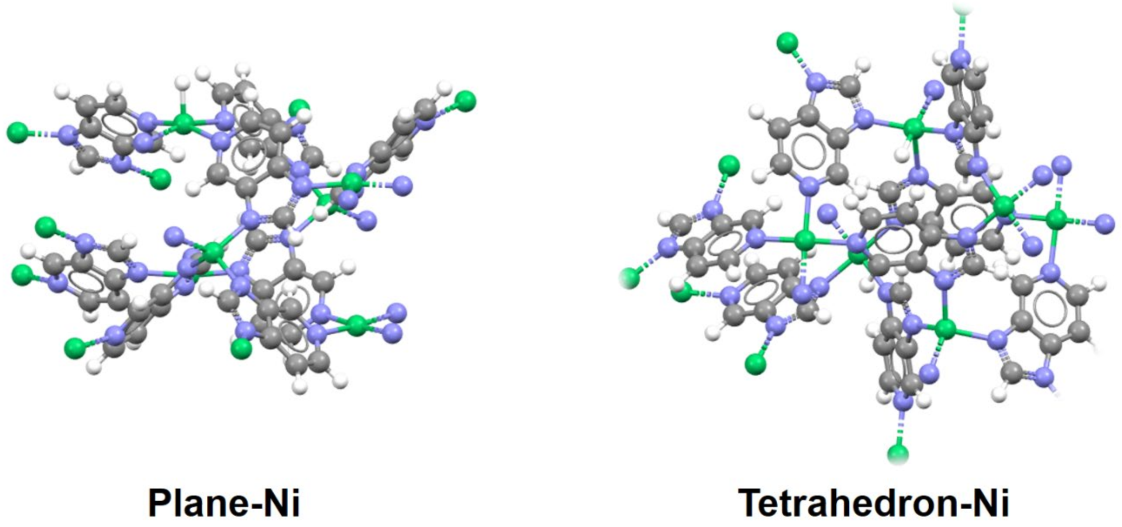

Supplementary Figure 40. The intermediate structures (*H) of HER on Plane -Ni and Tetrahedron-Ni.

(a) Plane-Ni:

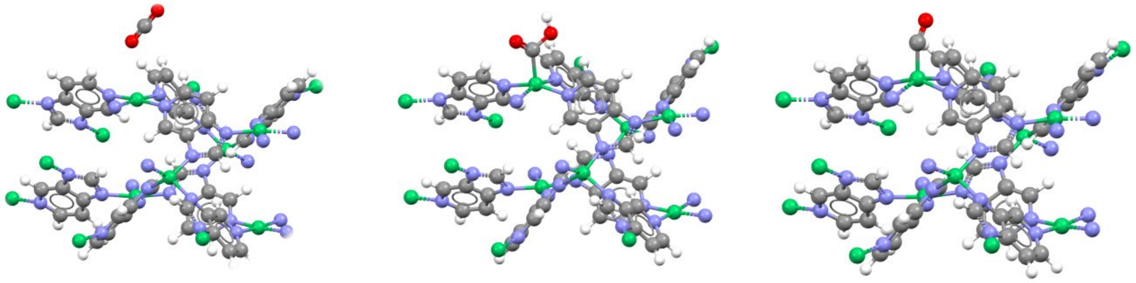

$^*CO_2$ $^*COOH$ $^*CO$

(b) Tetrahedron-Ni:

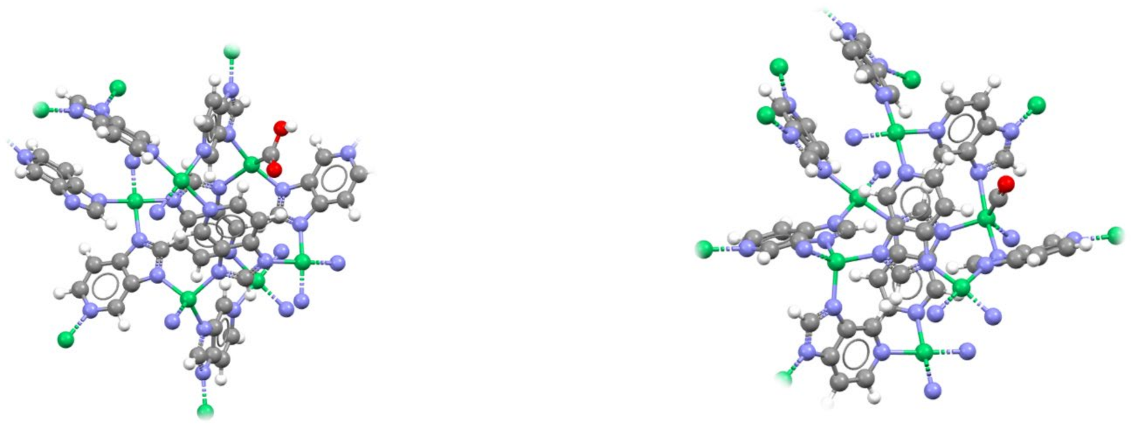

$^*COOH$ $^*CO$

Supplementary Figure 41. (a). The adsorption of *CO_2, *COOH, and *CO intermediates on Plan-Ni site. **(b).** The adsorption of *COOH, and *CO intermediates on Tetrahedron-Ni site.

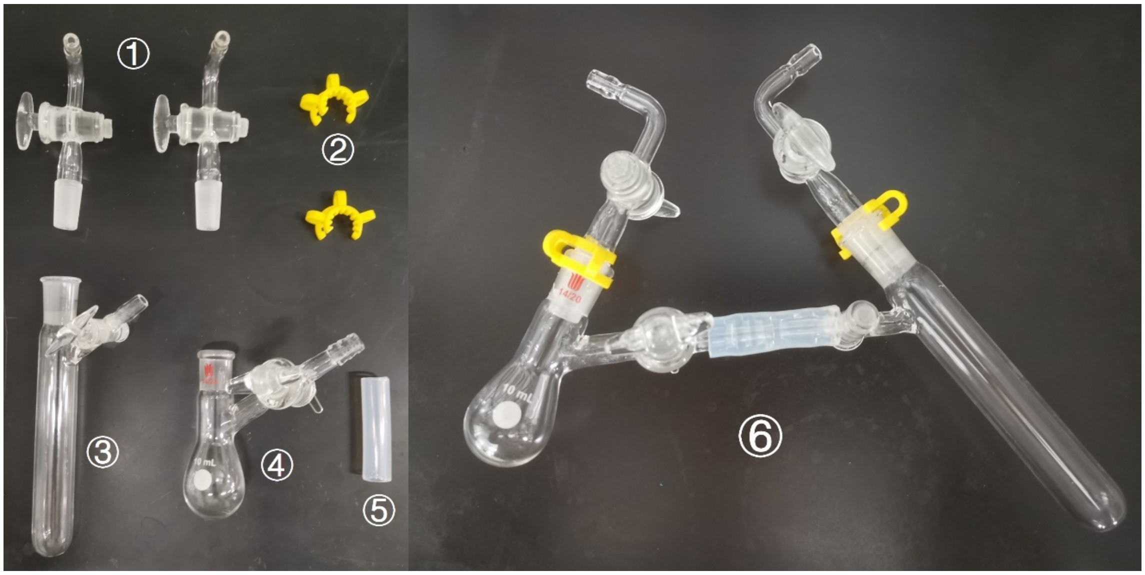

Supplementary Figure 42. ①: 90° connector with ground glass valves. ②: Cone interface clamp. ③: Schlenk tube for the photocatalytic reaction. ④: Schlenk type tube for the carbonylation reaction. ⑤: A tube for connection. ⑥: The final tandem reaction system. The photocatalytic CO_2RR on the right of the chamber (chamber A) and the carbonylation reaction on the left (chamber B).

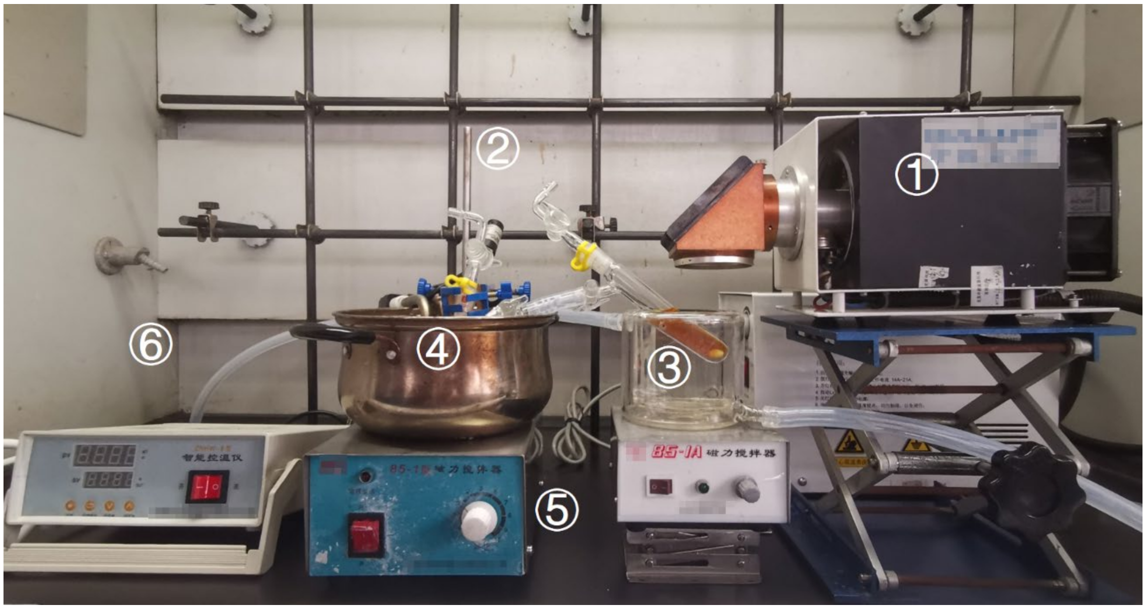

Switch On:

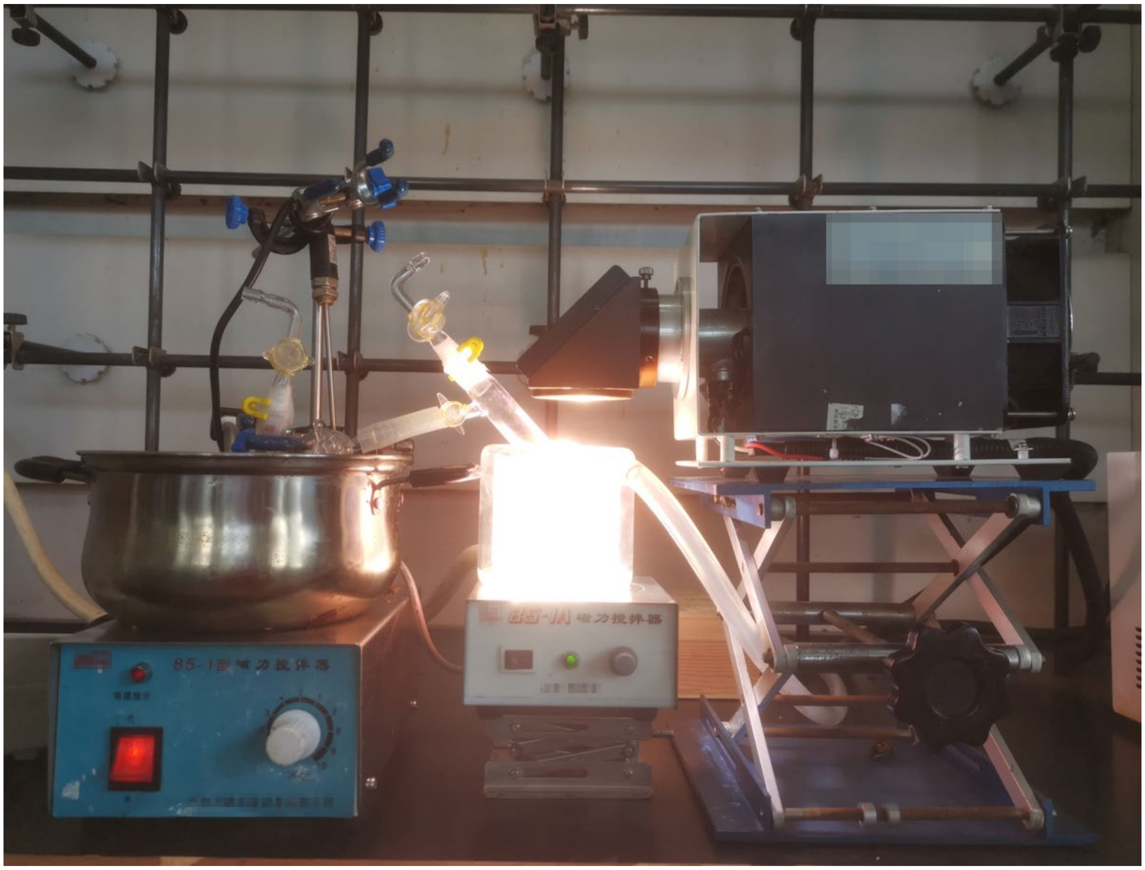

Supplementary Figure 43. ①: A 300 W Xenon light with an ultraviolet-cutoff filter (λ>= 420 nm). ②: The tandem reaction devices. The photocatalytic CO_2RR on the right of the chamber and the carbonylation reaction on the left. ③: The device of Condensed water. ④: The oil bath apparatus. ⑤: Magnetic stirrer. ⑥: The device of temperature-controlled.

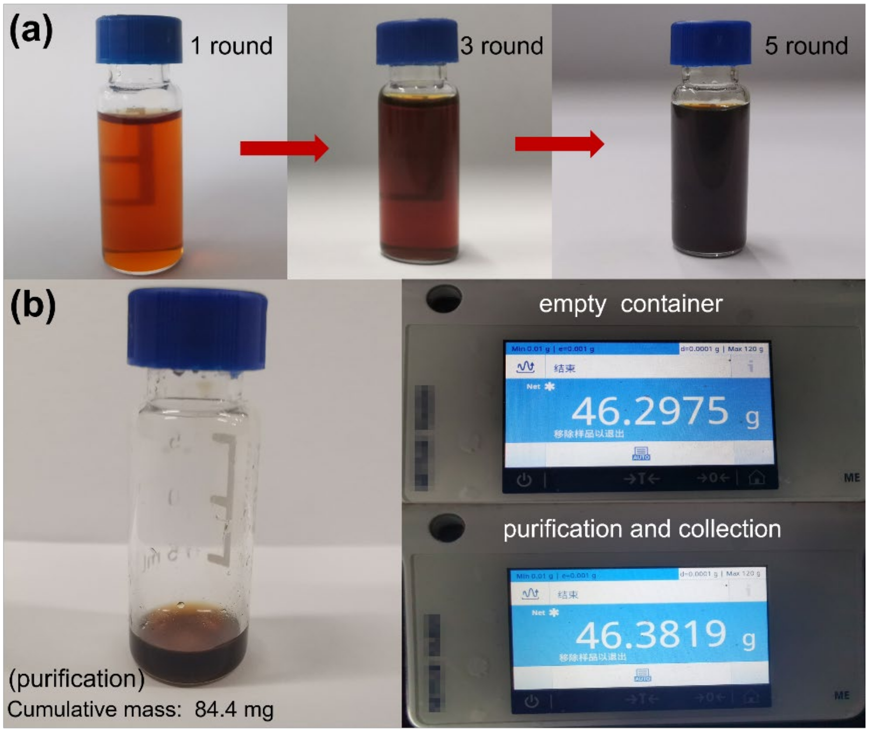

Supplementary Figure 44. (a). The carbonylation reaction gradually accumulates the amide product (DEET) and causes the color of reaction solution change with the different number of rounds of photocatalytic CO_2RR cycles. **(b).** Scale up the experiment to get the quality of purified DEET product.

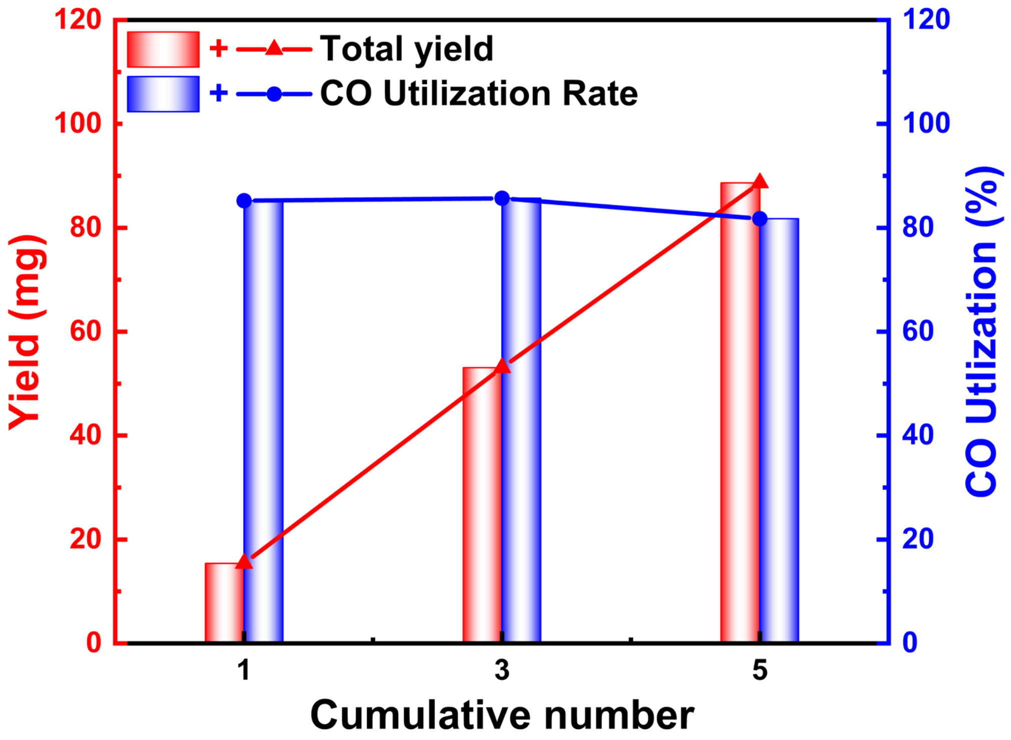

Supplementary Figure 45. The total yield and CO utilization rate of DEET in different cumulative rounds.

The GC-MS analysis results indicated that the photocatalytic product CO could maintain the initial conversion rate in the cycle experiments (within the range of 82% - 86%) (**Supplementary Figure 45**). After five rounds of the cycle experiments, the total product DEET was calculated to be 88.6 mg by using the standard curve method (**Supplementary Figure 45**). Moreover, the crude products of DEET were purified via silica column chromatography to obtain 84.4 mg of oily products (**Supplementary Figure 44b**).

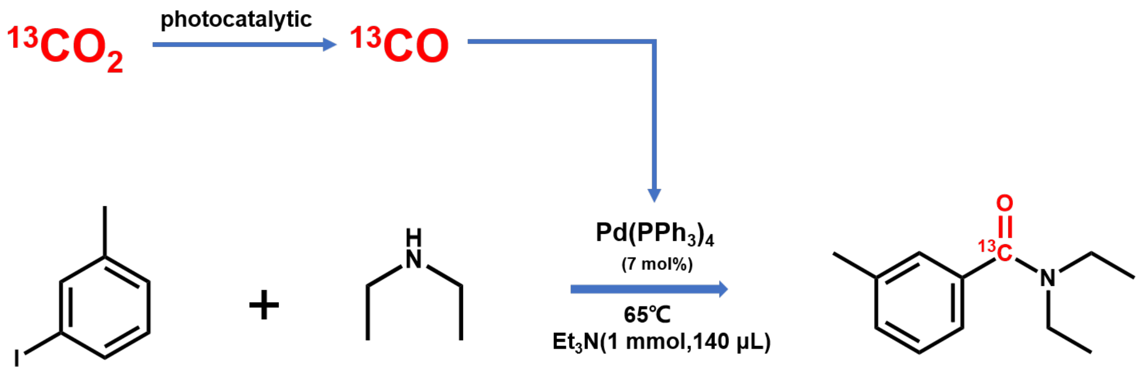

Supplementary Figure 46. Schematic of ^{13}C-Labeled DEET.

(a)

Method	DirectInfusion_TuneLow_pos.m	Operator	bruker
Sample Name	^{12}C	Instrument	micrOTOF-Q III 8228888.20519
Comment			

Acquisition Parameter

Source Type	ESI	Ion Polarity	Positive	Set Nebulizer	0.4 Bar
Focus	Active	Set Capillary	4500 V	Set Dry Heater	180 °C
Scan Begin	50 m/z	Set End Plate Offset	-500 V	Set Dry Gas	4.0 l/min
Scan End	1000 m/z	Set Collision Cell RF	140.0 Vpp	Set Divert Valve	Waste

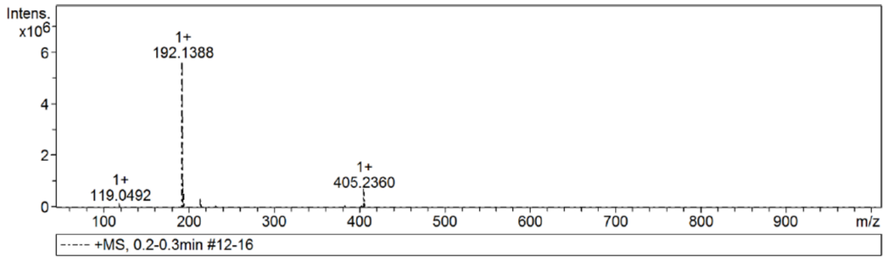

#	m/z	Res.	S/N	I	I %	FWHM
1	91.0494	7392	671.3	18579	0.3	0.0123
2	109.0627	12246	258.4	7288	0.1	0.0089
3	119.0492	8370	4259.4	122360	2.2	0.0142
4	120.0511	10152	384.0	11080	0.2	0.0118
5	192.1388	16943	169907.4	5623473	100.0	0.0113
6	193.1377	15966	28811.6	954666	17.0	0.0121
7	194.1374	12992	1519.7	50334	0.9	0.0149

HRMS-^{12}**C**-DEET (m / z): [M + H^+] calcd for $[C_{12}H_{17}NOH]^+$, 192.1383; found, 192.1388.

(b)

Method	DirectInfusion_TuneLow_pos.m	Operator	bruker
Sample Name	^{13}C	Instrument	micrOTOF-Q III 8228888.20519
Comment			

Acquisition Parameter

Source Type	ESI	Ion Polarity	Positive	Set Nebulizer	0.4 Bar
Focus	Active	Set Capillary	4500 V	Set Dry Heater	180 °C
Scan Begin	50 m/z	Set End Plate Offset	-500 V	Set Dry Gas	4.0 l/min
Scan End	1000 m/z	Set Collision Cell RF	140.0 Vpp	Set Divert Valve	Waste

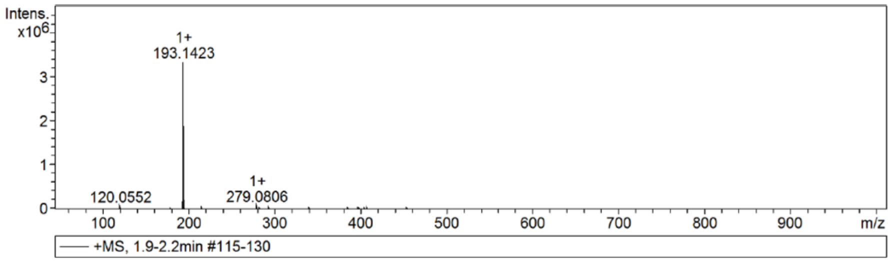

#	m/z	Res.	S/N	I	I %	FWHM
1	91.0517	11531	491.8	9421	0.3	0.0079
2	120.0552	11018	3126.0	64975	2.0	0.0109
3	179.1238	10124	1209.5	32099	1.0	0.0177
4	192.1368	10242	5959.4	166995	5.0	0.0188
5	193.1423	18050	118413.8	3328463	100.0	0.0107
6	194.1432	14285	12080.7	340871	10.2	0.0136
7	195.1425	9761	598.0	16942	0.5	0.0200

HRMS-^{13}**C**-DEET (m / z): [M + H^+] calcd for $[C_{11}{}^{13}CH_{17}NOH]^+$, 193.1416; found, 193.1423.

Supplementary Figure 47. High Resolution Mass Spectrometry (HRMS) of (a). ^{12}C-DEET and (b). ^{13}C-DEET.

Supplementary Tables

Supplementary Table 1. Crystal data and structure refinement for NNU-55-Co.

**Complexes**	**NNU-55-Co**
formula	$C_{28}H_{19}Co_3N_{12}O_6$
f*w*	796.34
crystal system	tetragonal
space group	*P*4/*n*
a (Å)	11.097(3)
b (Å)	11.097(3)
c (Å)	12.381(4)
α (deg)	90
β (deg)	90
γ (deg)	90
V (Å^3)	1524.6(10)
Z	2
D_{calcd} (g cm^{-3})	1.735
R(int)	0.0495
μ (mm^{-1})	1.678
F (000)	800.0
R_1 [$I > 2\sigma(I)$]^a	0.0349
wR_2 [$I > 2\sigma(I)$]^b	0.0841
R_1 (all data)	0.0504
wR_2 (all date)	0.0843
GOF on F^2	1.064

$^aR_1 = \sum||F_o| - |F_c||/|F_o|$. $^bwR_2 = [\sum w (F_o^2 - F_c^2)^2/\sum w(F_o^2)^2]^{1/2}$

Supplementary Table 2. The selected bond lengths (Å) for NNU-55-Co.

Co2	N2#1	2.162(3)	N3	C6	1.352(5)
Co2	N2#2	2.162(3)	N3	C5	1.347(5)
Co2	N2#3	2.162(3)	N2	C8	1.374(5)
Co2	N2	2.162(3)	N2	C3	1.342(5)
Co2	O5#4	2.052(8)	O5	Co2#4	2.052(8)
Co2	O2	2.039(7)	O5	C9	1.066(9)
Co1	N1#5	2.013(3)	C4	C8	1.406(5)
Co1	N1	2.013(3)	C4	C5	1.385(5)
Co1	N1#6	2.013(3)	C8	C7	1.394(5)
Co1	N1#7	2.013(3)	C7	C6	1.371(5)
Co3	N3#8	2.199(3)	O2	C10	1.103(5)
Co3	N3#9	2.199(3)	O3	Co3#11	2.021(7)
Co3	N3#10	2.199(3)	O3	C10	1.090(9)
Co3	N3	2.199(3)	C1	O1	1.647(18)
Co3	O3#11	2.021(7)	C1	C2#12	1.35(2)
Co3	O4	1.997(8)	C1	C2	1.35(2)
N1	C4	1.404(5)	C1	C2#13	1.35(2)
N1	C3	1.338(5)	C9	O4	1.012(9)

Supplementary Table 3. Kinetic analysis of emission decay for NNU-55-Co[a], NNU-55-Ni[b].

Samples	τ_1	A_1/%	τ_2	A_2/%	R^2	$\tau_{average}$/ns
1[a]	10	98.37	35	1.63	0.999	11
2[b]	9	99.84	50	0.16	0.999	9

The average lifetime was calculated by using the equation:

$$\tau_{average} = \frac{(A_1\tau_1^2 + A_2\tau_2^2)}{(A_1\tau_1 + A_2\tau_2)} \quad (1)$$

The time-resolved fluorescence decay spectrum was fitted using the E-exponential model:

$$y = y_0 + \sum A_n e^{\frac{-(x-x_0)}{\tau_n}} \; (n\text{=1,2,3......}) \quad (2)$$

Supplementary Table 4. Summary of state-of-art photocatalysts for reduction of CO_2 to CO.

Catalysts	Mass (mg)	Reaction medium	CO Total yield (μmol)	CO generation rate (μmol g^{-1} h^{-1})	Selectivity (%)	Ref.
**NNU-55-Ni**	**20**	**$Ru(bpy)_3^{2+}$ TEOA**	**85.3**	**266.6**	**81**	**This work**
**NNU-55-Ni-NS**	**20**	**$Ru(bpy)_3^{2+}$ TEOA**	**131.3**	**410.3**	**81**	**This work**
MOF-525	2	TEOA	0.8	64.0	91	5[11]
MOF-525-Zn	2	TEOA	1.3	111.7	90	5[11]
MOF-525-Co	2	TEOA	2.4	200.6	86	5[11]
MOF-Ni	5	$Ru(bpy)_3^{2+}$ TIPA	22.3	371.6	97	6[12]
MOF-Co	5	$Ru(bpy)_3^{2+}$ TIPA	22.8	1140.0	47	7[12]
ZrPP-1-Co	20	TEOA	4.14	14	96	7[13]
Co_6-MOF	5	$Ru(bpy)_3^{2+}$ TEOA	39.4	2624	58	8[14]
Ni-TpBpy	10	$Ru(bpy)_3^{2+}$ TEOA	40.6	811.4	96	9[15]
$[Ni(bpet)]^{2+}$	13	$Ru(bpy)_3^{2+}$ BIH	86	84.5	99	11[16]
BCN	50	$Co(bpy)_3^{2+}$ TEOA	9.3	93	76.2	12[17]
BIF-29	10	$Ru(bpy)_3^{2+}$ TEOA	167	3334	82.6	13[18]

Supplementary Table 5. The research of contrast conditions for NNU-55-Ni.

entry	Catalyst	CO (μmol)
1	**NNU-55-Ni**	n.d.
2	**NNU-55-Ni**	n.d.
3	$NiCl_2$	11.0
4	5-AD	3.6
5	$NiCl_2$ + 5-AD	12.3
6	None	trace
7	None PS	n.d.
8	None SD	n.d.

Reaction conditions: PS = $[Ru(bpy)_3]Cl_2 \cdot 6H_2O$ (10 mg), SD = TEOA (4 mL), CH_3CN+H_2O (13 mL + 100 μL), CO_2 (1 atm), λ ≥420 nm, 25°C, 16h, n.d.=Not detectable. **1**. in the dark; **2**. in the N_2; **3**. with 20 mg $NiCl_2$; **4**. with 20 mg 5-AD; **5**. with 20 mg $NiCl_2$ and 20 mg 5-AD; **6.** without any catalyst; **7**. without photosensitizers; **8**. without sacrificial agent.

Supplementary Table 6. Kinetic analysis of emission decay for 0.04 mM $[Ru(bpy)_3]Cl_2$[b], Ru/Bulky NNU-55-Ni [c], and Ru/NNU-55-Ni-NS [d].

Samples	τ_1	A_1/%	τ_2	A_2/%	R^2	$\tau_{average}$/ns[a]
1[b]	211	100.0	-	-	0.999	211
2[c]	192	100.0	-	-	0.999	192
3[d]	183	100.0	-	-	0.999	183

[a]. The average lifetime.

[c] and **[d]** with 1 mg catalyst.

Supplementary Table 7. The Conversion rate and Isolated yield to the final product in the tandem reaction.

Compounds[a]	CO Conversion rate (%)	Mass of isolated product (mg)
1	85	15.4
2	40	7.6
3	49	9.7
4	70	10.6
5	72	12.4
6	67	8.2
7	78	16.2
8	58	9.9
9	51	10.4
10	74	18.9
11	77	11.4
12	59	12.1
13	44	7.7

a: The compound serial number corresponds to the serial number of Supplementary Notes.

Supplementary Notes

Supplementary Note 1: Materials and General Methods.

All chemicals and solvents were commercially available and used without further purification. The anhydrous solvents were recommended for organic reaction. Powder X-ray diffraction (PXRD) pattern measurements were collected on a Rigaku SmartLab diffractometer equipped with graphite monochromatized Cu Kα radiation (λ= 1.54060 Å) at 298K. Infrared spectra (IR) were measured on a NEXUS670. Thermogravimetric analyses (TGA) were carried out on a Diamond DSC Pyris analyzer (Perkin-Elmer). 273K and 298K CO_2 adsorption-desorption isotherms were determined by Autosorb IQ2 (Quantachrome Instruments). The electrochemical test was carried out with EC-Lab SP-150 workstation (Bio-Logic) and CHI 660E (CH Instruments). X-ray photoelectron spectroscopy (XPS) was recorded using an Escalab 250Xi instrument (Thermo Scientific) equipped with an Al Kα microfocused X-ray source. UV-Vis-NIR diffuse reflectance spectra (UV-Vis-NIR DRS) was performed on a Varian Cary 5000. Gas chromatography was performed on GC-7890A (Aulight Co.,) equiped with a flame ionization detector (FID) with a methanizer and a thermal conductivity detector (TCD). Gas chromatography-mass spectrometer (GC-MS) was performed on 7890B and 5977B (Agilent Technologies) equiped HP-5-MSUI or HP-PLOT Molesieve capillary column. 1H NMR and 13C NMR were carried out AVANCE III 400M spectrometer (Bruker). Chemical shifts (δ) are reported in parts per million (ppm) relative to incompletely deuterated $CDCl_3$ (s, 7.26 ppm). Splitting patterns are designated as s, singlet; d, doublet; t, triplet; q, quartet; p, pentet; dd, doublet of doublets; td, triplet of doublets; m, multiplet.

Supplementary Note 2: Single-Crystal X-ray Analyses.

The single-crystal diffraction analysis of compound NNU-55-Co was collected on Bruker APEX Duo II equipment CCD area detector at 296 K. The X-ray generator was operated at 50 kV and 35 mA using Mo Kα (λ = 0.71073 Å) radiation. Data integration was performed using SAINT. Routine Lorentz and polarization corrections were applied. Multiscan absorption corrections were performed using SADABS. Those structures were solved with the ShelXT[1] structure solution program using Intrinsic Phasing and refined with the ShelXL-2018[2] refinement package using Least Squares minimization on Olex-2[3]

software. All the solvent molecules which are highly disordered and not able to be modeled were treated by the SQUEEZE routine in PLATON. The topological analyses were performed with TOPOS[4]. The detailed structure determination parameters and crystallographic data are shown in Table S1.

Supplementary Note 3: Calculational Methods.

The calculations were performed using the ORCA package employing the resolution of identity approximation[5]. All the DFT calculations were performed using the hybrid B3LYP functional. Basis sets of def2-TZVP[6] were adopted for all atoms in the complexes with decontracted auxiliary def2-TZVP/J Coulomb fitting basis sets[7]. D3 dispersion correction developed by Grimme is included for weak interactions[8].

The computational hydrogen electrode (CHE) model that proposed by Nørskov et al[9]. was applied to describe the Gibbs reaction free energy of reaction for CO_2RR elementary steps involving ($H^+ + e^-$) pair transfer. The calculation for each step can be defined as $\Delta G_n(U) = \Delta G_n(U=0) + neU$, where e is the elementary charge of an electron, n is the number of ($H^+ + e^-$) pairs transferred in CO_2RR and U is the electrode potential versus the reversible hydrogen electrode (RHE). Specifically, $\Delta G = \Delta E + \Delta E_{ZPE} - T\Delta S + \int C_P dT$ at U=0 V, where ΔE is the reaction energy difference between the product and reactant of the CO_2RR occurring on catalysts, which can be directly obtained from DFT computations; ΔE_{ZPE}, $T\Delta S$ and $\int Cp dT$ are zero-point energy correction, entropy correction and enthalpic temperature correction at T=298.15 K respectively, which were calculated from the vibrational frequencies. The ΔE_{ZPE}, $T\Delta S$ and $\int Cp dT$ for each reaction intermediates can be calculated by the following equations, respectively:

$$E_{ZPE} = \frac{1}{2}\sum_i hv_i \qquad (1)$$

$$-TS = K_B T \sum_i ln(1 - e^{-\frac{hv_i}{K_B T}}) - \sum_i hv_i(\frac{1}{e^{\frac{hv_i}{K_B T}} - 1}) \qquad (2)$$

$$\int C_p \, dT = \sum_i hv_i(\frac{1}{e^{\frac{hv_i}{K_B T}} - 1}) \qquad (3)$$

where h, v and K_B are Planck constant, vibrational frequencies and Boltzmann constant, respectively.

Supplementary Note 4: Electrochemical Measurements.

Preparation of the working electrode.

The as-synthesized 2 mg photocatalyst (NNU-55-M) were dispersed in a mixed solution of 1 ml ethanol and 10 μL Nafion D-520 dispersion solutions. The mixture was sonicated for a while to generate a homogeneous suspension. Subsequently, 200 μL of suspensions was transferred and uniformly covers on ITO glass plates (1 cm × 2 cm) via a simple drop-coating, then dried at room temperature.

Mott-Schottky plot measurements.

The Mott-Schottky plots were measured over an alternating current (AC) frequency of 500 Hz, 1,000 Hz and 1,500 Hz. The experiments were performed with an SP-150 electrochemical workstation via a conventional three-electrode system in a 0.5 M Na_2SO_4 aqueous solution (pH = 6.8). The Ag / AgCl electrode was employed as the reference electrode, and the platinum plate was used as the counter electrode, respectively.

Photocurrent measurements.

Photoelectrochemical measurements were similar to Mott-Schottky measurement with CHI 660e electrochemical workstation in a standard three-electrode electrochemical cell with a working electrode, a platinum plate as counter electrode and a saturated Ag/AgCl electrode as reference electrode. The detailed preparation of working electrodes was in line with the above experiments. A Xenon light with an ultraviolet-cutoff filter (λ>= 420 nm) was applied as the light source for Photocurrent, and 0.5 M Na_2SO_4 aqueous solution was used as the electrolyte. During the photoelectrochemical measurements, the distance between the light source and three-electrode electrochemical cell was about 20 cm, and a 5.0 mV bias potential was applied for the testing of photocurrent–time (I-t) curves. When the background current was stabilized, the working electrode was illuminated for 20s (light on) and then the light source was shielded for 20s (light off), repeated the above operation and recorded the photocurrent-time (I-t) curves through the electrochemical workstation, and finally obtained the photocurrent curve.

Supplementary Note 5: XAFS Characterization.

The X-ray absorption fine structure spectra (XAFS) Ni K-edge were collected at BL07A1 beamline of National Synchrotron Radiation Reaearch Center (NSRRC). The data were collected in fluorescence mode using a Lytle detector while the corresponding reference sample were collected in transmission mode. The sample were grinded and uniformly daubed on the special adhesive tape.

EXAFS fitting parameters: The obtained XAFS data was processed in Athena (version 0.9.26) for background, pre-edge line and post-edge line calibrations. Then Fourier transformed fitting was carried out in Artemis (version 0.9.26). The k^3 weighting, k-range of 2.5 ~ 10.5 $Å^{-1}$ and R range of 1 ~ 3 Å were used for the fitting of Ni foil; k-range of 3 ~ 13 $Å^{-1}$ and R range of 1 ~ 3 Å were used for the fitting of Ni samples. The four parameters, coordination number, bond length, Debye-Waller factor and E_0 shift (CN, R, ΔE_0) were fitted without anyone was fixed, the σ^2 was set.

Wavelet analysis parameters: For Wavelet Transform analysis, the χ(k) exported from Athena was imported into the Hama Fortran code. The parameters were listed as follow: R range, 1 ~ 4 Å, k range, 0 ~ 11 $Å^{-1}$ for Fe foil and 0 ~ 11 $Å^{-1}$ for Fe samples; k weight, 3; and Morlet function with κ=10, σ=1 was used as the mother wavelet to provide the overall distribution.

Supplementary Note 6: Apparent Quantum Efficiency (AQE).

The AQE was calculated as follow:

$$AQE = \frac{(2 \times \textit{the number of product}) \textit{ molecules produces}}{\textit{the number of incident photons}} \times 100\% \quad (1)$$

$$AQE = \frac{2 \times M \times N_A \times h \times c}{S \times P \times T \times \lambda} \times 100\% \quad (2)$$

M = yield of product (mol);

N_A (Avogadro constant) = 6.02 × 10^{23} mol^{-1};

h (Planck constant) = 6.626 × 10^{-34} J·s;

c (Speed of light) = 3 × 10^8 m s^{-1};

S = Irradiation area (cm^2) = 9.42 cm^2;

P = the intensity of irradiation light (W / cm^2) = 35.4 × 10^{-3} W cm^{-2} (400 nm) or 39.4 × 10-

^{3} W cm^{-2} (450 nm) or 47.1 × 10^{-3} W cm^{-2} (500 nm) or 45.4 × 10^{-3} W cm^{-2} (530 nm);
T = the photoreaction time (s) = 16 × 3600 = 57600 s;
λ = the wavelength of the monochromatic light (nm) = 400 × 10^{-9} m / 450 × 10^{-9} m / 500 × 10^{-9} m / 530 × 10^{-9} m.
AQE (400 nm) = 0.12%, AQE (450 nm) = 0.19%, AQE (500 nm) = 0.06%, AQE (530 nm) = 0.01%.

Supplementary Note 7: Photophysical experiments.

Photoluminescent (PL) quenching experiments.

The excitation wavelength was 400 nm when steady-state PL was measured using Fluoromax-4 spectrofluorometer. All of solution systems were bubbled with CO_2 for 30 min to dissolve oxygen and maintain saturated conditions. To probe the fluorescence quenching of $[Ru(bpy)_3]Cl_2·6H_2O$ by NNU-55-Ni-NS catalysts, the photoluminescent quenching measurements were performed in MeCN / H_2O (100:1, 10 mL) mixed solution containing 0.04 mM $[Ru(bpy)_3]Cl_2·6H_2O$. The steady-state PL spectra of mixed solution containing $[Ru(bpy)_3]Cl_2·6H_2O$ were performed upon the addition of increasing amounts of NNU-55-Ni-NS (0, 0.5, 1.0, and 2.0 mg). To probe the fluorescence quenching of $[Ru(bpy)_3]Cl_2·6H_2O$ by TEOA, we added a small amount of TEOA (40.0 - 120.0 mM) to maintain the viscosity and concentration of $Ru(bpy)_3^{2+}$ mixture solutions (0.04 mM). Fluorescence intensity was then evaluated.

Photoluminescence lifetime experiments.

The samples were excited by the incident light of 390 nm and the PL decay spectra at 605 nm are monitored using PICOQuant FT-300 spectrofluorometer. These solution systems were bubbled with CO_2 for 30 min to dissolve oxygen and maintain saturated conditions. The mixed solution contained acetonitrile (10 mL) and deionized water (100 μL). Then $[Ru(bpy)_3]Cl_2·6H_2O$ (0.04 mM) was dissolved in 10 mL of the abovementioned mixed solution. Finally, 1 mg catalyst was added and the solution underwent ultrasonication to disperse the catalyst uniformly. Photoluminescence lifetimes were tested subsequently.

Supplementary Note 8: CO conversion rate.

CO conversions of all substrates were determined via gas chromatography (GC) and gas chromatography-mass spectrometry (GC-MS) analysis, concentrations were calculated based on a standard cur. First, photocatalytic reaction products were quantified via GC to obtain CO yield ($n_{CO\text{-}total}$ / μmol), the initial volumes of reaction solutions (V / mL). The concentrations of the amide compounds in each sample were determined using GC-MS (C_{amide} / mg mL^{-1}).

$$m_{amide} = C_{amide} \times V \qquad (1)$$

$$n_{amide} = \frac{m_{amide}}{M_{amide}} = n_{CO\text{-}amide} \qquad (2)$$

$$\text{CO conversion rate (\%)} = \frac{n_{CO\text{-}amide}}{n_{CO\text{-}total}} \qquad (3)$$

The yield of these isolated products was also isolated by silica gel chromatography.

Mass spectrum and standard curve of all products.

Supplementary Notes 9. N,N-diethyl-*m*-toluamide **(DEET)**[19]

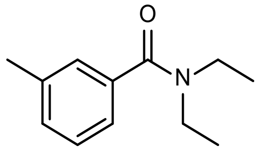

$C_{12}H_{17}NO$

M = 191.13 g mol^{-1}

^{1}H NMR: (400 MHz, Chloroform-*d*) δ 7.26–7.22 (m, 1H), 7.18–7.15 (m, 2H), 7.12 (dd, *J* = 8.0, 1.6 Hz, 1H), 3.55-3.49 (m, 2H), 3.25-3.20 (m, 2H), 2.34 (s, 3H), 1.22 (t, *J* = 8.0 Hz, 3H), 1.08 (t, *J* = 4.0 Hz, 3H).

^{13}C NMR: (101 MHz, Chloroform-*d*) δ 171.3, 138.1, 137.1, 129.6, 128.1, 126.8, 123.0, 43.1, 39.0, 21.3, 14.1, 12.8.

Mass spectral data:

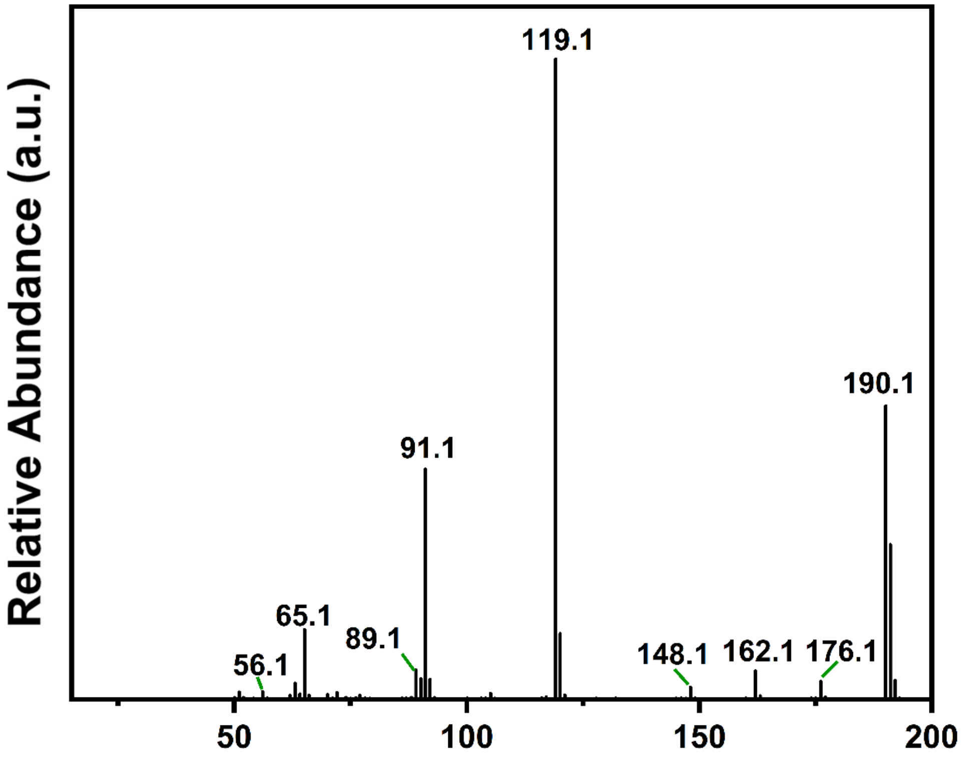

Standard Curve of DEET (GC-MS):

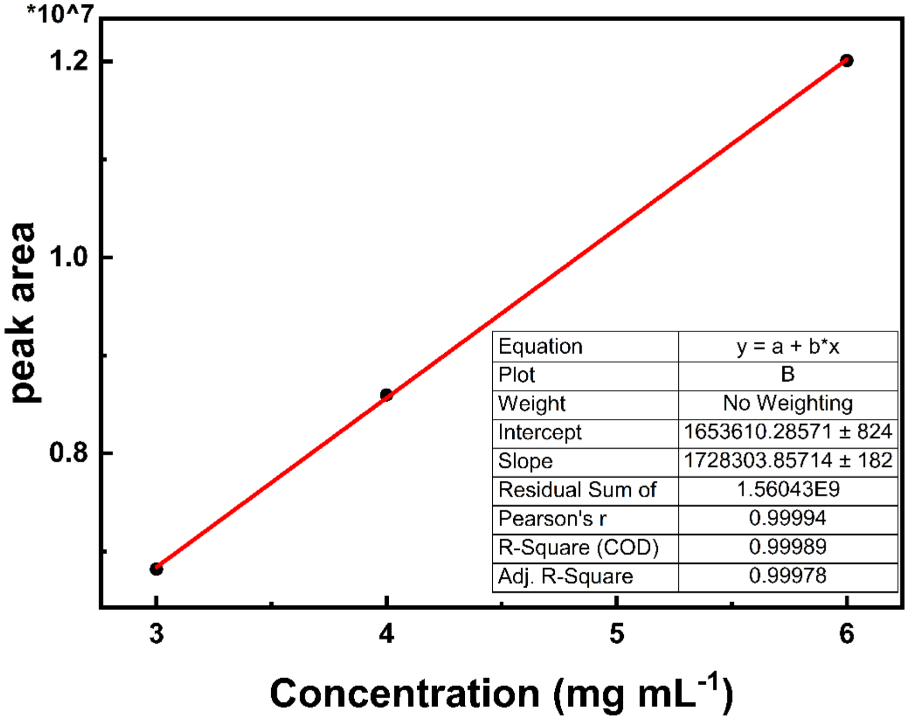

Supplementary Notes 10. N-phenylbenzamide[21]

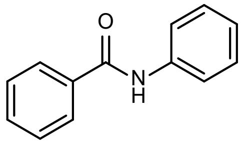

$C_{13}H_{11}NO$

M = 197.24 g mol^{-1}

1H NMR: (400 MHz, Chloroform-d) δ 7.92 (br, 1H), 7.88–7.85 (m, 2H), 7.66–7.63 (m, 2H), 7.57–7.53 (m, 1H), 7.45-7.50 (m, 2H), 7.39–7.34 (m, 2H), 7.18–7.13 (m, 1H).

13C NMR: (101 MHz, CDCl3) δ 165.8, 137.9, 134.9, 131.8, 129.1, 128.8, 127.0, 124.6, 120.2.

Mass spectral data:

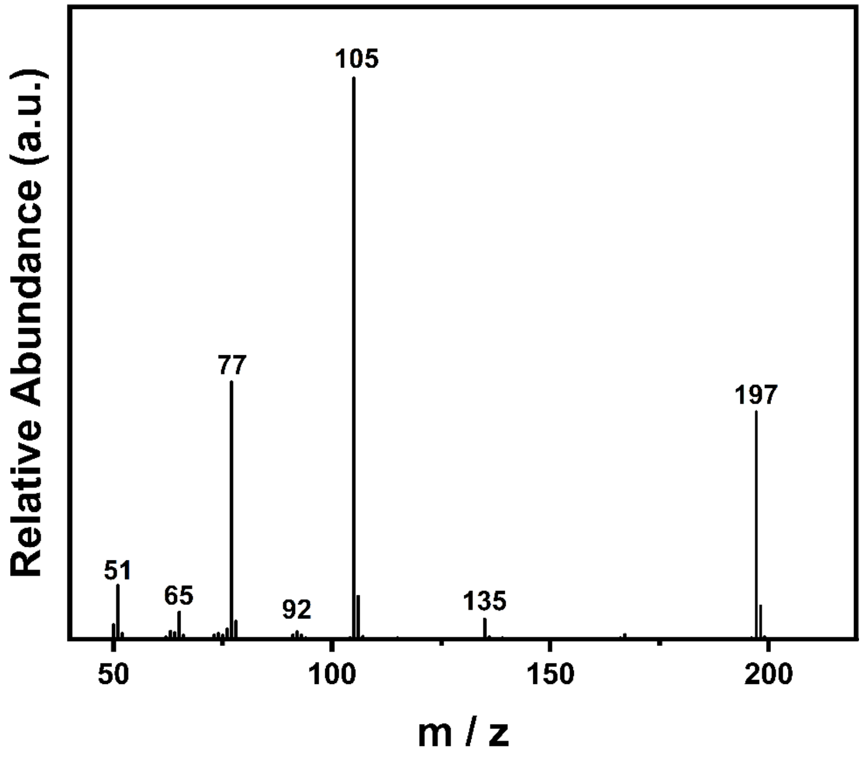

Standard Curve of compound (GC-MS):

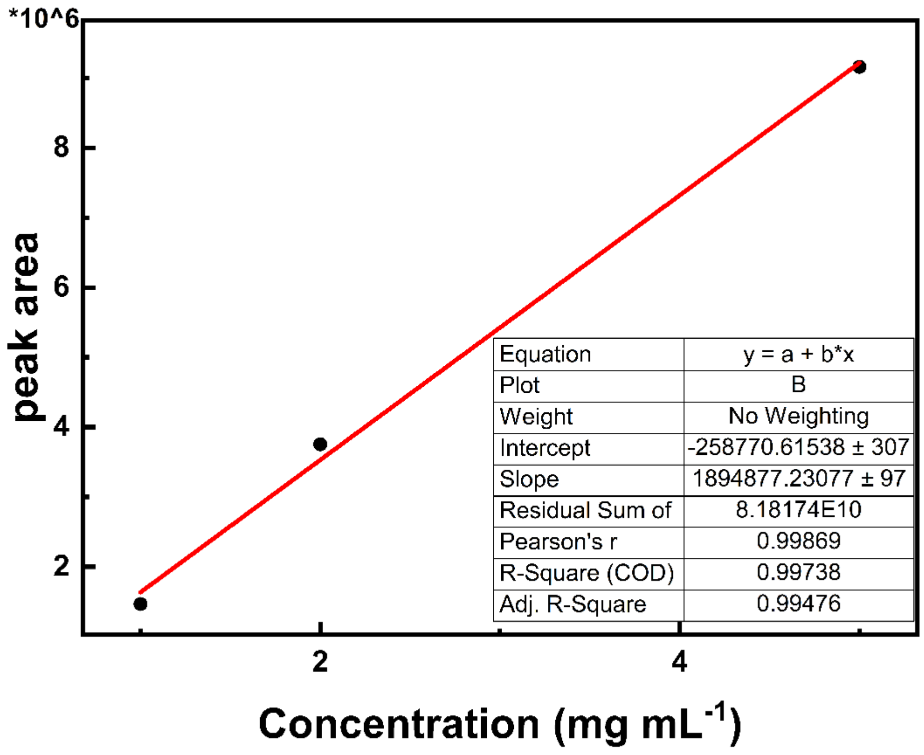

Supplementary Notes 11. N-benzylbenzamide[23]

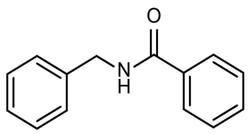

$C_{14}H_{13}NO$
M = 211.26 g mol^{-1}

^{1}H NMR: (400 MHz, Chloroform-*d*) δ 7.81–7.78 (m, 2H), 7.51–7.45 (m, 1H), 7.42–7.37 (m, 2H), 7.36 – 7.25 (m, 5H), 6.79 (s, 1H), 4.60 (d, *J* = 4.0 Hz, 2H).

^{13}C NMR: (101 MHz, Chloroform-*d*) δ 167.4, 138.1, 134.2, 131.4, 128.6, 128.5, 127.8, 127.4, 126.9, 43.9.

Mass spectral data:

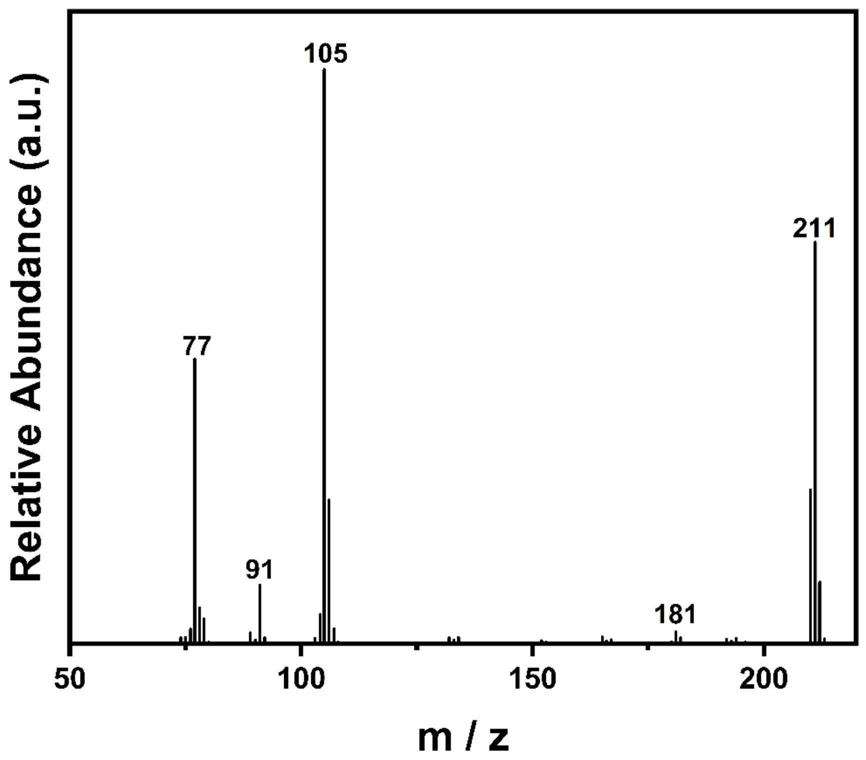

Standard Curve of compound (GC-MS):

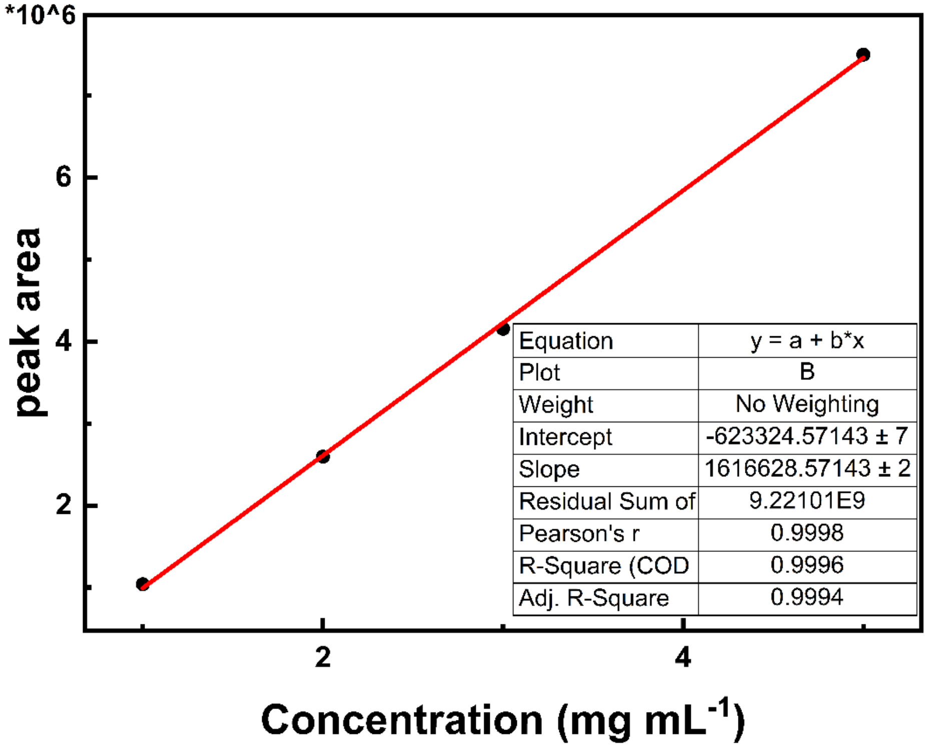

Supplementary Notes 12. N-butylbenzamide[21]

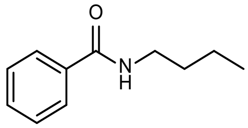

$C_{11}H_{15}NO$
M = 177.25 g mol^{-1}

^{1}H NMR (400 MHz, Chloroform-*d*): δ 7.77–7.75 (m, 2H), 7.44–7.40 (m, 1H), 7.34 (m, *J* = 12.0, 4.0 Hz, 2H), 6.85 (d, *J* = 8.0 Hz, 1H), 3.40–3.34 (m, 2H), 1.57–1.50 (m, 2H), 1.38–1.28 (m, 2H), 0.88 (t, *J* = 8.0 Hz, 3H).

^{13}C NMR (101 MHz, Chloroform-*d*): δ 167.6, 134.6, 131.0, 128.2, 126.8, 39.7, 31.5, 20.0, 13.6.

Mass spectral data:

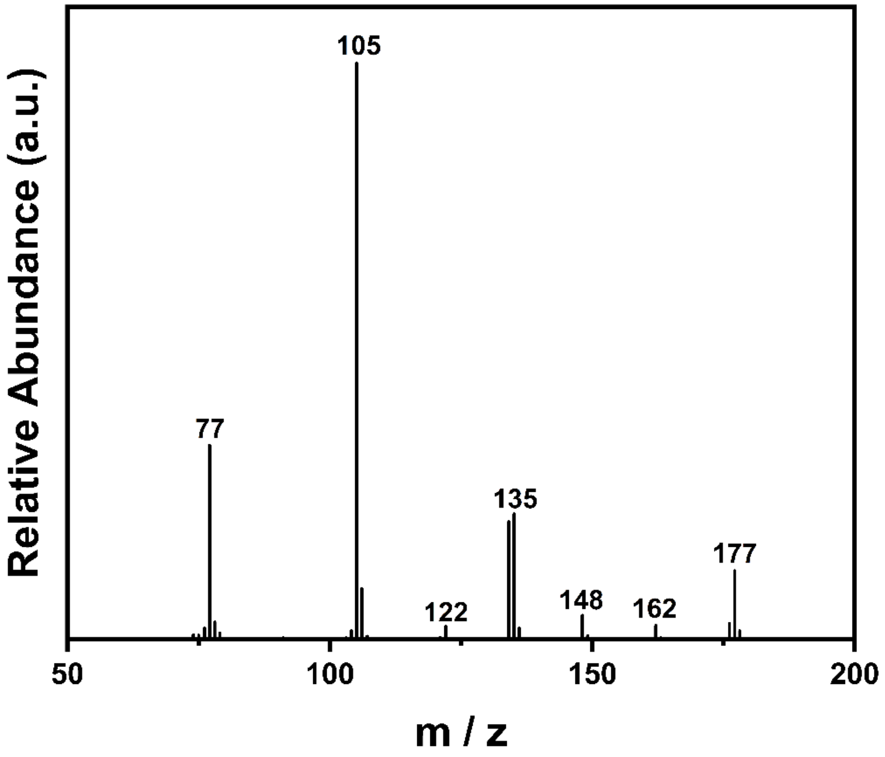

Standard Curve of compound (GC-MS):

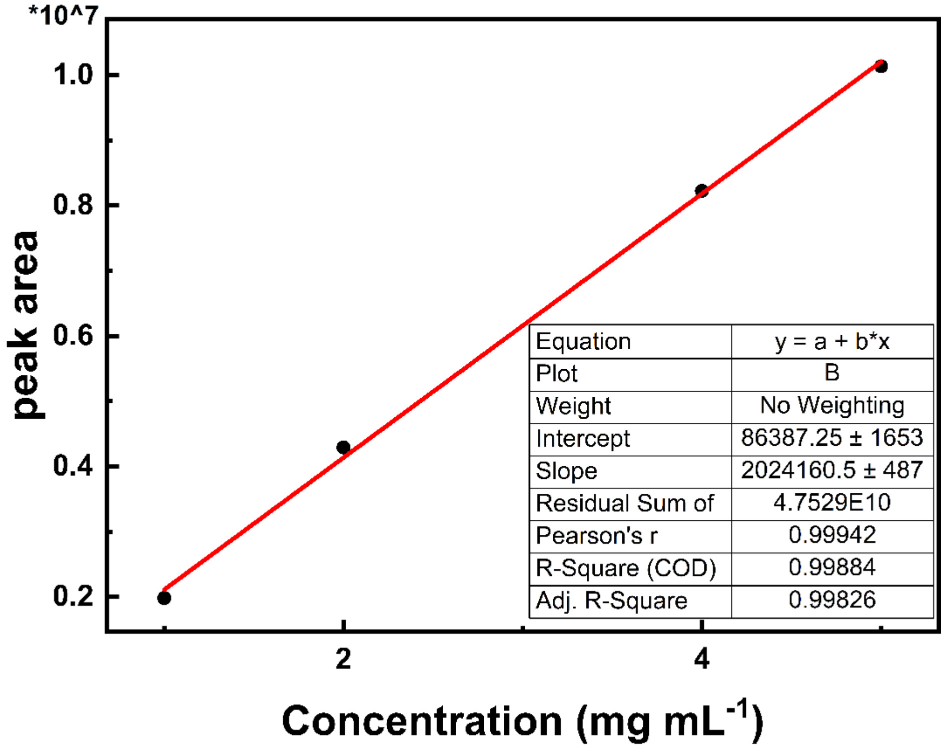

Supplementary Notes 13. N-butyl-4-methoxybenzamide[21]

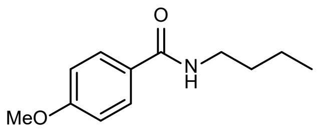

$C_{12}H_{17}NO_2$
M = 207.13 g mol^{-1}

^{1}H NMR: (400 MHz, Chloroform-*d*) δ 7.73 (d, *J* = 8.0 Hz, 2H), 6.83 (d, *J* = 8.0 Hz, 2H), 6.64 (t, *J* = 8.0 Hz, 1H), 3.77 (s, 3H), 3.38-3.33 (m, 2H), 1.56–1.49 (m, 2H), 1.37–1.28 (m, 2H), 0.88 (t, *J* = 8.0 Hz, 3H).

^{13}C NMR: (101 MHz, Chloroform-*d*) δ 167.0, 161.8, 128.6, 127.0, 113.4, 55.2, 39.6, 31.6, 20.0, 13.7.

Mass spectral data:

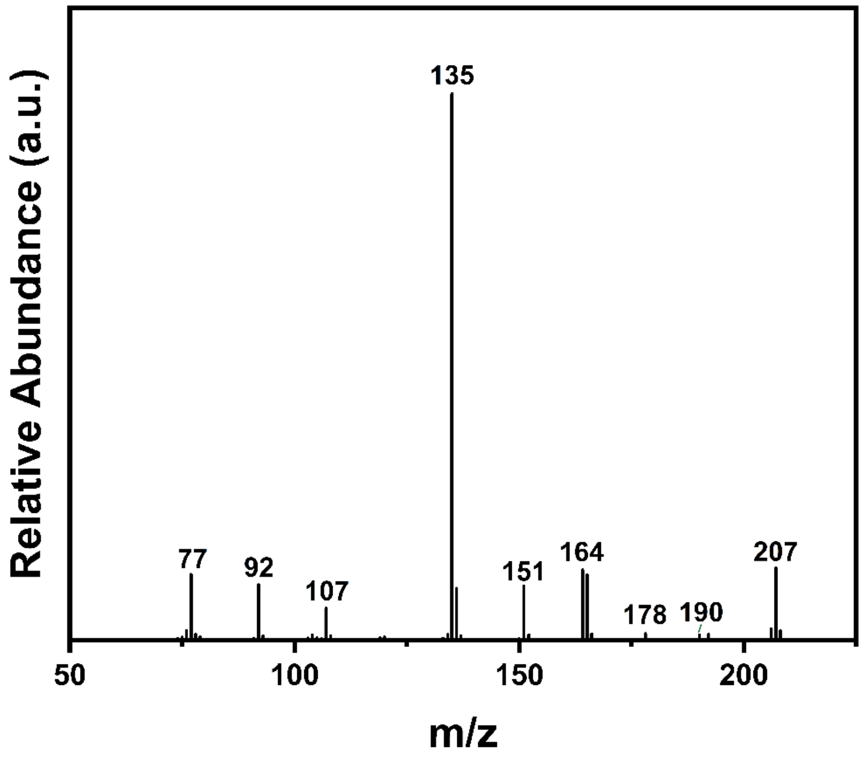

Standard Curve of compound (GC-MS):

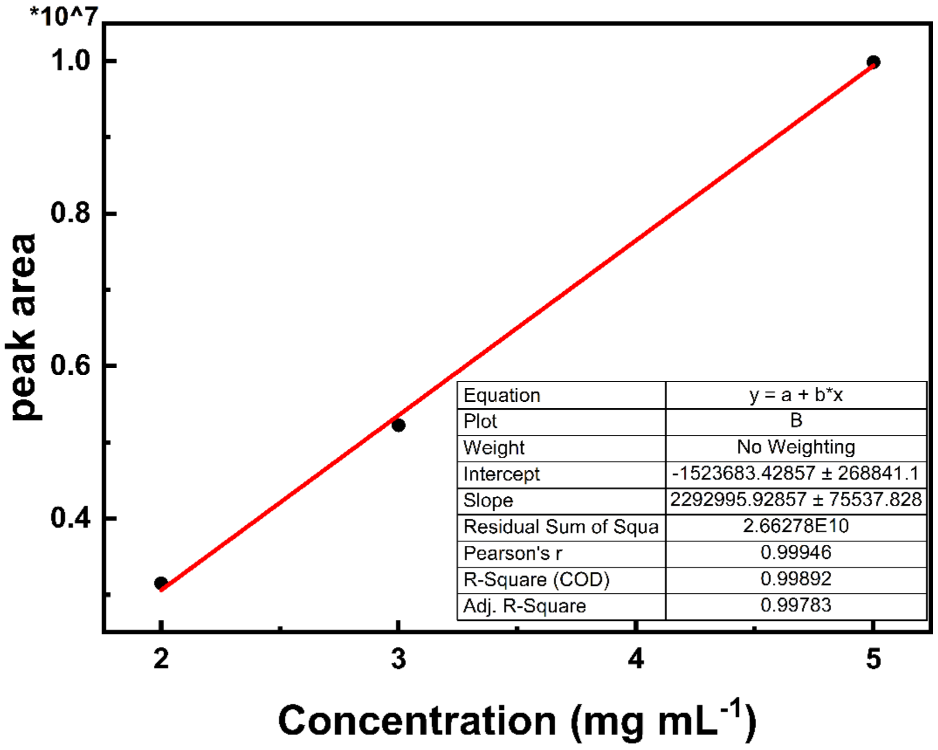

Supplementary Notes 14. N-butyl-4-cyanobenzamide[21]

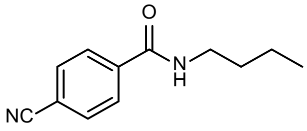

$C_{12}H_{14}N_2O$
M = 202.26 g mol^{-1}

^{1}H NMR: (400 MHz, Chloroform-*d*) δ 7.87–7.85 (m, 2H), 7.67–7.65 (m, 2H), 6.88 (t, *J* = 4.0 Hz, 1H), 3.41–3.36 (m, 2H), 1.58–1.51 (m, 2H), 1.39–1.29 (m, 2H), 0.89 (t, *J* = 8.0 Hz, 3H).

^{13}C NMR: (101 MHz, Chloroform-*d*) δ 165.7, 138.6, 132.2, 127.6, 118.0, 114.5, 39.9, 31.3, 20.0, 13.6.

Mass spectral data:

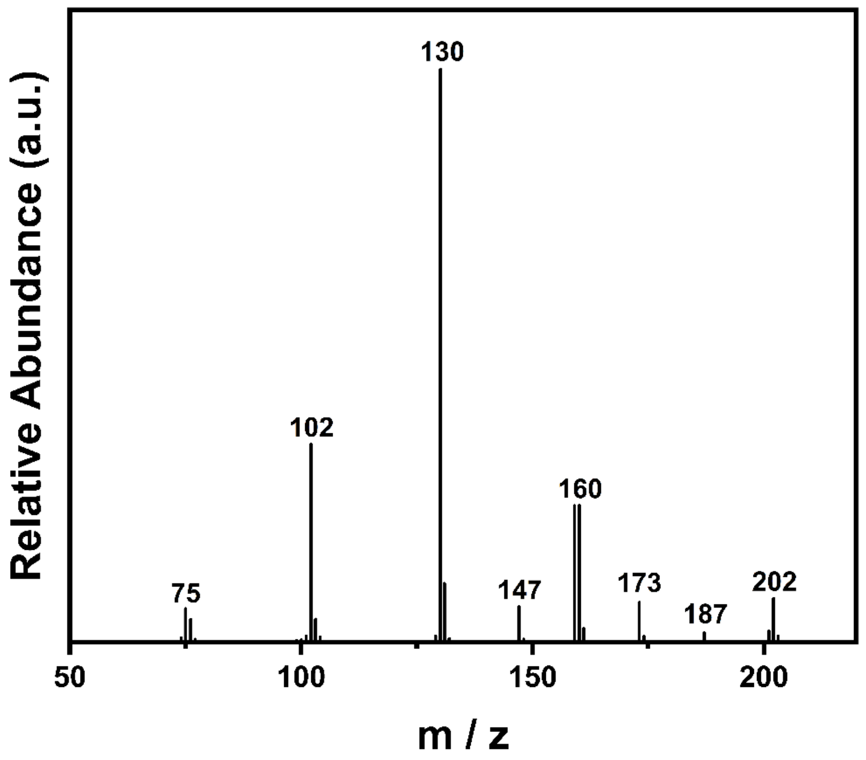

Standard Curve of compound (GC-MS):

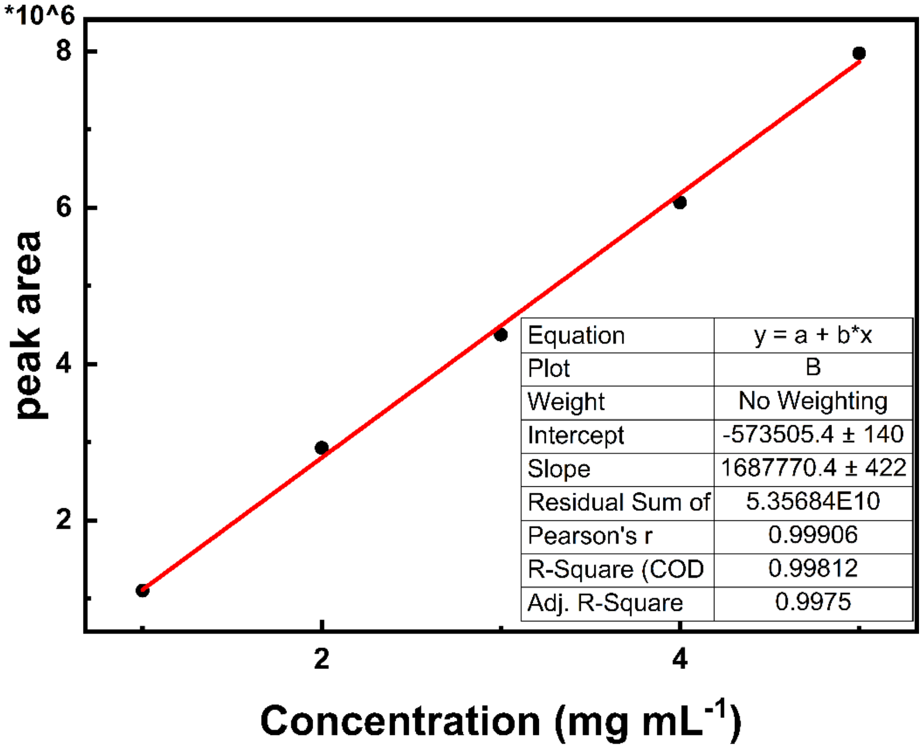

Supplementary Notes 15. N-((3s,5s,7s)-adamantan-1-yl)-4-methoxybenzamide[22]

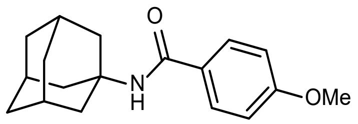

$C_{18}H_{23}NO_2$
M = 285.39 g mol^{-1}

^{1}H NMR: (400 MHz, Chloroform-*d*) δ 7.68–7.64 (m, 2H), 6.88 – 6.85 (m, 2H), 5.78 (s, 1H), 3.80 (s, 3H), 2.09 (s, 9H), 1.73 – 1.60 (m, 6H).

^{13}C NMR: (101 MHz, Chloroform-*d*) δ 166.1, 161.7, 128.4, 113.4, 55.3, 52.0, 41.6, 36.4, 36.3, 29.4.

Mass spectral data:

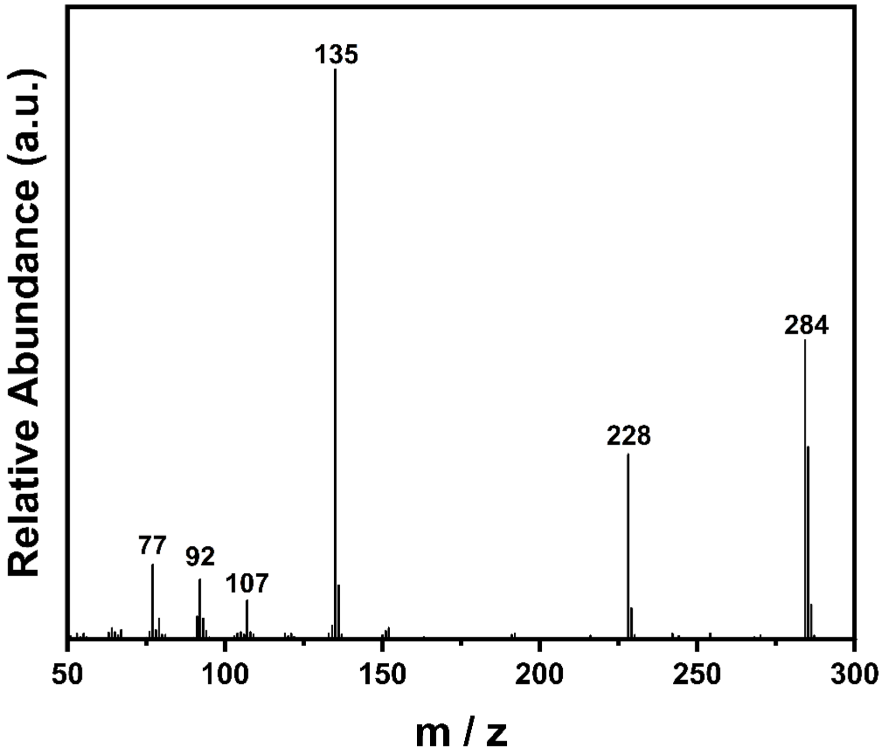

Standard Curve of compound (GC-MS):

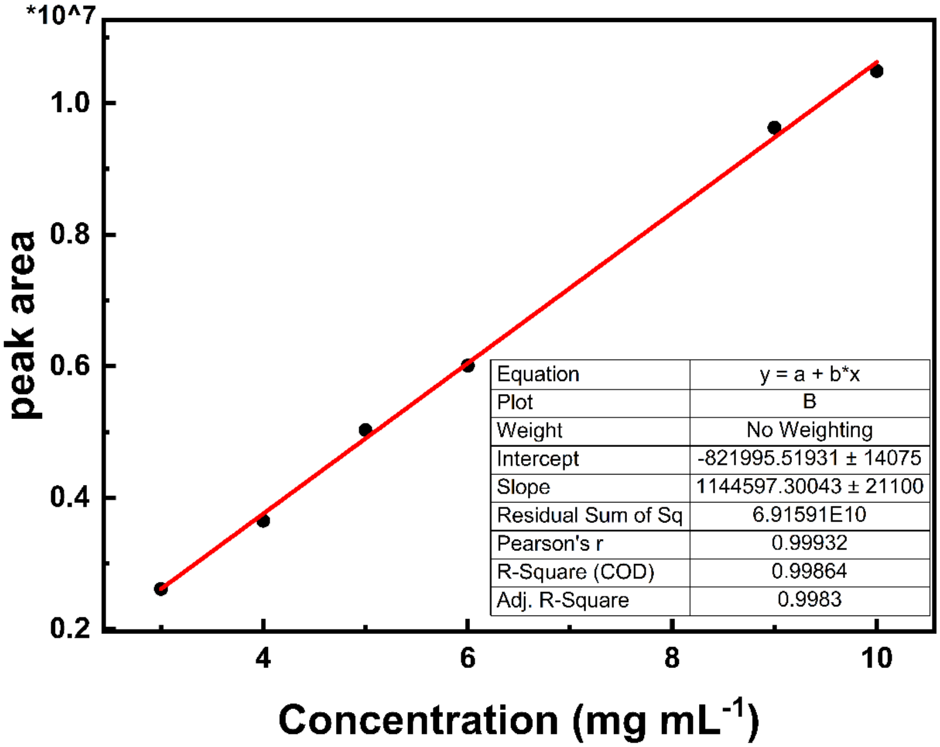

Supplementary Notes 16. L-methyl (4-methoxybenzoyl) alaninate

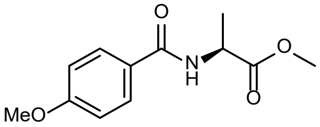

$C_{12}H_{15}NO_4$
M = 237.26 g mol^{-1}

^{1}H NMR: (400 MHz, Chloroform-*d*) δ 7.79–7.75 (m, 2H), 6.95–6.91 (m, 2H), 6.64 (d, *J* = 8 Hz, 1H), 4.83–4.76 (m, 1H), 3.82 (d, *J* = 24.0 Hz, 6H), 1.51 (d, *J* = 8 Hz, 3H).

^{13}C NMR: (101 MHz, Chloroform-*d*) δ 173.9, 166.3, 162.3, 128.9, 126.1, 113.7, 55.4, 52.6, 48.4, 18.8.

HRMS (m / z)**:** [M + H$^+$] calcd for $[C_{12}H_{15}NO_4H]^+$, 238.1079; found, 238.1079.

Mass spectral data:

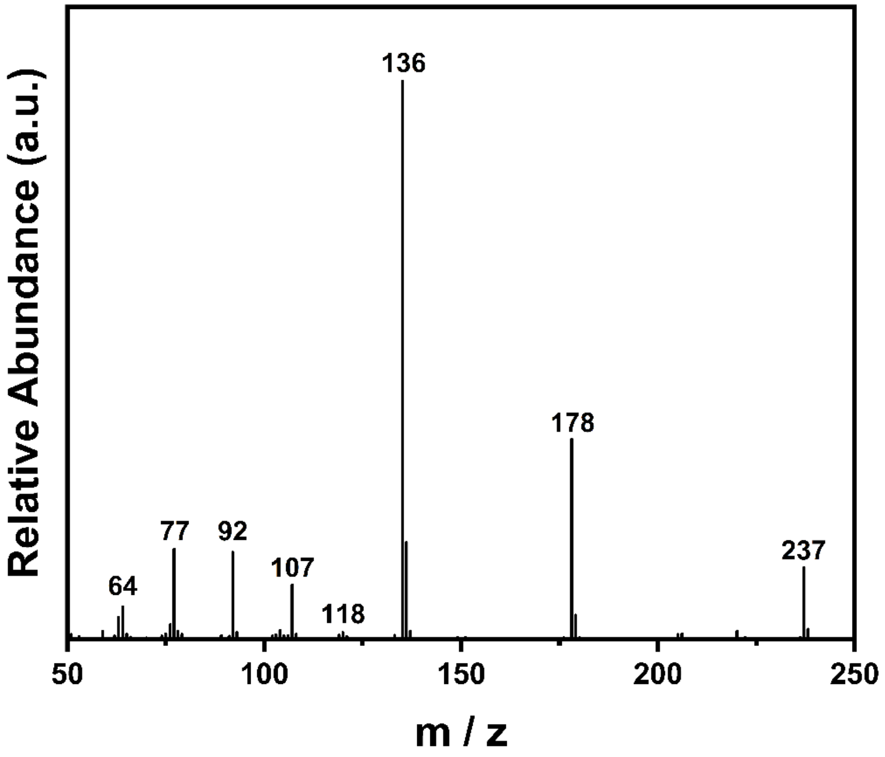

Standard Curve of compound (GC-MS):

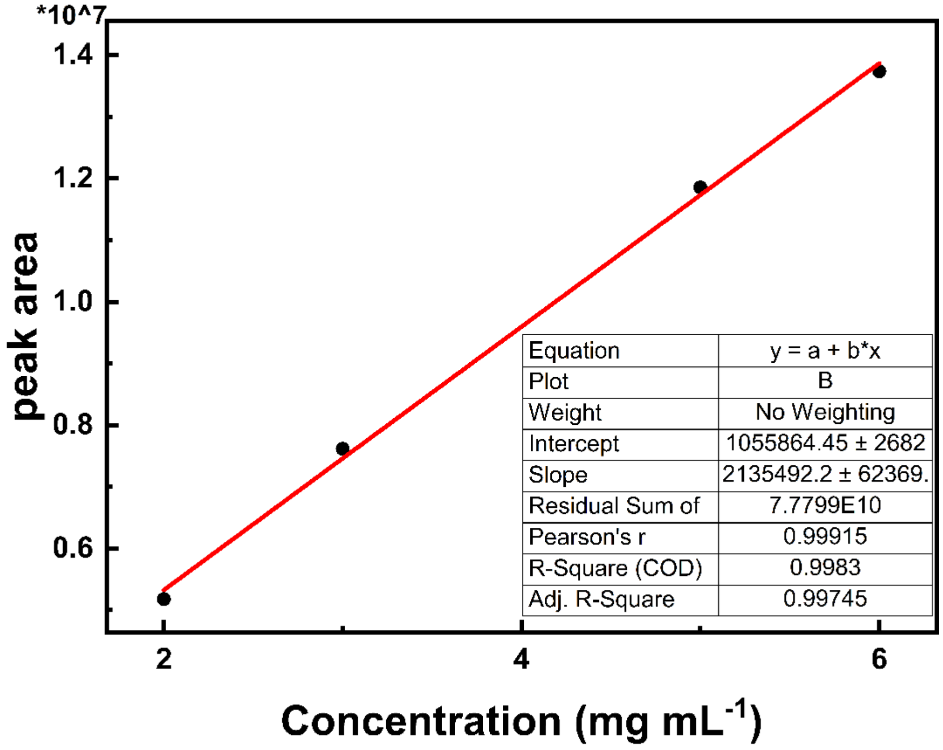

Supplementary Notes 17. D-methyl (4-methoxybenzoyl) prolinate

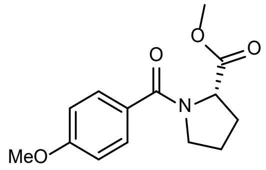

$C_{14}H_{17}NO_4$
M = 263.29 g mol^{-1}

^{1}H NMR: (400 MHz, Chloroform-*d*) δ 7.50 (d, *J* = 8.6 Hz, 2H), 6.82 (d, *J* = 8.6 Hz, 2H), 4.81–4.54 (m, 1H), 3.74 (s, 3H), 3.68 (s, 3H), 3.64–3.59 (m, 1H), 3.55–3.50 (m, 2H), 2.26–2.19 (m, 1H), 1.97–1.88 (m, 3H), 1.85–1.79 (m, 1H).

^{13}C NMR: (101 MHz, Chloroform-*d*) δ 172.7, 169.0, 160.9, 129.2, 113.1, 59.1, 55.1, 51.9, 49.9, 29.1, 25.3.

HRMS (m / z)**:** [M + H$^+$] calcd for $[C_{14}H_{17}NO_4H]^+$, 264.1236; found, 264.1236.

Mass spectral data:

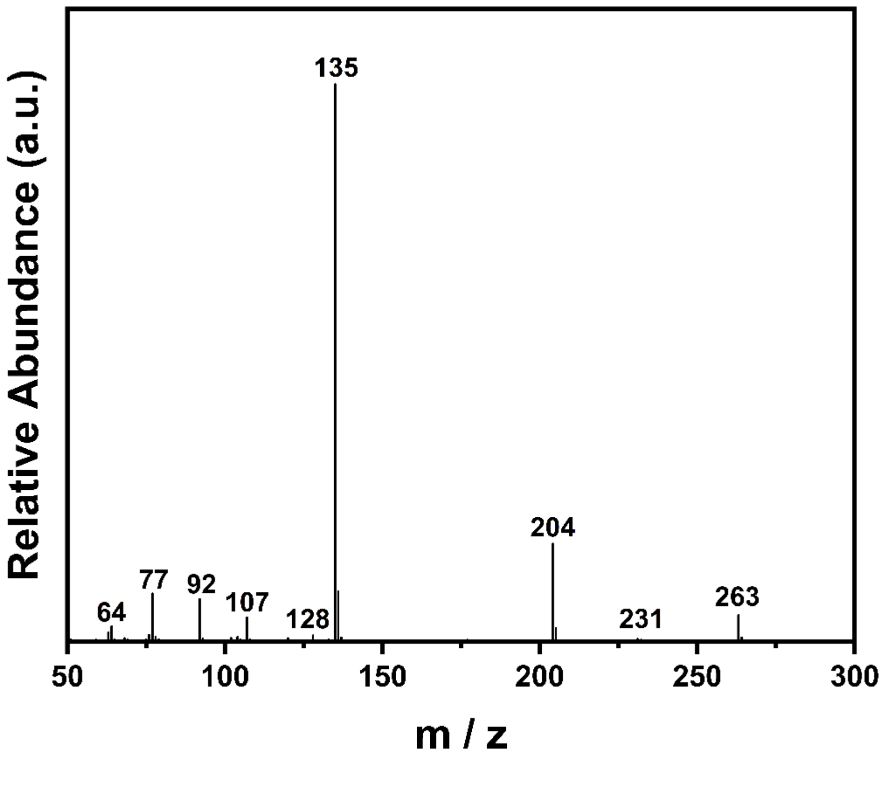

Standard Curve of compound (GC-MS):

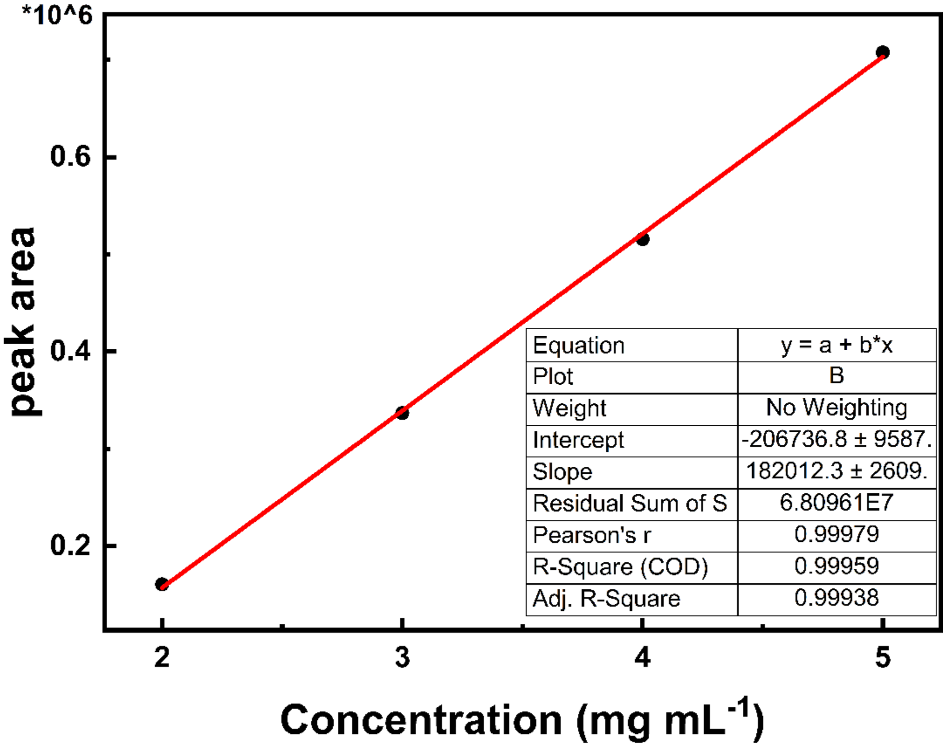

Supplementary Notes 18. 2-chloro-N-(4'-chloro-[1,1'-biphenyl]-2-yl) nicotinamide **(Boscalid)**[20]

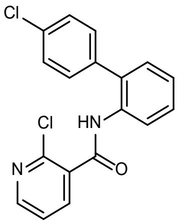

$C_{18}H_{12}Cl_2N_2O$
M = 343.21 g mol^{-1}

^{1}H NMR: (400 MHz, Chloroform-d) δ 8.46–8.41 (m, 2H), 8.16-8.13 (m, 2H), 7.48–7.42 (m, 3H), 7.37 – 7.32 (m, 3H), 7.27 (d, J = 0.8 Hz, 1H).

^{13}C NMR: (101 MHz, Chloroform-d) δ 162.5, 151.4, 146.7, 140.2, 136.2, 134.4, 134.3, 132.2, 131.0, 130.8, 130.2, 129.3, 128.9, 125.3, 122.9, 122.0.

Because of the high boiling point of the Boscalid, mass spectrometry cannot identify the components. Concentrations of analytical samples were calculated by an internal standard

method using anicole as the internal standard. The yield was determined by 1H NMR by internal standard method (55.0umol).

Supplementary Notes 19. (4-methoxyphenyl) (phenyl) methanone[24]

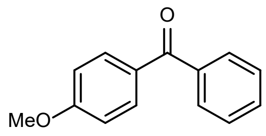

$C_{14}H_{12}O_2$

M = 212.25 g mol^{-1}

^{1}H NMR (400 MHz, Chloroform-*d*): δ 7.85–7.81 (m, 2H), 7.77–7.73 (m, 2H), 7.59–7.53 (m, 1H), 7.49–7.45 (m, 2H), 6.96–6.94 (m, 2H), 3.89 (s, 3H).

^{13}C NMR (101 MHz, Chloroform-*d*): δ 195.5, 163.2, 138.2, 132.5, 131.8, 130.1, 129.7, 128.1, 113.5, 55.5.

Mass spectral data:

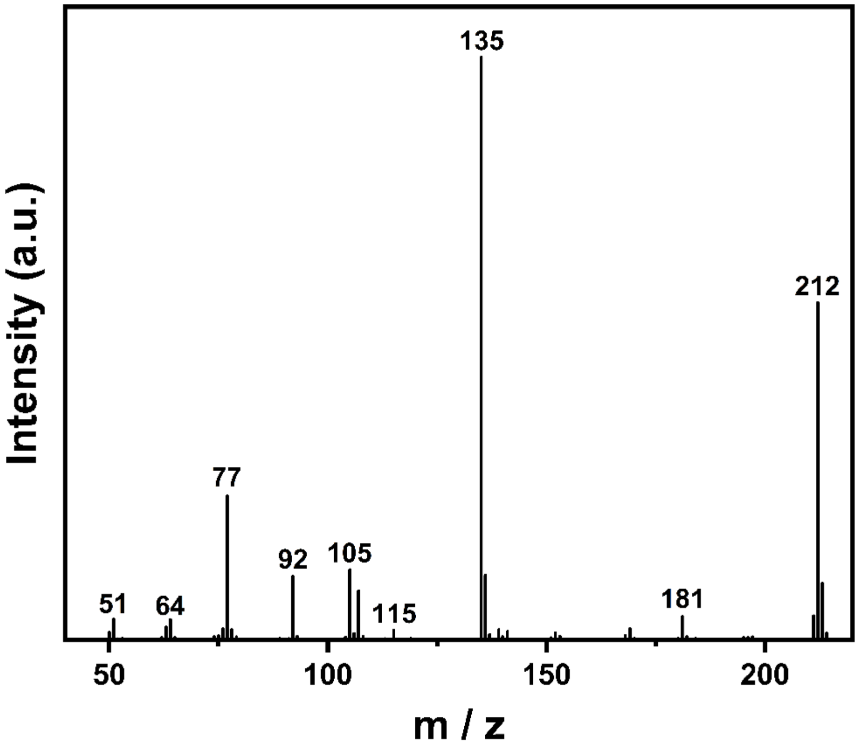

Standard Curve of compound (GC-MS):

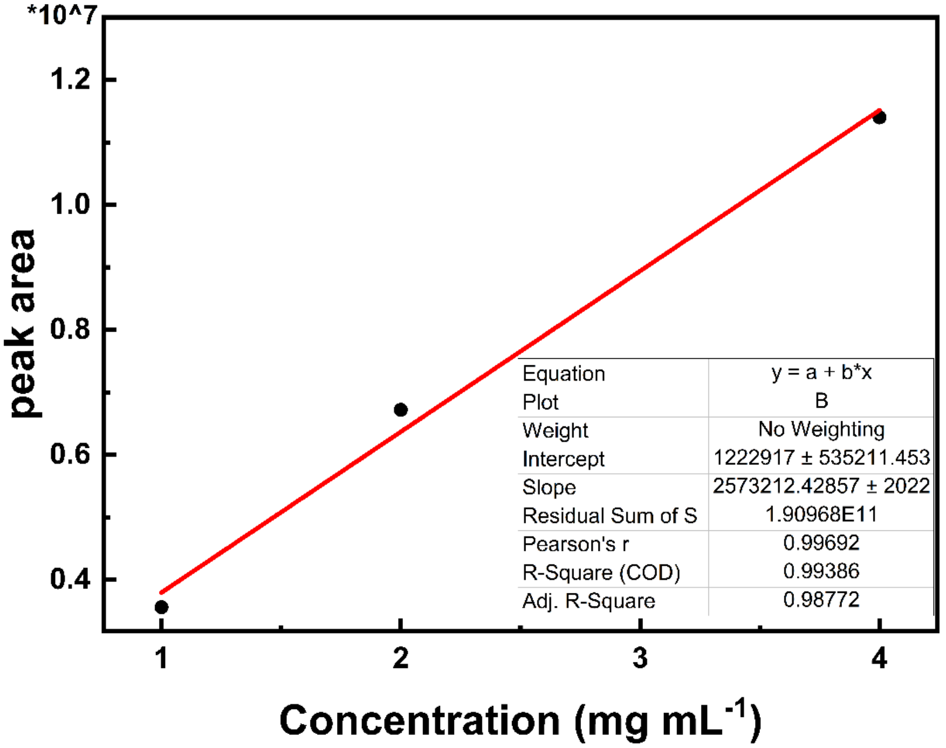

Supplementary Notes 20. Ethyl 4-methoxybenzoate[25]

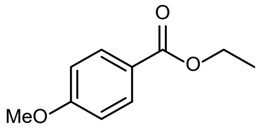

$C_{10}H_{12}O_3$

M = 180.20 g mol^{-1}

^{1}H NMR (400 MHz, Chloroform-*d*): δ 8.00–7.97 (m, 2H), 6.92–6.88 (m, 2H), 4.33 (q, *J* = 7.1 Hz, 2H), 3.84 (s, 3H), 1.37 (t, *J* = 7.1 Hz, 3H).

^{13}C NMR (101 MHz, Chloroform-*d*): δ 166.3, 163.2, 131.4, 122.8, 113.4, 60.6, 55.3, 14.3.

Mass spectral data:

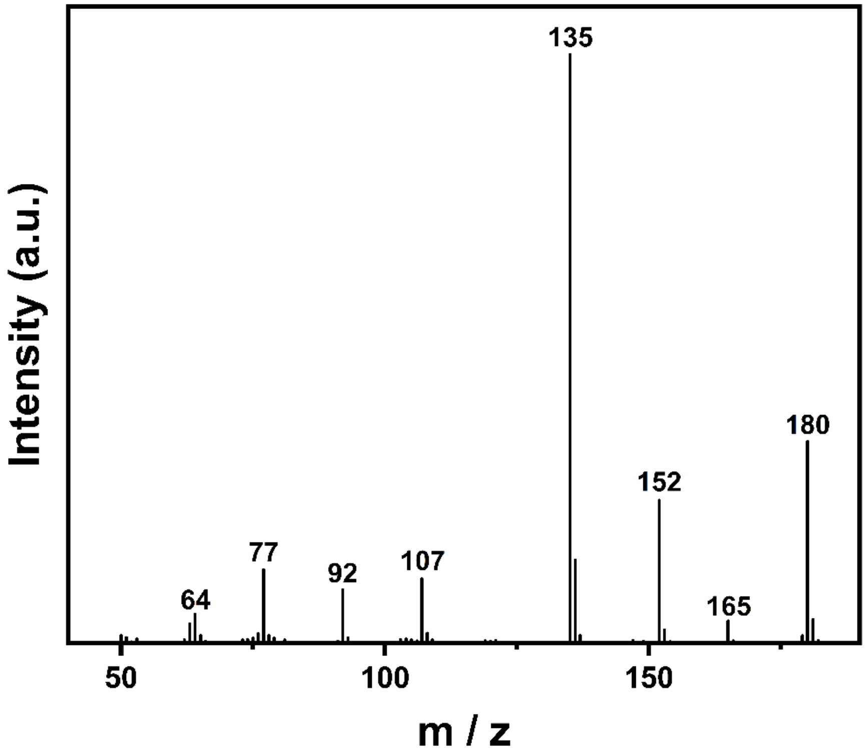

Standard Curve of compound (GC-MS):

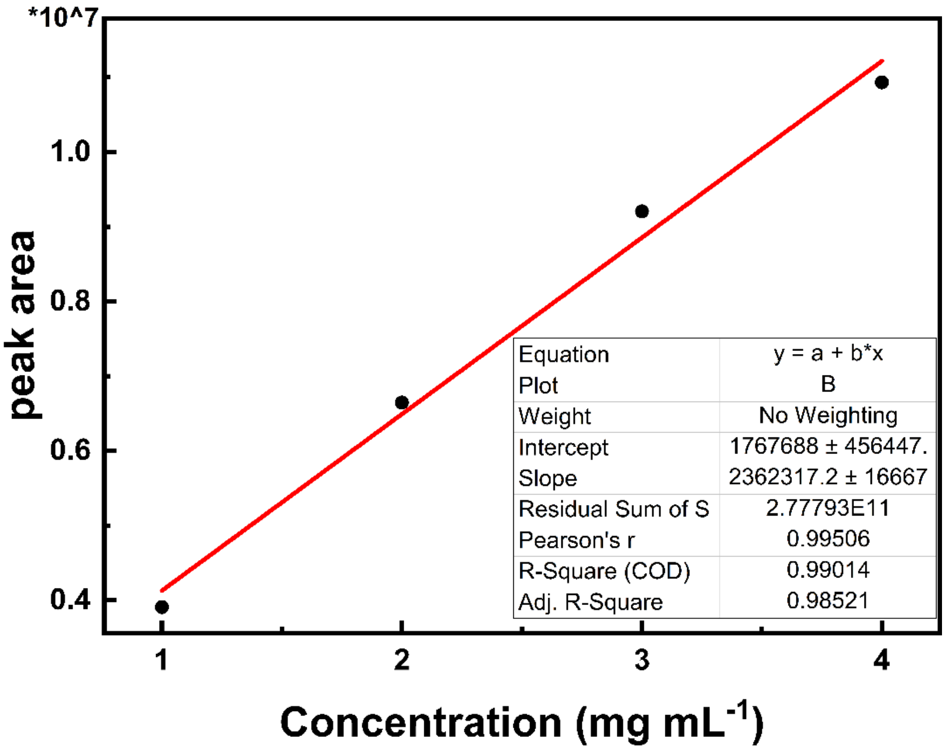

Supplementary Notes 21. phenyl 4-methoxybenzoate[26]

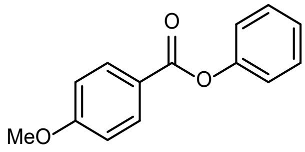

$C_{14}H_{12}O_3$

M = 228.25 g mol^{-1}

^{1}H NMR (400 MHz, Chloroform-*d*): δ 8.18–8.15 (m, 2H), 7.45–7.41 (m, 2H), 7.29–7.20 (m, 3H), 7.01–6.97 (m, 2H), 3.90 (s, 3H).

^{13}C NMR (101 MHz, Chloroform-*d*): δ 164.9, 163.8, 151.0, 132.3, 129.4, 125.7, 121.8, 121.8, 113.8, 55.5.

Mass spectral data:

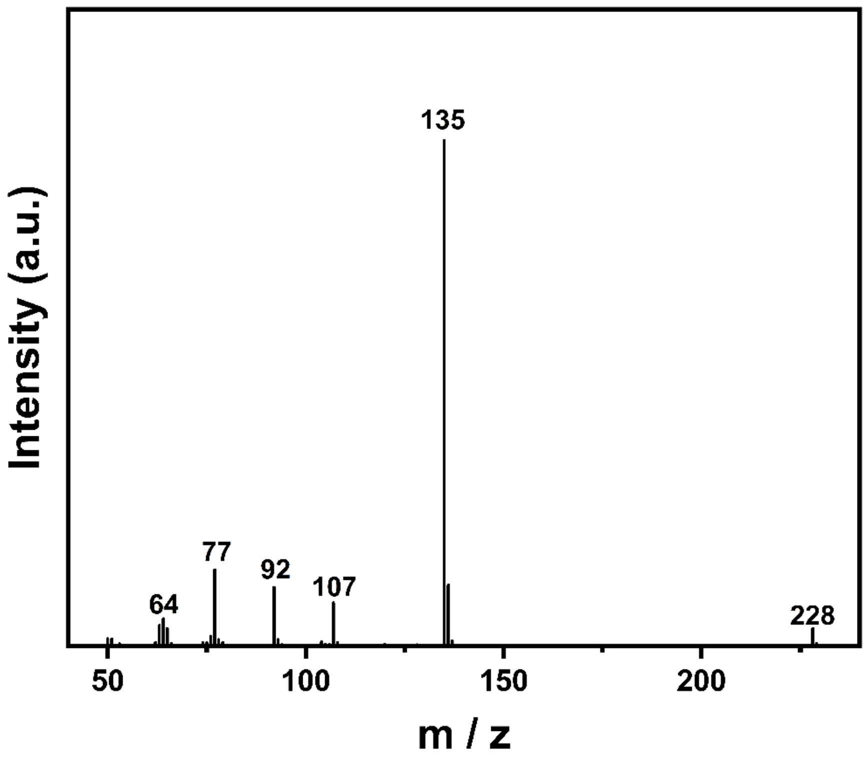

Standard Curve of compound (GC-MS):

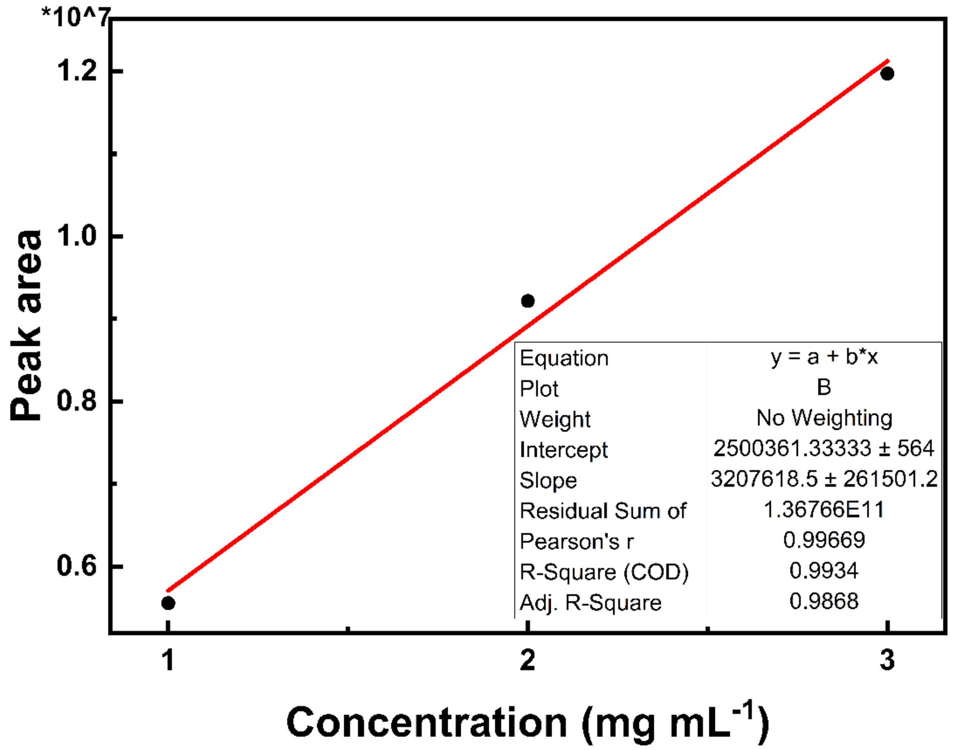

^{1}H- and ^{13}C-NMR Spectra

1. N,N-diethyl-3-methylbenzamide **(DEET)**

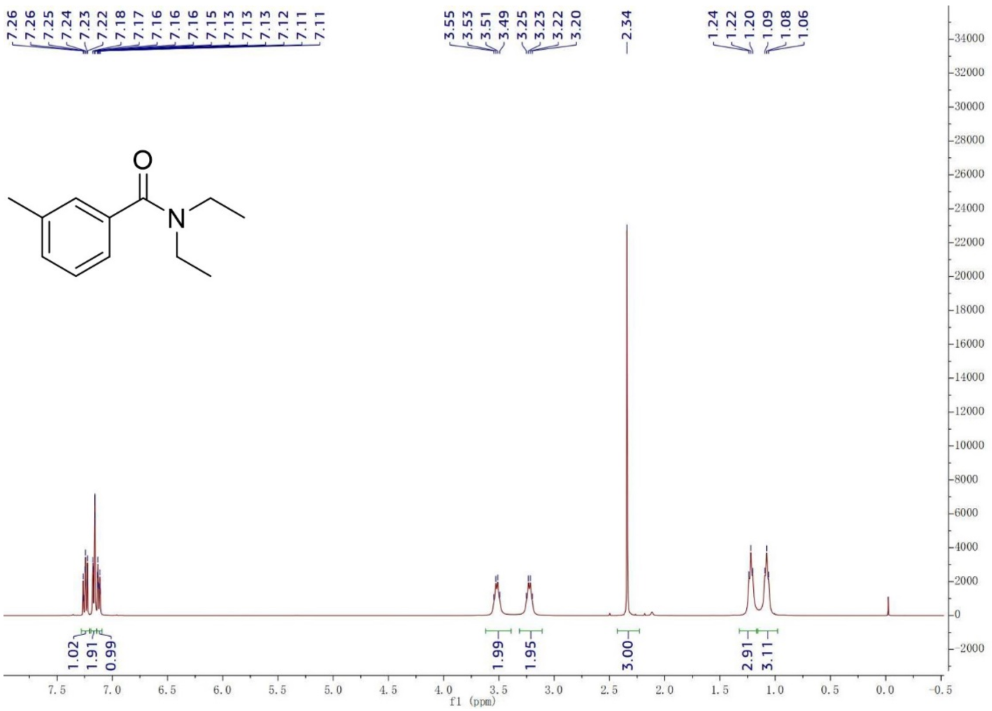

^{1}H NMR spectrum of product **1** in $CDCl_3$

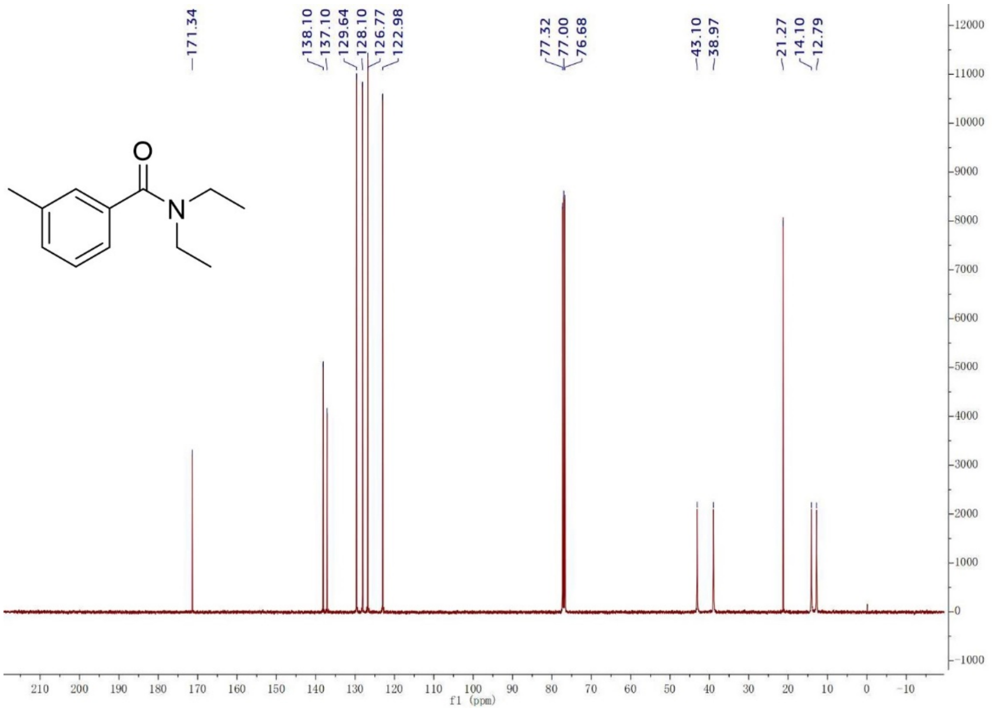

^{13}C NMR spectrum of product **1** in $CDCl_3$

2. 2-chloro-N-(4'-chloro-[1,1'-biphenyl]-2-yl) nicotinamide **(Boscalid)**

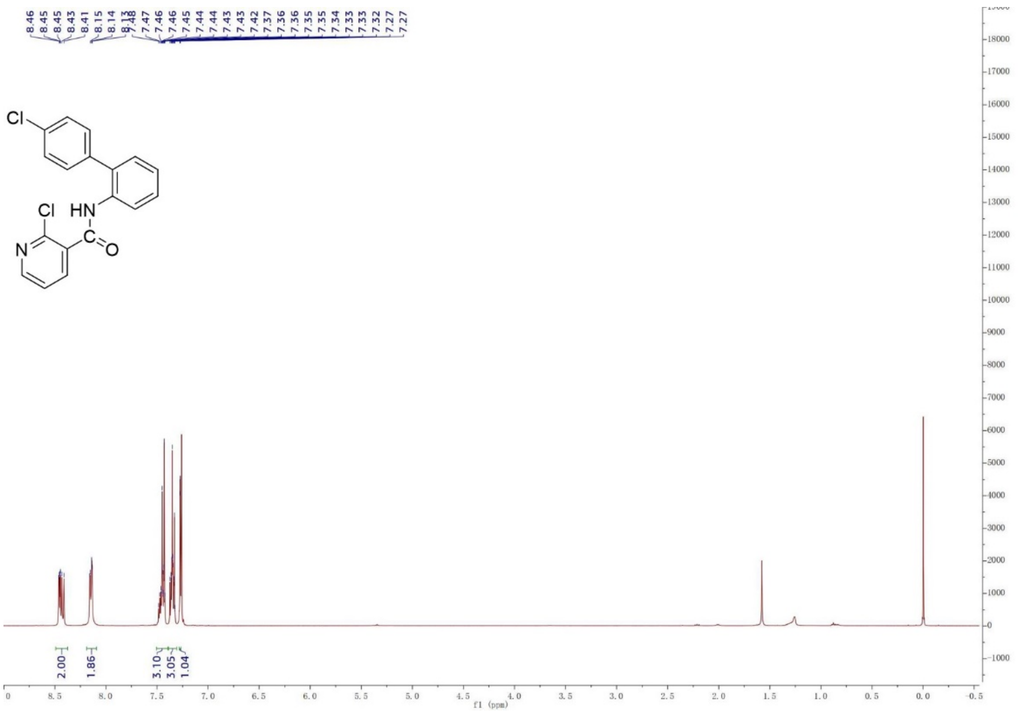

^{1}**H NMR** spectrum of product **2** in $CDCl_3$

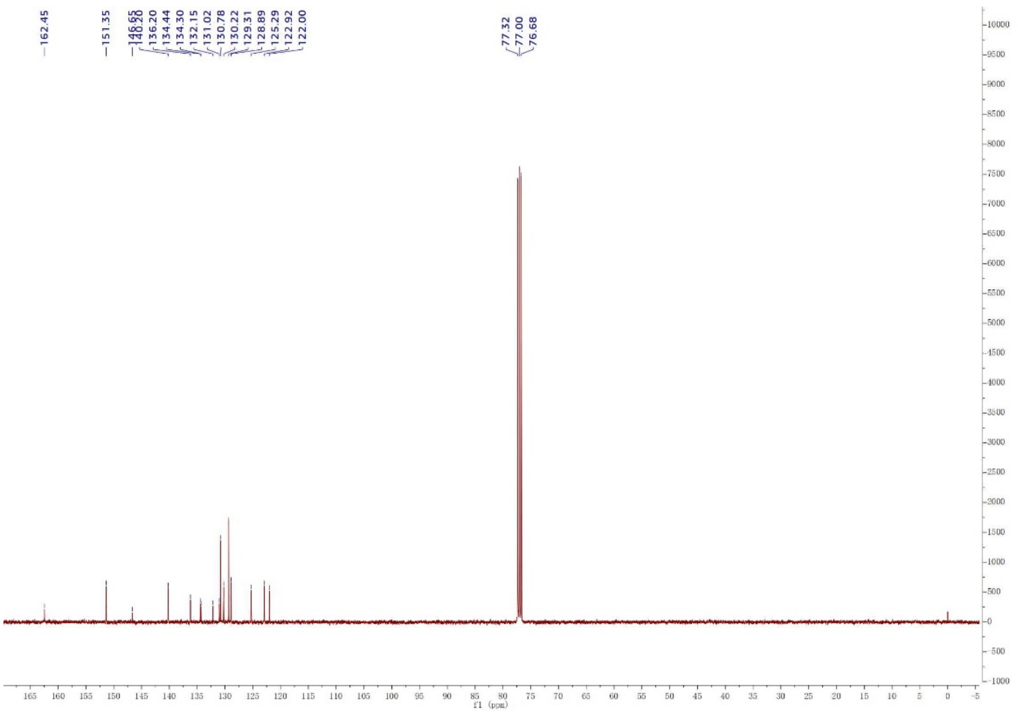

^{13}**C NMR** spectrum of product **2** in $CDCl_3$

3. N-butyl-4-methoxybenzamide

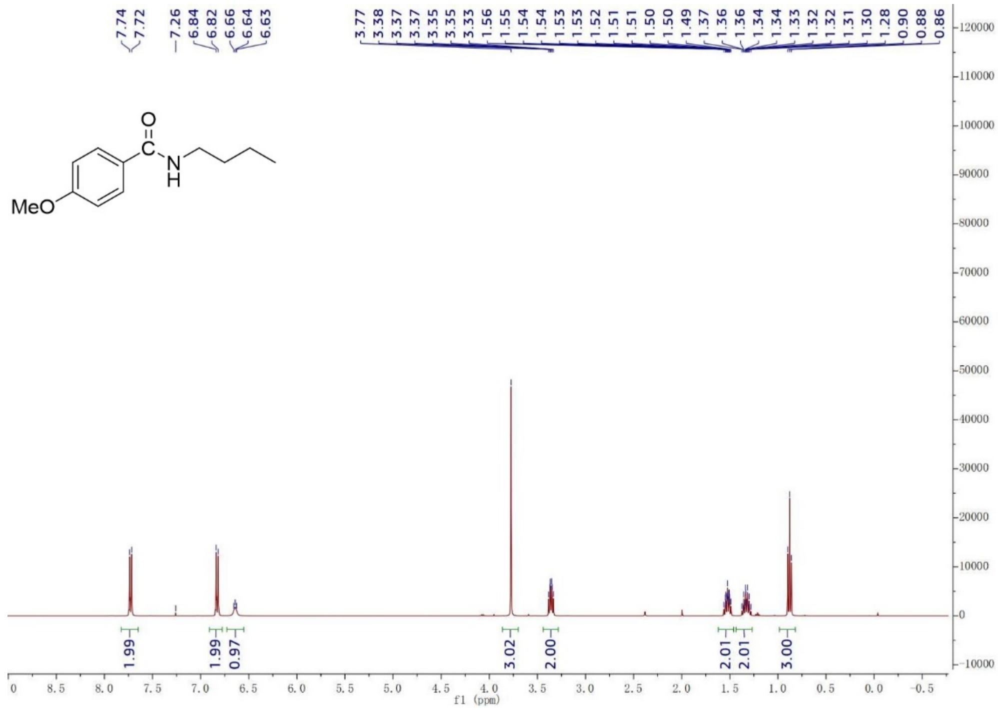

^{1}**H NMR** spectrum of product **3** in $CDCl_3$

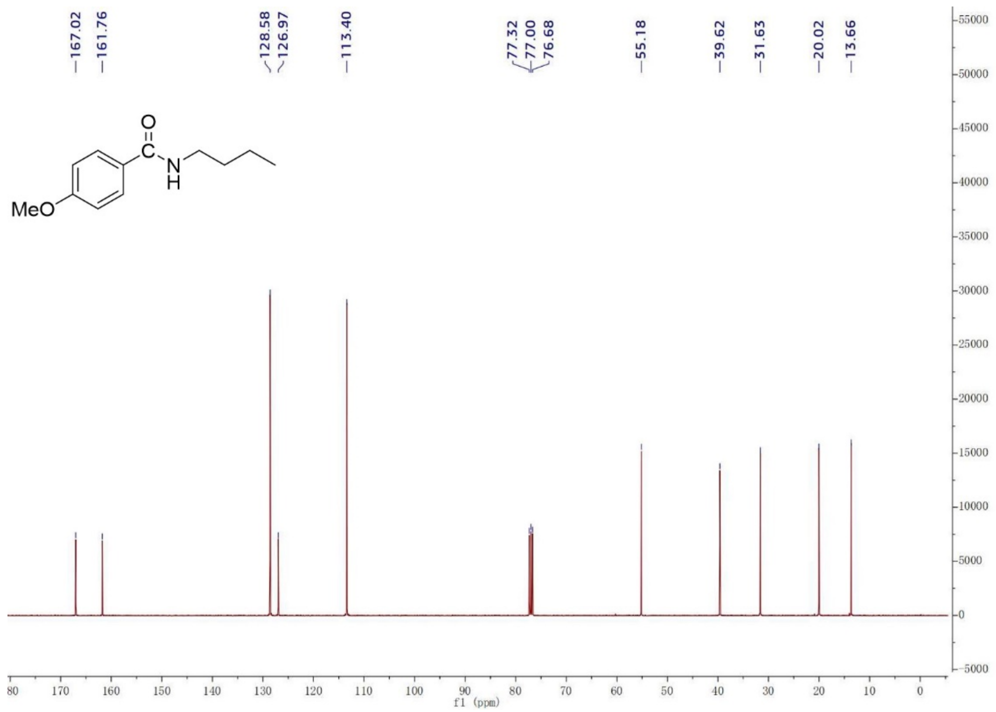

^{13}**C NMR** spectrum of product **3** in $CDCl_3$

4. N-((3s,5s,7s)-adamantan-1-yl)-4-methoxybenzamide

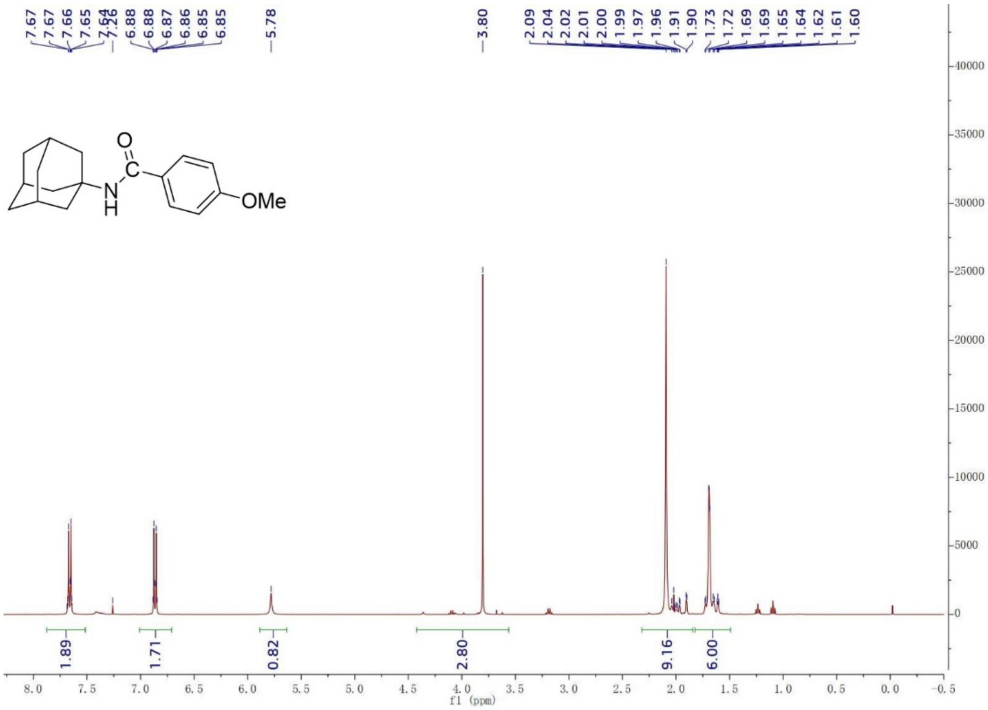

^{1}H NMR spectrum of product **4** in $CDCl_3$

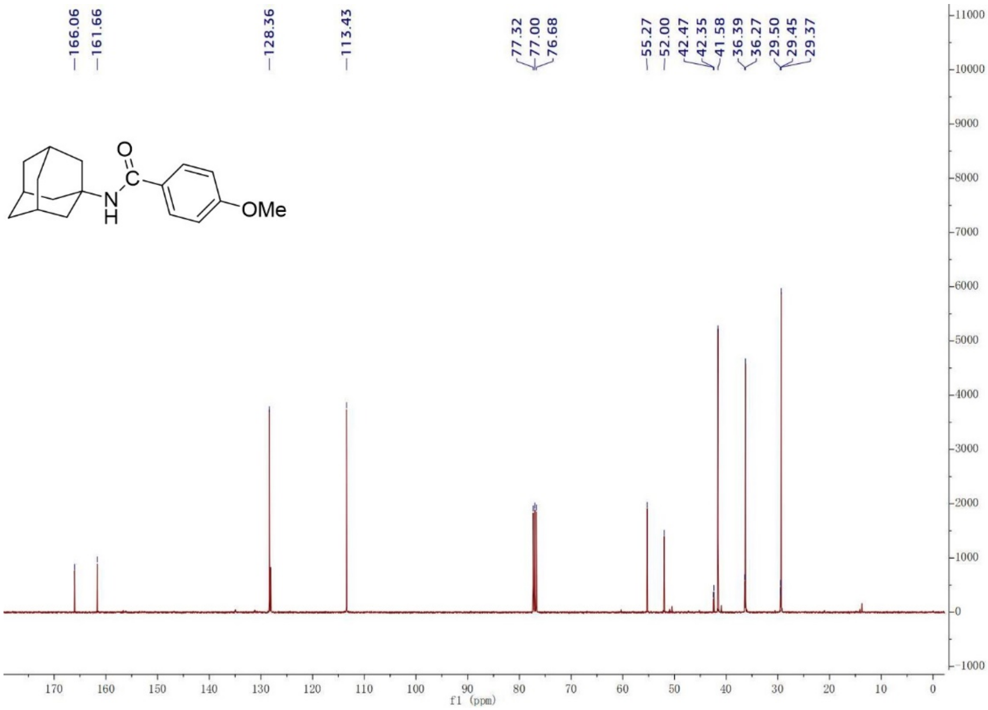

^{13}C NMR spectrum of product **4** in $CDCl_3$

5. L-methyl (4-methoxybenzoyl) alaninate

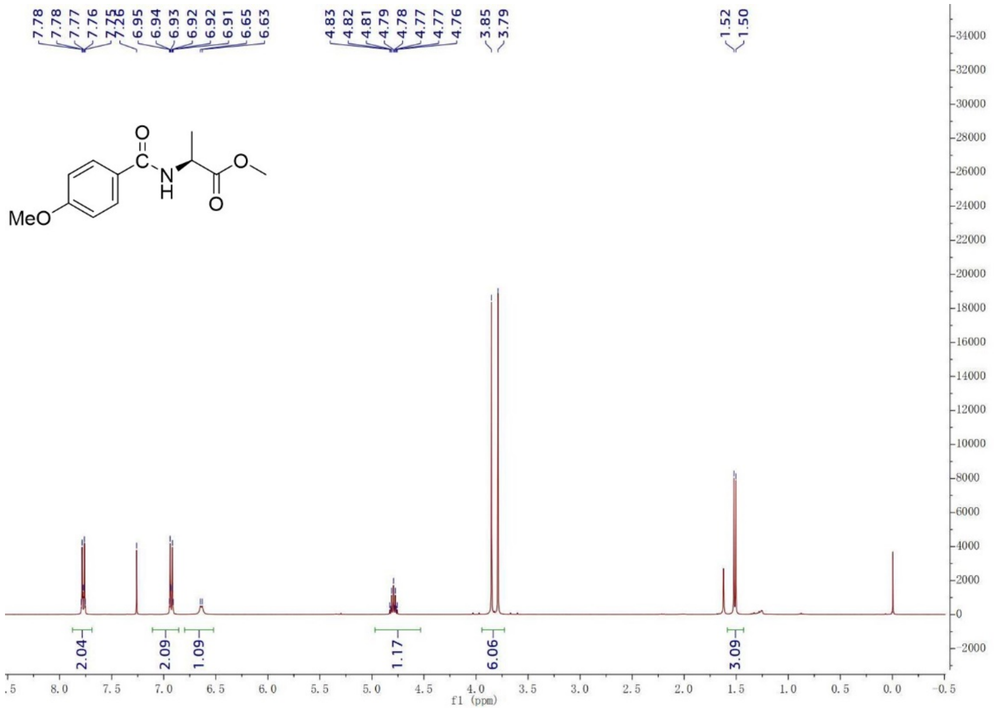

^{1}**H NMR** spectrum of product **5** in $CDCl_3$

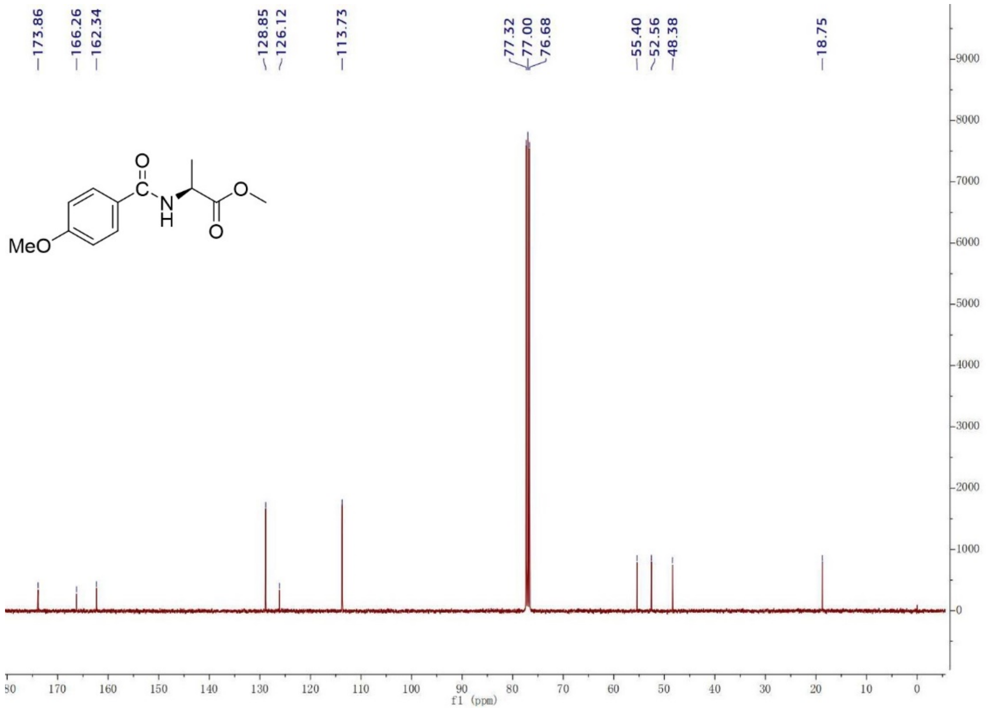

^{13}**C NMR** spectrum of product **5** in $CDCl_3$

6. D-methyl (4-methoxybenzoyl) prolinate (Keto-Enol Tautomerism = 4:1)

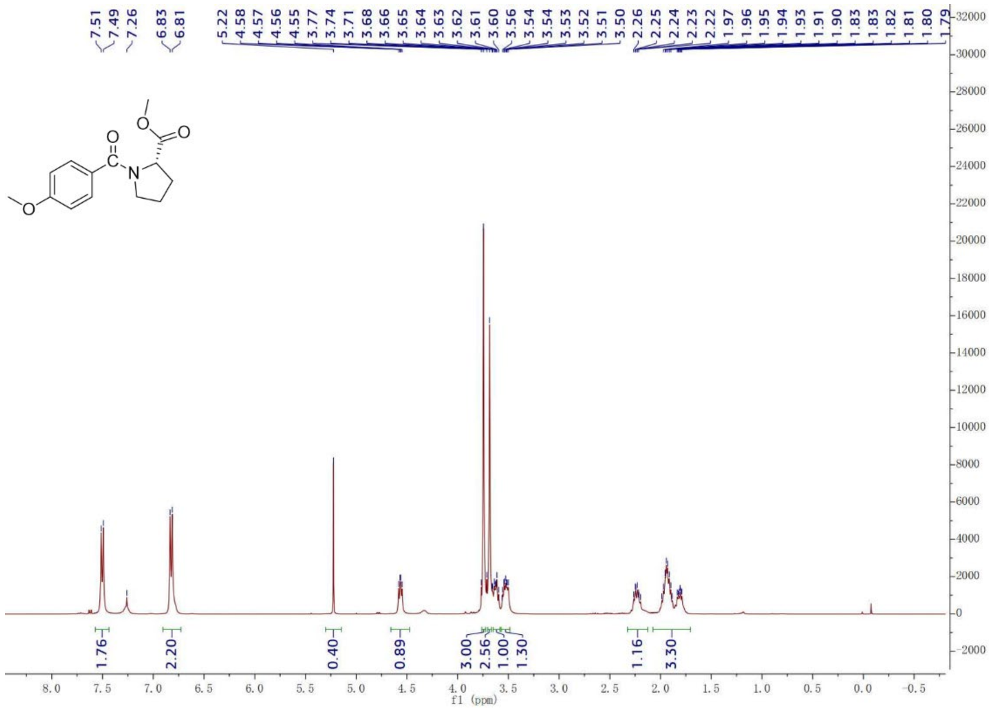

^{1}H NMR spectrum of product **6** in $CDCl_3$

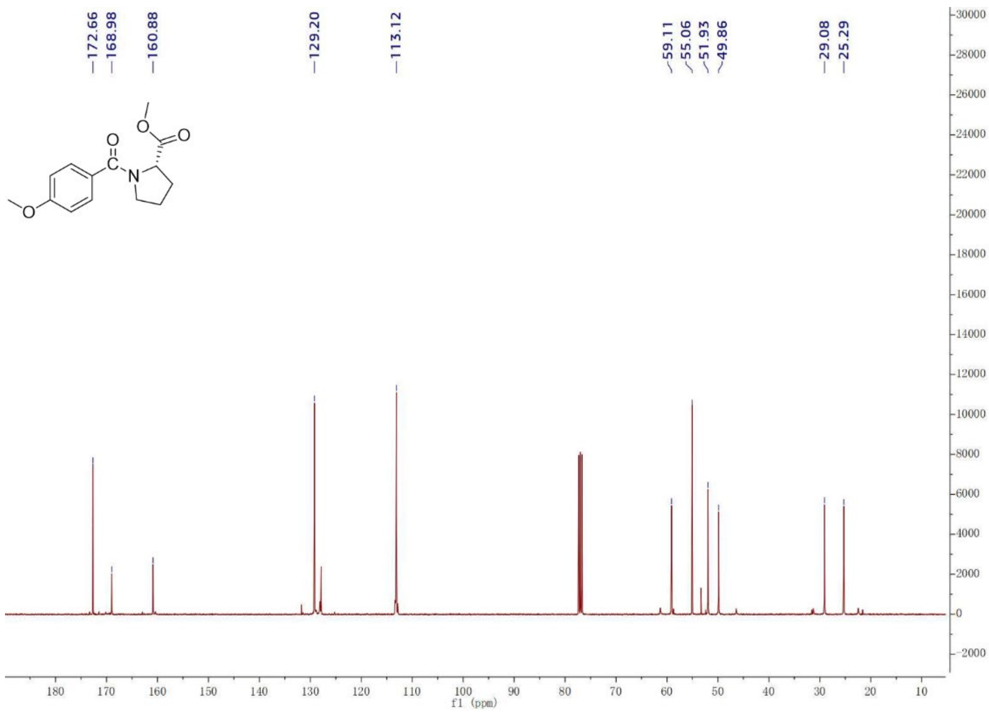

^{13}C NMR spectrum of product **6** in $CDCl_3$

7. N-phenylbenzamide

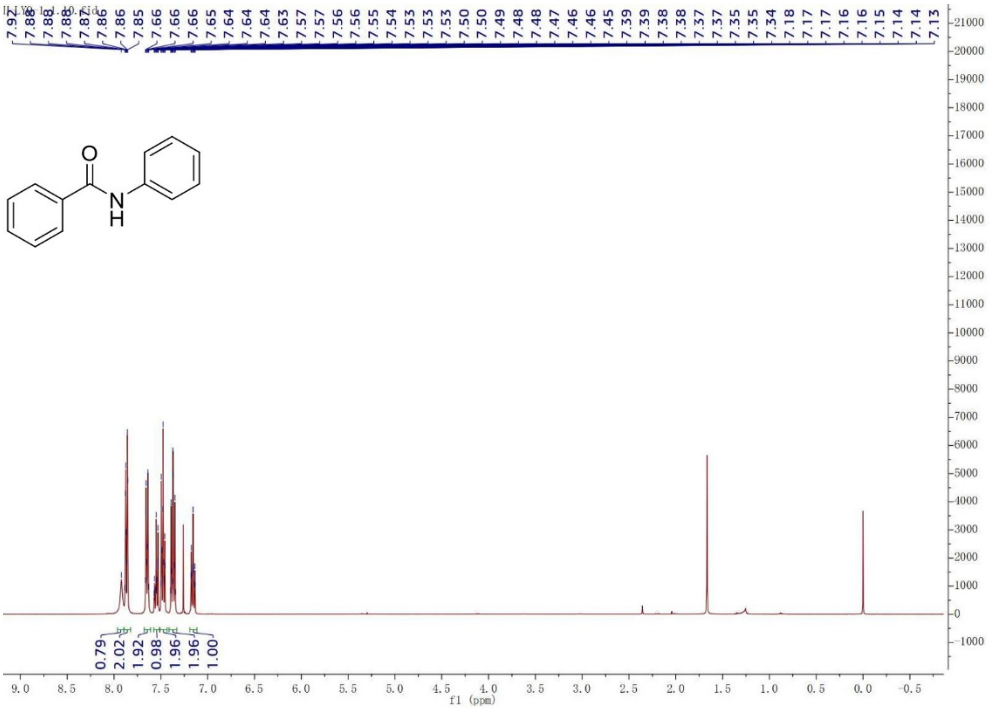

^{1}H NMR spectrum of product **7** in $CDCl_3$

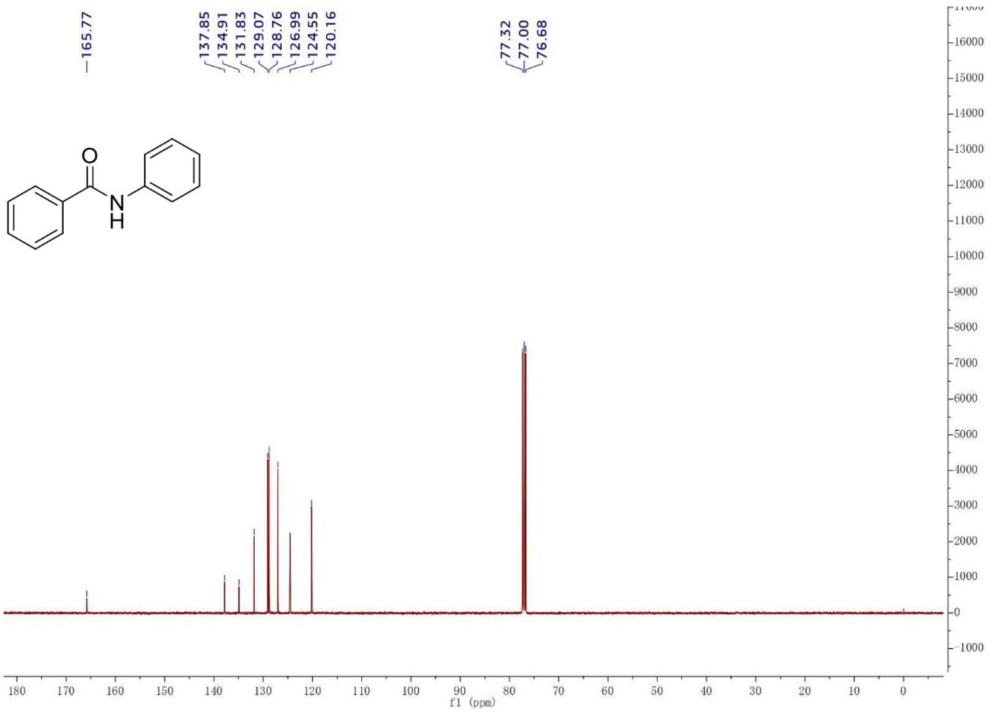

^{13}C NMR spectrum of product **7** in $CDCl_3$

8. N-benzylbenzamide

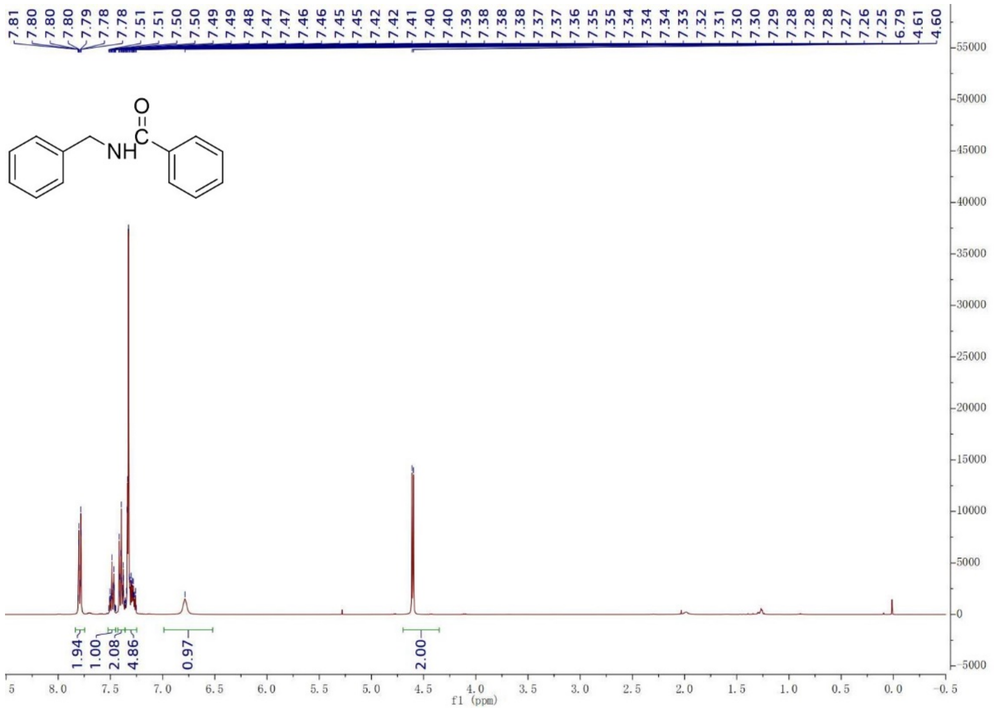

^{1}**H NMR** spectrum of product **8** in $CDCl_3$

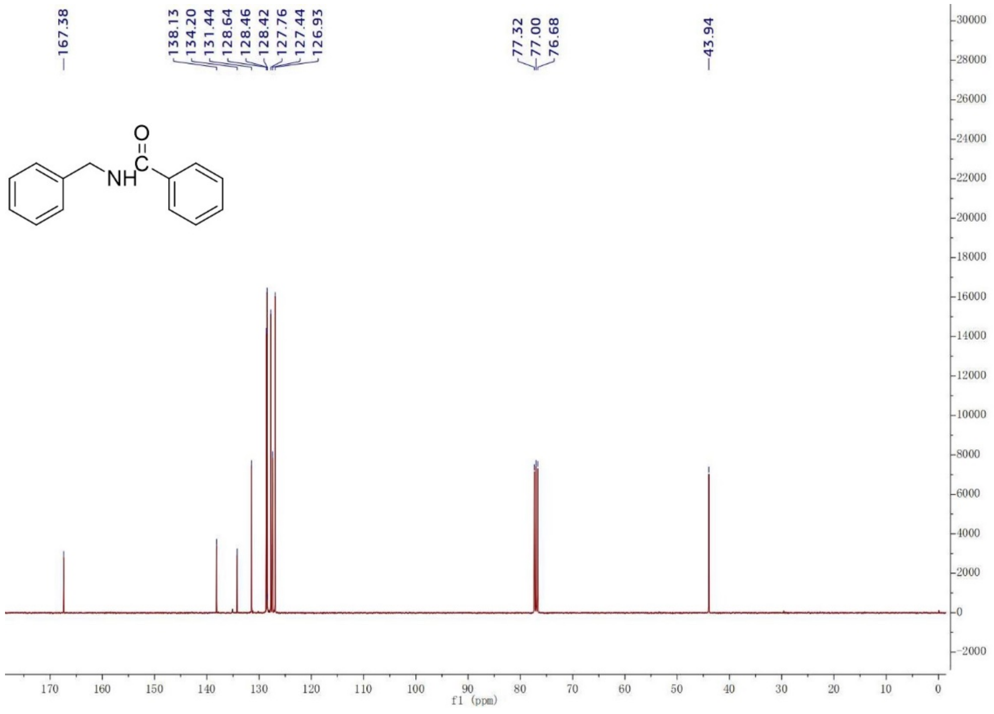

^{13}**C NMR** spectrum of product **8** in $CDCl_3$

9. N-butylbenzamide

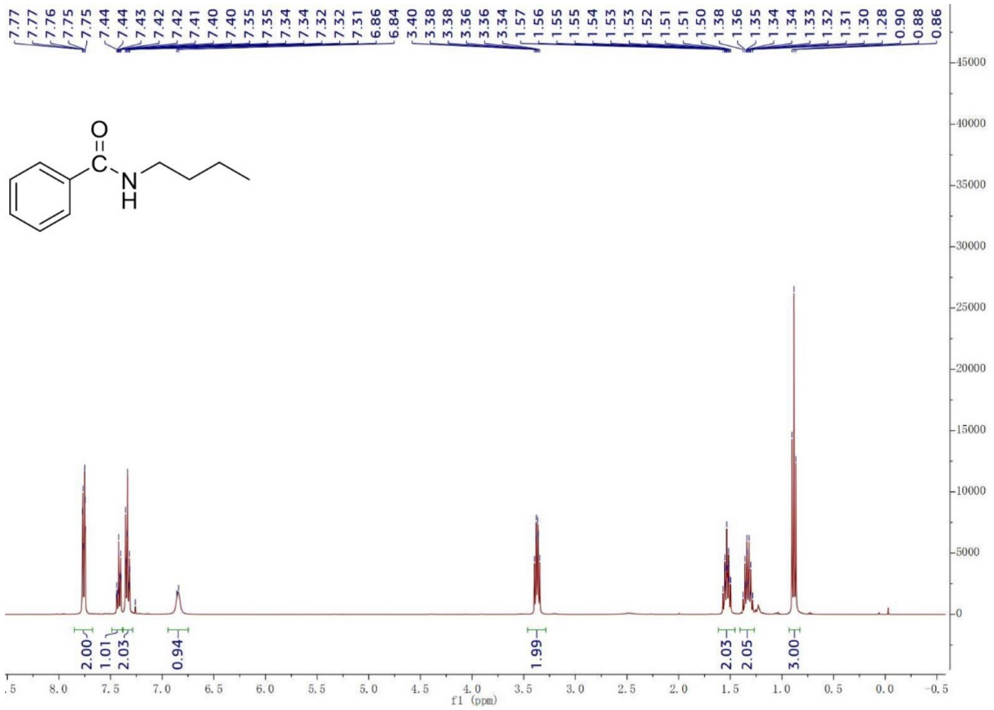

^{1}H NMR spectrum of product **9** in $CDCl_3$

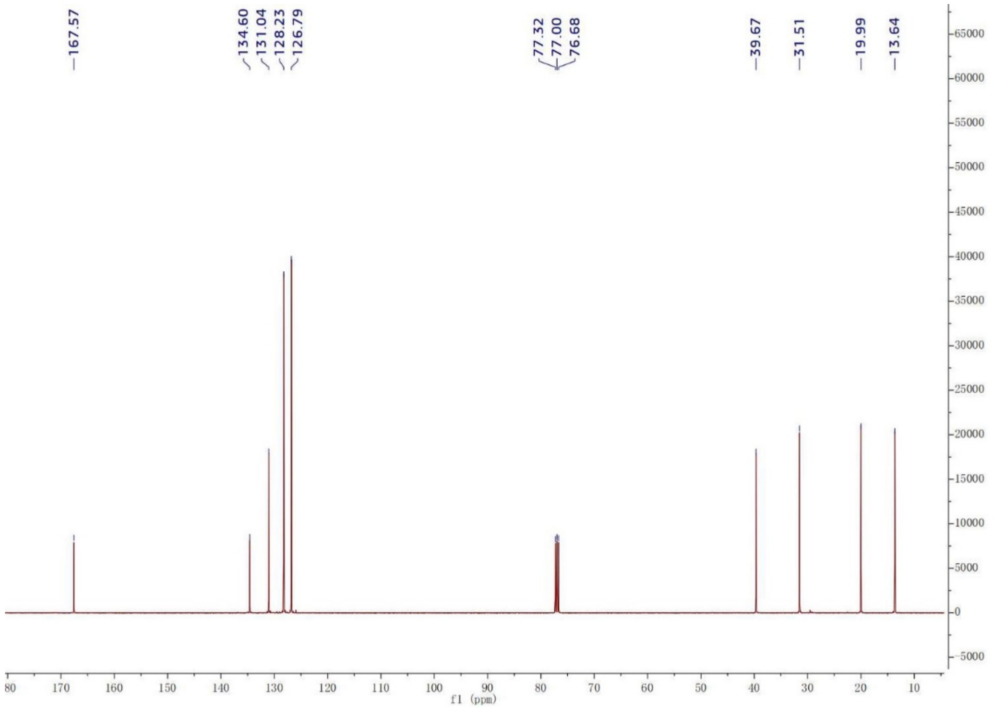

^{13}C NMR spectrum of product **9** in $CDCl_3$

10. N-butyl-4-cyanobenzamide

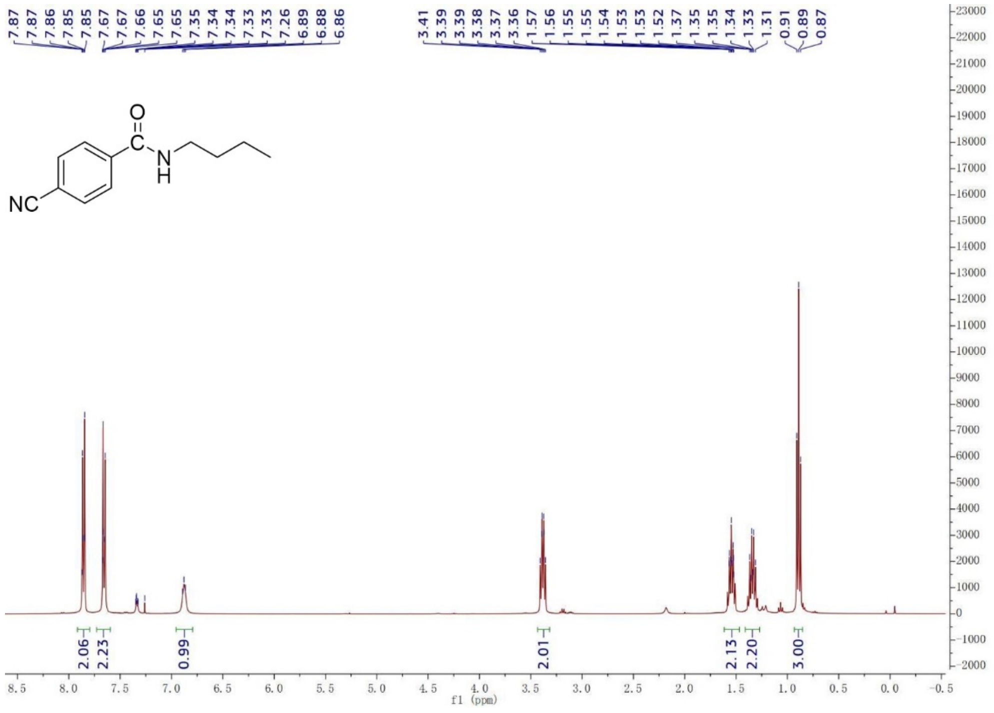

^{1}H NMR spectrum of product **10** in $CDCl_3$

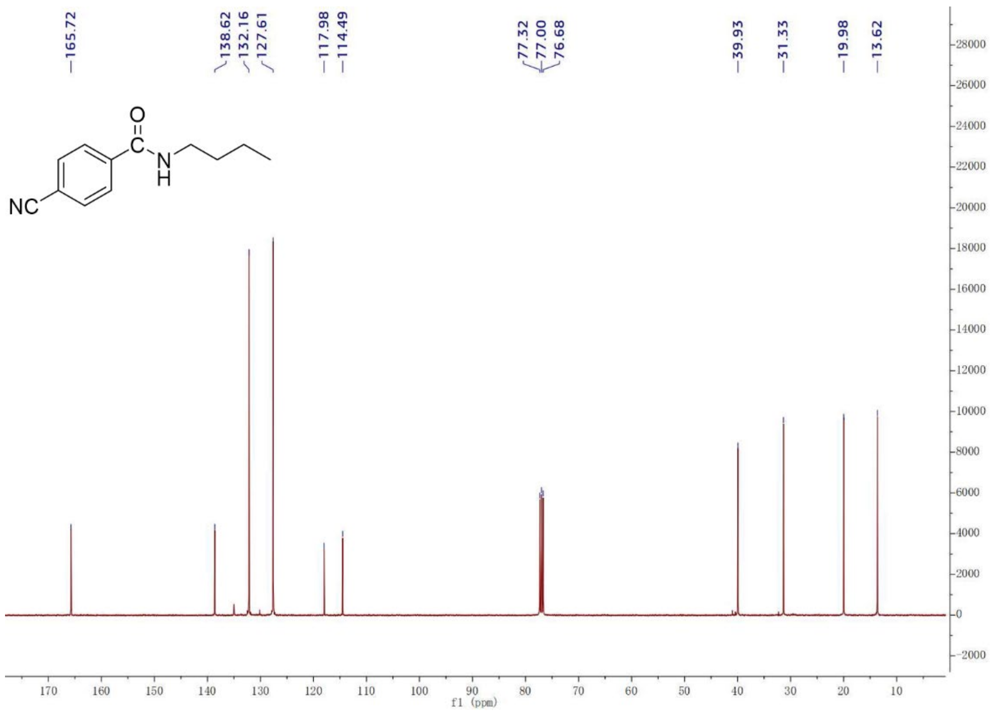

^{13}C NMR spectrum of product **10** in $CDCl_3$

11. (4-methoxyphenyl) (phenyl) methanone

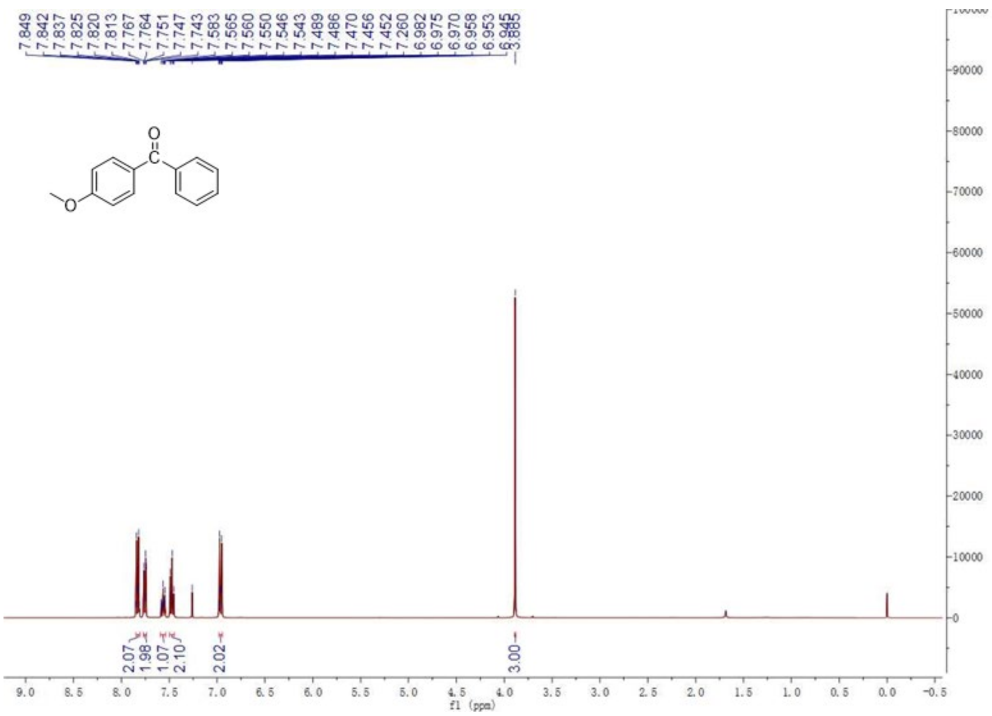

^{1}H NMR spectrum of product **11** in $CDCl_3$

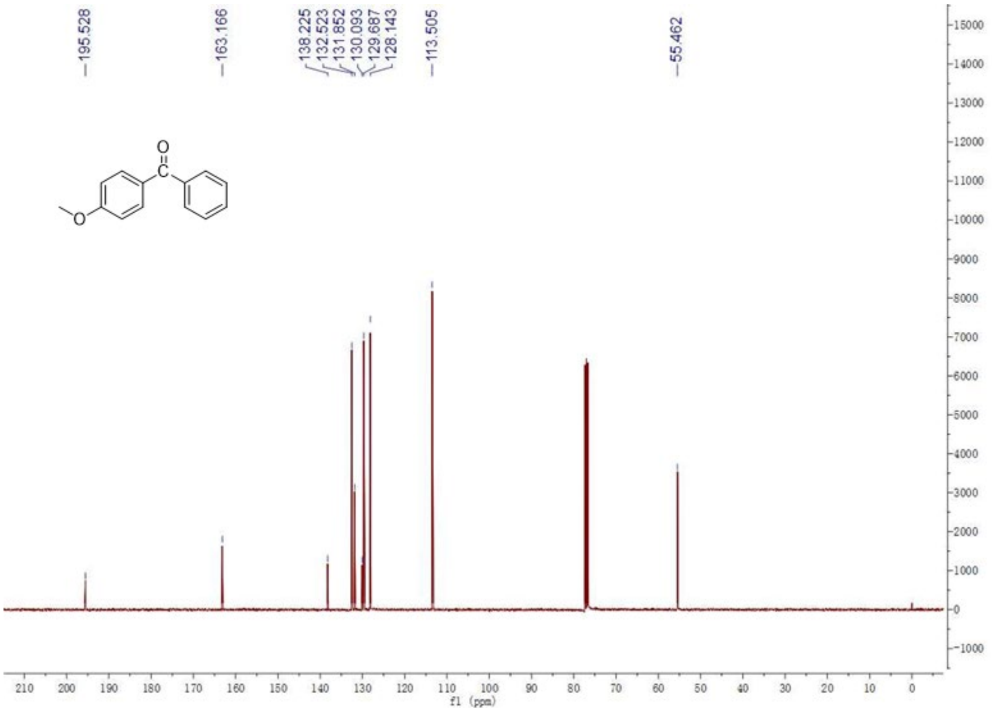

^{13}C NMR spectrum of product **11** in $CDCl_3$

12. Ethyl 4-methoxybenzoate

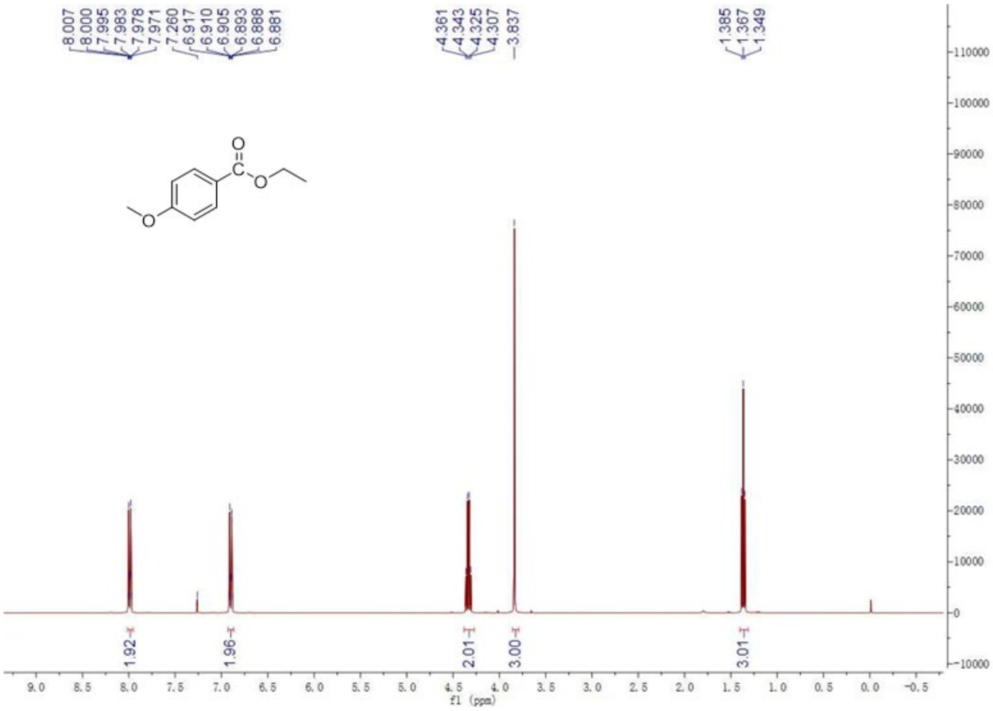

^{1}**H NMR** spectrum of product **12** in $CDCl_3$

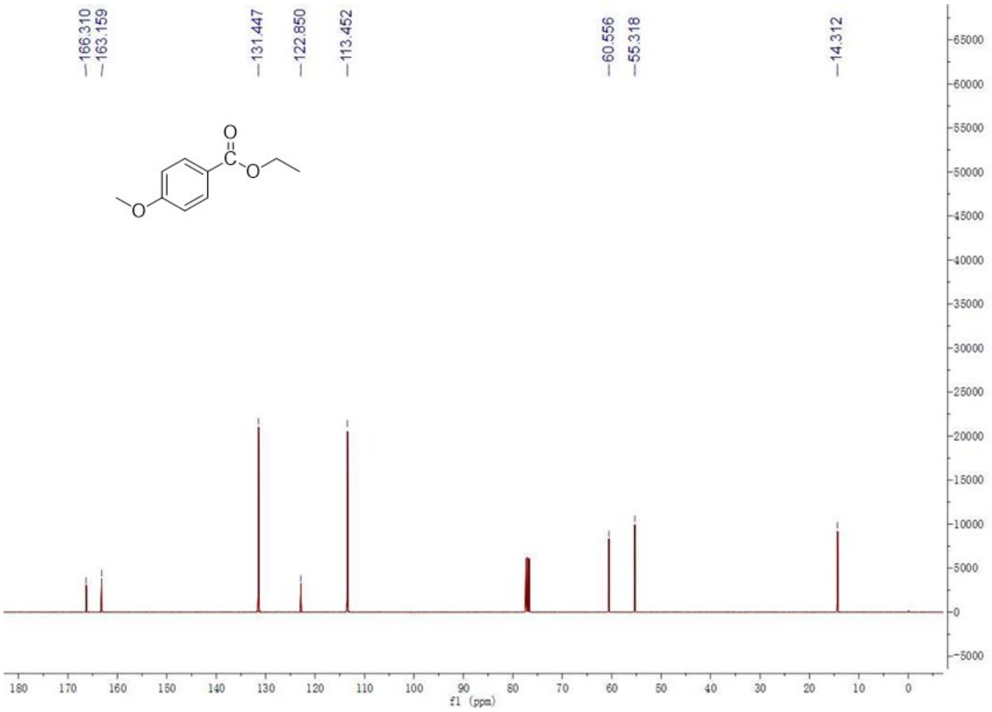

^{13}**C NMR** spectrum of product **12** in $CDCl_3$

13. phenyl 4-methoxybenzoate

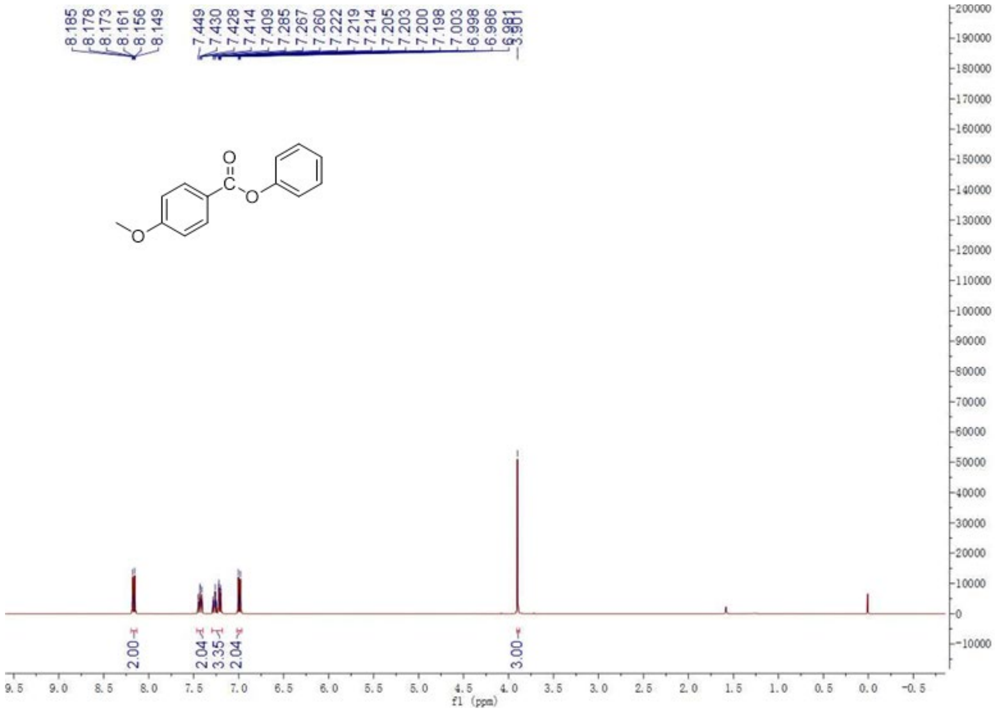

¹H NMR spectrum of product **13** in $CDCl_3$

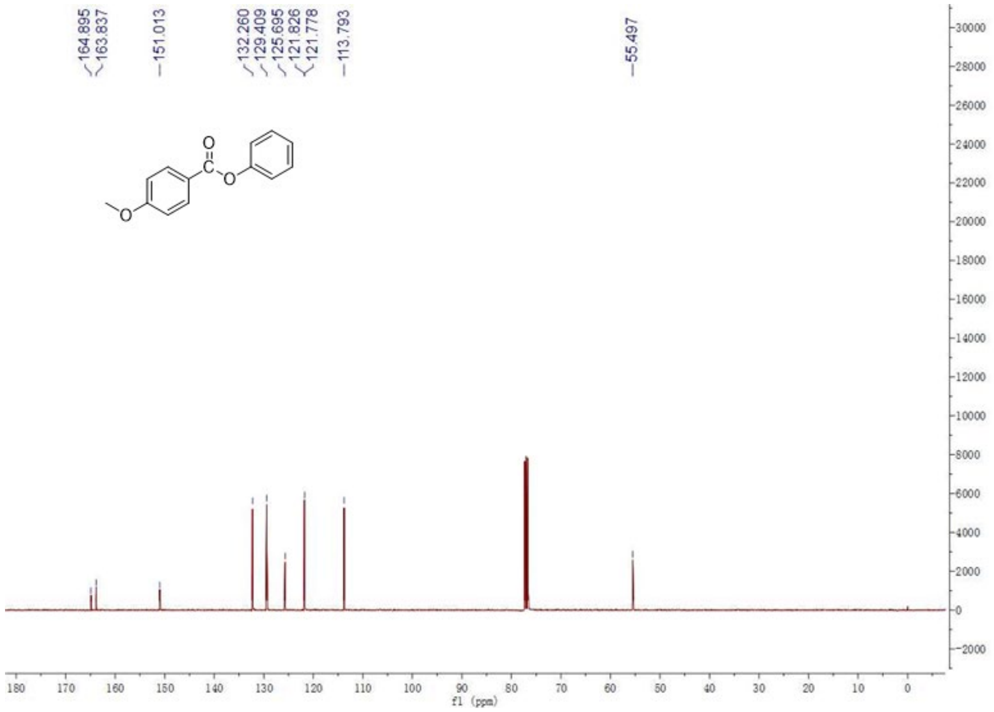

¹³C NMR spectrum of product **13** in $CDCl_3$

CheckCIF / PLATON report:

Structure factors have been supplied for datablock(s) NNU-55-Co

THIS REPORT IS FOR GUIDANCE ONLY. IF USED AS PART OF A REVIEW PROCEDURE FOR PUBLICATION, IT SHOULD NOT REPLACE THE EXPERTISE OF AN EXPERIENCED CRYSTALLOGRAPHIC REFEREE.

No syntax errors found. CIF dictionary Interpreting this report

Datablock: NNU-55-Co

Complexes

Bond precision	C-C = 0.0052 Å	Wavelength=0.71073	
Cell	a = 11.097(3)	b = 11.097(3)	c = 12.381(4)
	alpha = 90	beta = 90	gamma = 90
Temperature	296 K		
	Calculated		Reported
Volume	1524.6(10)		1524.6(10)
Space group	*P*4/*n*		*P*4/*n*
Hall group	-*P*4a		-*P*4a
Moiety formula	2($C_{13}H_8Co_{1.50}N_6O_2$), $C_2H_3O_2$		$C_{26}H_{16}Co_3N_{12}O_4$, $C_2H_3O_2$
Sum formula	$C_{28}H_{19}Co_3N_{12}O_6$		$C_{28}H_{19}Co_3N_{12}O_6$
Mr	796.34		796.34
Dx (g cm^{-3})	1.735		1.735
Z	2		2
Mμ (mm^{-1})	1.678		1.678
F (000)	800.0		800.0
F (000’)	802.33		
h,k,l (max)	12,12,14		12,12,14
Nref	1271		1264
T_{min},T_{max}	0.846, 0.846		0.687, 0.745
T_{min}’	0.846		

Correction method= # Reported T Limits: T_{min}=0.687 T_{max}=0.745

AbsCorr = NONE

Data completeness = 0.994 Theta(max) = 24.404

R(reflections) = 0.0349 (1014)	wR_2(reflections) = 0.0841(1264)
S = 1.064 Npar = 126	

There is no alert level A and B error.

Supplementary References

1. Sheldrick, G., SHELXT - Integrated space-group and crystal-structure determination. *Acta Crystallogr. A*. **71**, 3-8 (2015).
2. Sheldrick, G. Crystal structure refinement with SHELXL. *Acta Crystallogr. C*. **71**, 3-8(2015).
3. Dolomanov, O. V., Bourhis, L. J., Gildea, R. J., Howard, J. A. K., Puschmann, H., OLEX2: a complete structure solution, refinement and analysis program. *J. Appl. Crystallogr*. **42**, 339-341 (2009).
4. Alexandrov, E. V., Blatov, V. A., Kochetkov, A. V., Proserpio, D. M., Underlying nets in three-periodic coordination polymers: topology, taxonomy and prediction from a computer-aided analysis of the Cambridge Structural Database. *CrystEngComm*. **13**, 3947-3958 (2011).
5. F. Neese, *WIRES Comput. Mol. Sci.* **2**, 73-78 (2012).
6. A. Schäfer, C. Huber and R. Ahlrichs, *J. Chem. Phys.* **100**, 5829, (1994).
7. F. Weigend, *Phys. Chem. Chem. Phys.* **8**, 1057-1065, (2006).
8. S. Grimme, J. Antony, S. Ehrlich and H. Krieg, *J. Chem. Phys.* **132**, 154104, (2010).
9. A. A. Peterson, F. Abild-Pedersen, F. Studt, J. Rossmeisl and J. K. Nørskov, *Energy Environ. Sci.* **3**, 1311-1315, (2010).
10. Basnayake SA, Su J, Zou X, Balkus KJ. Carbonate-based Zeolitic Imidazolate Framework for highly selective CO_2 capture. *Inorg. Chem.* **54**, 1816-1821 (2015).
11. Zhang H, et al. Efficient Visible-Light-Driven Carbon Dioxide Reduction by a Single-Atom Implanted Metal–Organic Framework. *Angewandte Chemie International Edition*. **55**, 14310-14314 (2016).
12. Wang X-K, et al. Monometallic Catalytic Models Hosted in Stable Metal–Organic Frameworks for Tunable CO_2 Photoreduction. *ACS Catal*. **9**, 1726-1732 (2019).
13. Chen E-X, et al. Acid and Base Resistant Zirconium Polyphenolate-Metalloporphyrin Scaffolds for Efficient CO2 Photoreduction. *Adv Mater*. **30**, 1704388 (2018).
14. Zhao J, et al. A hexanuclear cobalt metal–organic framework for efficient CO_2 reduction under visible light. *J. Mater. Chem. A*. **5**, 12498-12505 (2017).

15. Zhong W, et al. A Covalent Organic Framework Bearing Single Ni Sites as a Synergistic Photocatalyst for Selective Photoreduction of CO_2 to CO. *J Am Chem Soc*. **141**, 7615-7621 (2019).
16. Hong D, Tsukakoshi Y, Kotani H, Ishizuka T, Kojima T. Visible-Light-Driven Photocatalytic CO_2 Reduction by a Ni(II) Complex Bearing a Bioinspired Tetradentate Ligand for Selective CO Production. *J Am Chem Soc*. **139**, 6538-6541 (2017).
17. Huang C, et al. Carbon-doped BN nanosheets for metal-free photoredox catalysis. *Nat Commun*. **6**, 7698 (2015).
18. Zhang H-X, et al. Isolated Square-Planar Copper Center in Boron Imidazolate Nanocages for Photocatalytic Reduction of CO_2 to CO. *Angewandte Chemie International Edition*. **58**, 11752-11756 (2019).
19. Jang, Doo Ok. Application of Cl_3CCONH_2/PPh_3 towards the Synthesis of Bioactive Amides. *Bull. Korean. Chem. Soc.* ***30***, 2066–2070 (2009).
20. Wetzel A, Ehrhardt V, Heinrich MR. Synthesis of Amino- and Hydroxybiphenyls by Radical Chain Reaction of Arenediazonium Salts. *Angew. Chem. Int. Ed*. **47**, 9130-9133 (2008).
21. Nordeman P, Odell LR, Larhed M. Aminocarbonylations employing $Mo(CO)_6$ and a bridged two-vial system: allowing the use of nitro group substituted aryl iodides and aryl bromides. *J. Org. Chem*. **77**, 11393-11398 (2012).
22. Kalkhambkar RG, Waters SN, Laali KK. Highly efficient synthesis of amides via Ritter chemistry with ionic liquids. *Tetrahedron Lett*. **52**, 867-871 (2011).
23. Nordstrøm LU, Vogt H, Madsen R. Amide Synthesis from Alcohols and Amines by the Extrusion of Dihydrogen. *J. Am. Chem. Soc*. **130**, 17672-17673 (2008).
24. He X, Cao Y, Lang X-D, Wang N, He L-N. Integrative Photoreduction of CO_2 with Subsequent Carbonylation: Photocatalysis for Reductive Functionalization of CO_2. *ChemSusChem*. **11**, 3382-3387 (2018).
25. Yu C, Özkaya B, Patureau FW. Electro-Oxidative Selective Esterification of Methylarenes and Benzaldehydes. *Chem. Eur. J.* **27**, 3682-3687 (2021).
26. Wang T, et al. Transition-Metal-Free DMAP-Mediated Aromatic Esterification of Amides with Organoboronic Acids. *Eur. J. Org. Chem.* **2021**, 3274-3277 (2021).
